# Effects of taxon sampling and tree reconstruction methods on phylodiversity metrics

**DOI:** 10.1002/ece3.5425

**Published:** 2019-08-21

**Authors:** Johanna R. Jantzen, William M. Whitten, Kurt M. Neubig, Lucas C. Majure, Douglas E. Soltis, Pamela S. Soltis

**Affiliations:** ^1^ Florida Museum of Natural History University of Florida Gainesville FL USA; ^2^ Department of Biology University of Florida Gainesville FL USA; ^3^ Department of Plant Biology Southern Illinois University of Carbondale Carbondale IL USA

**Keywords:** community phylogenetics, phylodiversity, phylogenetic diversity, phylogeny reconstruction methods, taxon sampling

## Abstract

1. The amount and patterns of phylodiversity in a community are often used to draw inferences about the local and historical factors affecting community assembly and can be used to prioritize communities and locations for conservation. Because measures of phylodiversity are based on the topology and branch lengths of phylogenetic trees, which are affected by the number and diversity of taxa in the tree, these analyses may be sensitive to changes in taxon sampling and tree reconstruction methods.
2. To investigate the effects of taxon sampling and tree reconstruction methods on measures of phylodiversity, we investigated the community phylogenetics of the Ordway‐Swisher Biological Station (Florida), which is home to over 600 species of vascular plants. We studied the effects of (a) the number of taxa included in the regional phylogeny; (b) random versus targeted sampling of species to assemble the regional species pool; (c) including only species from specific clades rather than broad sampling; (d) using trees reconstructed directly for the taxa under study compared to trees pruned from a larger reconstructed tree; and (e) using phylograms compared to chronograms.
3. We found that including more taxa in a study increases the likelihood of observing significantly nonrandom phylogenetic patterns. However, there were no consistent trends in the phylodiversity patterns based on random taxon sampling compared to targeted sampling, or within individual clades compared to the complete dataset. Using pruned and reconstructed phylogenies resulted in similar patterns of phylodiversity, while chronograms in some cases led to significantly different results from phylograms.
4. The methods commonly used in community phylogenetic studies can significantly impact the results, potentially influencing both inferences of community assembly and conservation decisions. We highlight the need for both careful selection of methods in community phylogenetic studies and appropriate interpretation of results, depending on the specific questions to be addressed.

## 1 INTRODUCTION

The field of community phylogenetics uses patterns of phylodiversity to understand community assembly and the coexistence of related species, incorporating a phylogenetic framework into the study of community ecology (Ackerly, 2003; Cavender‐Bares, Kozak, Fine, & Kembel, 2009; Webb, 2000; Webb, Ackerly, McPeek, & Donoghue, 2002). Recent studies have investigated patterns of community phylogenetic structure in diverse lineages including vertebrates (e.g., Gómez, Bravo, Brumfield, Tello, & Cadena, 2010; Patrick & Stevens, 2016), invertebrates (e.g., Lessard, Fordyce, Gotelli, & Sanders, 2009; Saito, Valente‐Neto, Rodrigues, de Oliveira Roque & Siqueira, 2016), algae (e.g., Fritschie, Cardinale, Alexandrou, & Oakley, 2014), zooplankton (e.g., Gianuca et al., 2017), and vascular plants (e.g., Kembel & Hubbell, 2006; Willis et al., 2010). These studies rely on measures of phylodiversity, a quantification of the evolutionary history represented by the taxa in a given community, based on the branches connecting these taxa on a regional phylogeny, often referred to as “phylogenetic diversity” (Faith, 1992). We refer to this concept as “phylodiversity” to distinguish it from Faith's phylogenetic diversity (PD), one specific index of phylodiversity. Since phylodiversity was first described, numerous indices have been developed to quantify the phylodiversity represented on trees; recent reviews highlight the differences and similarities between indices and describe their applications in different fields (Cadotte et al., 2010; Miller, Farine, & Trisos, 2017; Scheiner, Kosman, Presley, & Willig, 2017; Tucker et al., 2017; Vellend, Cornwell, Magnuson‐Ford, & Mooers, 2011). We focused on the impact of methods on phylodiversity as it relates to community structure using indices commonly used in community phylogenetics: NRI and NTI. A variety of factors may influence estimates of the magnitude and patterns of phylodiversity, such as the species composition of the regional species pool; yet, the extent to which many variables influence phylogenetic patterns and their interpretation is unclear. In this paper, we test five questions related to this gap in understanding how taxon sampling and tree reconstruction methods can affect estimated patterns of phylodiversity, focusing on metrics that are commonly used to understand community structure.

Identifying phylogenetic patterns in a community depends on comparing measures of phylodiversity to null models to determine if taxa in a given community are a nonrandom draw from across the phylogeny. These nonrandom patterns, namely phylogenetic clustering and phylogenetic overdispersion, are often interpreted as evidence of habitat filtering or competitive exclusion (Webb et al., 2002), respectively, although the assumptions underlying these interpretations have been called into question (e.g., Burns & Strauss, 2011; Fritschie et al., 2014; Gerhold, Cahill, Winter, Bartish, & Prinzing, 2015; Godoy, Kraft, & Levine, 2014). Correctly inferring community assembly processes from phylogenetic patterns is dependent on knowing whether functional trait diversity can be represented by phylodiversity (i.e., whether traits responsible for coexistence or competitive exclusion are evolutionarily conserved or convergent; Cadotte, Cavender‐Bares, Tilman, & Oakley, 2009; Cavender‐Bares et al., 2009). The importance of understanding the effects of these underlying assumptions, and complicating factors such as issues of spatial and temporal scale, on the interpretation of phylodiversity patterns has been previously addressed (e.g., Cadotte et al., 2009; Cavender‐Bares et al., 2009; Vamosi, Heard, Vamosi, & Webb, 2009), so we will not discuss these ideas further here.

Community phylogenetic patterns can be identified based on measures of phylodiversity for a community of coexisting species, assembled from a regional species pool, which comprises all species potentially able to colonize a site (Cornell & Harrison, 2014). The calculation of phylodiversity in a given community is therefore based on the phylogeny of the regional species pool. Thus, the identification and interpretation of phylogenetic patterns may be affected by the choice of taxa for study and the methodological decisions involved in reconstructing the regional phylogeny. However, the effect of different taxon sampling strategies and tree reconstruction methods on the calculation of phylodiversity metrics and the identification of phylogenetic patterns is not well understood. Trees for phylodiversity studies are typically reconstructed from a few molecular loci (e.g., Pei et al., 2011; Schmidt‐Lebuhn, Knerr, Miller, & Mishler, 2015), although genomic data have been used as well (Kellar, Ahrendsen, Aust, Jones, & Pires, 2015). In many cases, community phylogenies are pruned from trees based on much larger sets of species that were reconstructed for other studies (e.g., Lessard et al., 2009; Patrick & Stevens, 2016; Pyron & Burbrink, 2014). When molecular phylogenies are unavailable, trees may be reconstructed from taxonomic information, or constructed as supertrees from other published phylogenies (e.g., Brunbjerg, Borchsenius, Eiserhardt, Ejrnaes, & Svenning, 2012; Hinchliff et al., 2015; Willis et al., 2010). Moreover, some studies use phylograms, with branch lengths in units of substitutions per site, representing the amount of evolutionary change (e.g., Cavender‐Bares, Ackerly, Baum, & Bazzaz, 2004; Schmidt‐Lebuhn et al., 2015; Saito et al., 2016), while others use chronograms, with branch lengths in units of evolutionary time (e.g., Araya et al., 2012; Kembel & Hubbell, 2006; Willis et al., 2010). The effect of these alternative tree reconstruction methods and of using phylograms versus chronograms on estimated patterns of phylodiversity is unclear.

Additionally, rather than including all taxa found in the region or community, studies often sample only a small subset of taxa. The sampling strategy for a given study may focus on the most species‐rich clades (e.g., Kellar et al., 2015), the taxa that are either most ecologically representative or ecologically dominant (e.g., Araya et al., 2012), or the taxa that are relevant to a specific research question (e.g., Cavender‐Bares et al., 2004; Mishler et al., 2014; Münkemüller et al., 2014). From a practical standpoint, studies may focus on the taxa that have the most data available for the region under study (e.g., Schmidt‐Lebuhn et al., 2015), potentially biasing the study toward well‐sampled species. Different sampling strategies can be justified based on the questions being asked, but the effect that alternative sampling strategies may have on observed patterns of phylodiversity has yet to be determined (Kellar et al., 2015; Münkemüller et al., 2014; Vamosi et al., 2009).

In this paper, we test how alternative taxon sampling strategies, different tree reconstruction methods, and the representation of phylogenies as phylograms versus chronograms affect estimates of phylodiversity using an empirical dataset. Although a few recent studies have independently addressed aspects of some of these issues (e.g., Allen et al., 2019; Elliott, Knerr, & Schmidt‐Lebuhn, 2018; Park, Worthington, & Xi, 2018), many questions remain. We therefore use an empirical approach to explore novel aspects of phylodiversity estimation by addressing five questions:

1. What effects do the number and proportion of taxa in the regional phylogeny have on estimates of phylodiversity?
2. For assembling the regional species pool, how does random versus targeted taxon sampling affect patterns of phylodiversity?
3. Do patterns of phylodiversity vary among clades, and if so, how?
4. Do phylodiversity estimates derived from regional phylogenies pruned from a larger phylogeny differ from those based on regional phylogenies built specifically for the analysis?
5. Do measures of phylodiversity differ when based on chronograms versus phylograms?

Taken together, these lines of enquiry enable us to assess the robustness of phylodiversity metrics to differences in methodologies.

## 2 MATERIALS AND METHODS

### 2.1 Geographic site

**Figure 1:**
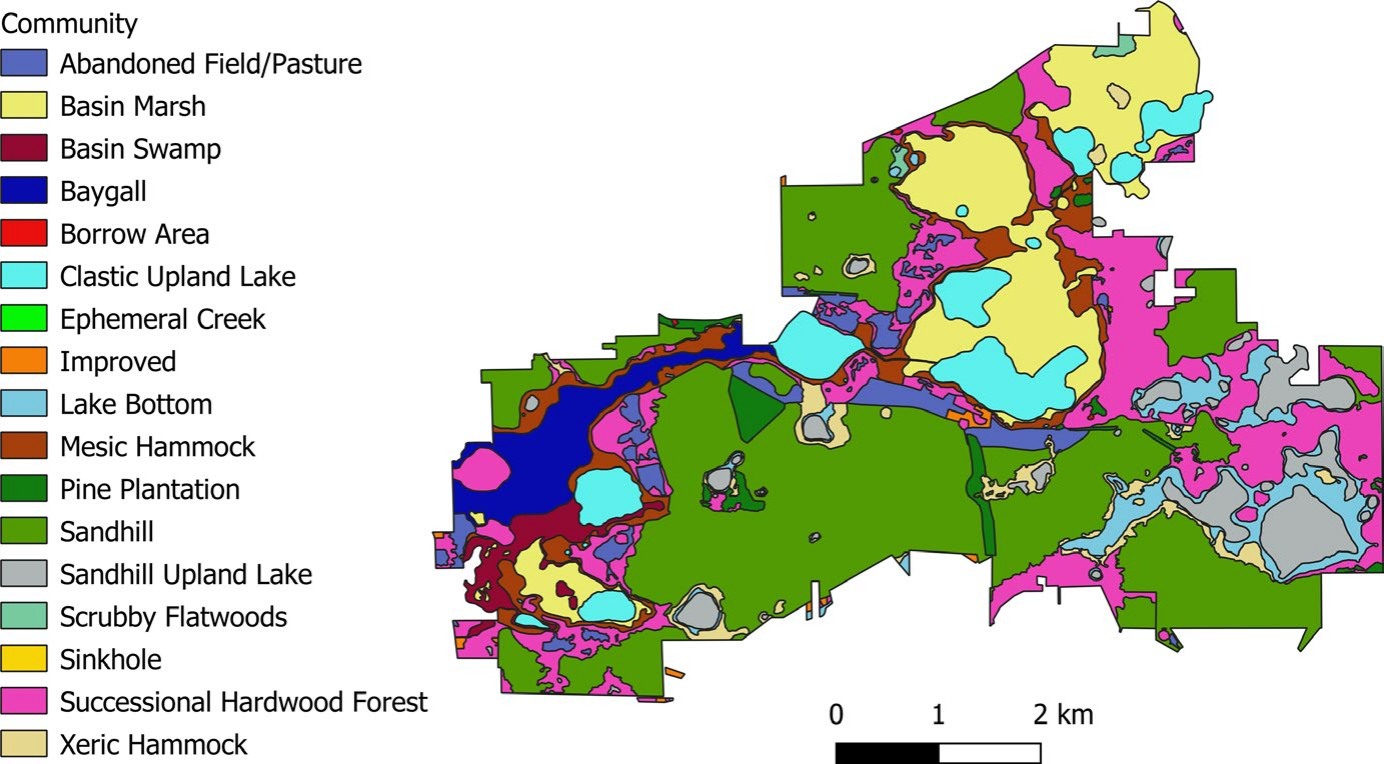
Map of the FNAI vegetation communities of the Ordway‐Swisher Biological Station (UF/IFAS; redrawn from http://ordway-swisher.ufl.edu/PlantCommunities.aspx)

We evaluated the potential impacts of taxon sampling strategies and tree reconstruction methods on estimates of phylodiversity at the Ordway‐Swisher Biological Station (OSBS). The OSBS site is located in north‐central Florida (Putnam County), at 29°41′N and 82°0′W, and covers more than 3,840 hectares. The OSBS is ecologically diverse, with 11 communities described by the Florida Natural Areas Inventory (FNAI, 2010) and three altered landcover types and has been the subject of a floristic inventory and barcoding project (unpublished data, L. C. Majure et al.; Figure 1). The OSBS is managed using prescribed burns, and some areas have been subject to other anthropogenic disturbances such as road building and pine plantations.

### 2.2 Taxon sampling and DNA sequencing

The vascular flora of OSBS was documented by collections of several botanists (L. C. Majure, K. M. Neubig, W. S. Judd, and W. M. Whitten) over a several‐year period (2014–2016); voucher specimens and digital images are deposited in the herbarium of the Florida Museum of Natural History (FLAS; https://www.floridamuseum.ufl.edu/herbarium/cat/imagesearch.asp?srchproject=OS). We focused on vascular plants, utilizing a DNA barcoding dataset of *rbcL* and *matK* sequences for 572 of the ca. 680 species (~84%) found at OSBS (Appendix A: Tables A1 and S26), with each species represented by a single individual. DNA extractions were made from pulverized silica‐dried tissues incubated in a CTAB‐based buffer and then purified with isoamyl alcohol/chloroform followed by a silica‐column purification (Neubig et al., 2014). PCR and sequencing methods followed the Smithsonian barcoding protocol for *rbcL* and *matK* (CBOL Plant Working Group, 2009; Dunning & Savolainen, 2010; Ford et al., 2009; Kress et al., 2009; Levin et al., 2003; Yu, Xue, & Zhou, 2011).

Contaminants and misidentified taxa were removed by visual inspection following preliminary phylogenetic analysis using the methods described below. Sequence identity was also verified by conducting BLAST searches for all sequences to check for contaminants. 2015 was used to reconcile species names (Boyle et al., 2013; The Taxonomic Name Resolution Service, 2015, and associated databases: https://Tropicos.org, 2014; The PLANTS Database, 2015; Global Compositae Checklist, 2009; The Taxonomy Project, 2003; The Plant List, 2013; and International Legume Database and Information Service, 2013) using the default parameters and manually checking the output for ambiguous matches and synonyms. This molecular dataset provides our estimate of the community‐level phylogeny for this site and represents the complete dataset from which subsets were taken for comparison.

### 2.3 Sequence alignment and phylogeny reconstruction

Sequences were aligned using ClustalW with manual adjustments (Thompson, Higgins, & Gibson, 1994), and alignments were visually inspected before phylogenetic analysis. The *rbcL* alignment includes 562 aligned base pairs (bp), and the *matK* alignment includes 1,223 aligned bp, with missing data scored as “?”. These two plastid regions were concatenated to produce a final alignment of 1,785 characters. The alignment matrix is deposited on Dryad (doi:10.5061/dryad.5m9n159).

Preliminary maximum‐likelihood (ML) analyses of the 572‐taxon dataset produced some bipartitions in the backbone of the tree that conflict with currently accepted topologies (e.g., APG IV, 2016; Wickett et al., 2014), primarily due to a lack of informative characters for those clades. Although nearly comprehensive for vascular plants of OSBS, the broad taxon sampling did not allow for complete resolution of all relationships, particularly when coupled with the relatively small number of characters in this dataset and the conserved nature of these plastid genes. As a result, six constraints were used to enforce the currently accepted branching order at deeper nodes, as has been done in other studies (e.g., Allen et al., 2019). These constraints were tailored to each dataset used for tree reconstruction so that each resulting phylogeny would not conflict with the expected backbone branching order (Soltis et al., 2011; APG IV, 2016; Wickett et al., 2014). Using phylogenetic constraints is a robust way of ensuring that the backbone topology is consistent with more rigorous phylogenetic studies with broader taxon sampling, while allowing branch lengths and unconstrained bifurcations to vary. These constraints should not bias the results of our study, as we use very few constraints (only six out of between 98 (100‐taxon subset) and 571 (complete dataset) bifurcations were constrained), and the methods being tested in this study should be impacted equally by the improved backbone topology. As was also found by Allen et al. (2019), these barcoding loci produced a remarkably well‐resolved tree with only minor deviations from the expected topology (e.g., Soltis et al., 2011; APG IV, 2016; Wickett et al., 2014); the differences from the expected tree are primarily in phylogenetic relationships that have been notoriously difficult to resolve. Any lack of resolution or short branch lengths resulting from the use of slowly evolving barcoding loci are not likely to affect the phylogenetic patterns, as they contribute much less to the overall tree length than more well‐supported, longer branches (Allen et al., 2019).

Maximum‐likelihood (ML) analyses were carried out in RAxML using the GTRGAMMA model of nucleotide substitution and separate partitions for *rbcL* and *matK* (Stamatakis, 2014). For each analysis, a thorough best tree search was run from a random starting tree with 1,000 fast bootstrap replicates. The ML tree for the complete dataset was rooted using the lycophytes, based on well‐supported relationships among vascular plants (e.g., Wickett et al., 2014). For individual trees reconstructed for subsets of taxa, because the presence of one or more lycophytes in the subset tree was not guaranteed, an R script was written to check for the presence of each subsequently basal node on the tree from the complete dataset and to root the subset trees on the basal most node included (R Core Team, 2013).

For comparing chronograms to phylograms, the best ML tree with the highest likelihood score was made ultrametric using the program TreePL (Smith & O'Meara, 2012). Calibration points were taken from Bell, Soltis, and Soltis (2010) (Appendix B: Table B1), and smoothing parameters were designated based on cross‐validation. For questions that used pruned trees (either phylograms or chronograms), the R package “ape” was used to drop tips from the larger tree based on the complete dataset using the drop.tip function (Paradis, Claude, & Strimmer, 2004).

### 2.4 Regional species pool and community data

The complete regional species pool was considered to be composed of all of the vascular plant taxa documented at OSBS. We limited our taxonomic scope to the taxa found in the region of interest (OSBS) as is typically done in community phylogenetic studies. For the questions we posed that test the effects of different sampling strategies, the number and composition of species in the regional species pool was manipulated, resulting in regional species pools of different sizes and species richness. Within each regional species pool, phylodiversity measures were calculated for the 14 different communities. Although it was the regional species pool that was manipulated in this study, the number and composition of species in a community is highly correlated with, and directly linked to, the number and composition of taxa in the regional species pool.

We used the individual communities and landcover types (collectively referred to hereafter as communities) within the OSBS to investigate how methodological effects may influence phylodiversity patterns across different types of communities and to understand how consistent these phenomena are. These 14 communities are Abandoned Field/Pasture, Basin Marsh, Basin Swamp, Baygall, Clastic Upland Lake, Improved, Lake Bottom, Mesic Hammock, Pine Plantation, Sandhill, Sandhill Upland Lake, Scrubby Flatwoods, Successional Hardwood Forest, and Xeric Hammock (FNAI, 2010). These communities are best considered as habitat types rather than individual plots, as multiple patches of the same habitat type are scattered across the OSBS instead of a habitat type being confined to a single location to form a plot (Figure 1). This empirical dataset, with sequence data for species from multiple communities, provides a unique opportunity to characterize the effects of different methodologies in a natural system across different communities. Species were assigned to communities at the OSBS using the FNAI classification system (FNAI, 2010). GPS coordinates from voucher specimens were plotted onto a map of the FNAI communities at the OSBS in QGIS (QGIS Development Team, 2015). Because many species occur in more than one community, in addition to the single voucher specimen per species, additional species occurrence datasets were downloaded from https://www.neonscience.org/ and plotted onto the OSBS GIS map. Species were then assigned to communities. Assignments were edited to ensure that they were consistent with expectations based on both the FNAI community descriptions and expert taxonomic opinion (i.e., W. M. Whitten). Species that are known to be invasive (and actively removed by OSBS staff) or cultivated at an abandoned plant nursery at OSBS, and those that could not be unambiguously assigned to communities were omitted, resulting in our final dataset of presence/absence data for 572 native and naturalized species distributed across 14 communities. Most species were present in only one community (392 species), while several were found in many communities (2–3 communities = 120 species; 4–6 communities = 45 species; 7–10 communities = 13 species).

### 2.5 Indices

The choice of metric can affect the detection of phylodiversity patterns (Hardy, 2008; Kembel, 2009). Three widely used phylodiversity indices were chosen for this study based on their prevalence in the literature, the different aspects of phylodiversity that they capture, and how they may differ in their potential sensitivity to the methods examined in this study (Kellar et al., 2015; Scheiner et al., 2017). We calculated the standard effect sizes (SES) of Faith's PD (resulting in PD_SES_), mean pairwise distance (resulting in the inverse of net relatedness index or NRI), and mean nearest taxon distance (resulting in the inverse of nearest taxon index or NTI) by comparing observed values of phylodiversity to null models. In this study, the null models were represented by the same phylogenetic tree topology, branch lengths, and list of taxa as the observed phylogeny, but the positions of taxa at the tips were randomized with 1,000 replicates for each calculation. In other words, for each community phylogeny, the calculated phylodiversity value (PD, MPD, or MNTD) was compared with 1,000 null values to obtain the SES. Therefore, these indices (PD_SES_, −NRI, and −NTI) represent effect sizes rather than raw values of phylodiversity and are therefore comparable across datasets and phylogenies. Because many studies have been conducted using these indices and null models, it is important to assess how taxon sampling and tree reconstruction methods may influence estimates of phylodiversity as measured using these indices.

Measures of phylodiversity were calculated using the R package “picante” (Kembel et al., 2010). Each phylogeny was a rooted tree with branch lengths given either in substitutions per site (reconstructed and pruned ML phylograms) or evolutionary time (pruned chronograms). Results from these analyses were compared between communities to determine how consistent the effects of these methodologies may be across the 14 different community types. Because the output from “picante” is the inverse of Webb's NRI and NTI (Kembel et al., 2010; Webb, 2000), communities that are phylogenetically overdispersed have positive SES, communities that are phylogenetically clustered have negative SES, and communities that have no phylogenetic signal (i.e., where taxa are randomly distributed across the tree) have SES that are not significantly different from zero. Because these indices are calculated for each community as a fraction with respect to the regional dataset, these measures cannot be calculated in a meaningful way for the regional dataset itself, so comparisons were only conducted on the subsets, not for the complete dataset.

### 2.6 Study design

Our five questions were addressed as follows:

1. Number and proportion of taxa
  We compared results from the complete dataset of 572 taxa with those from random subsets of 100, 200, 300, 400, and 500 taxa, which might reflect varying sampling effort in generating a regional phylogeny. Because this question addresses sampling completeness, rather than sampling biases, these subsets were taken randomly from the complete dataset using a Biopython script (Cock et al., 2009), with 100 replicates for each subset size. The complete ML phylogeny was pruned, resulting in 100 replicates of pruned trees for each subset size. Phylodiversity indices were calculated for each community for each replicate tree for each subset size, resulting in a distribution of indices; in this analysis, all trees were represented as phylograms.
2. Random versus targeted sampling
  To test for sampling bias in the taxa that are chosen from the regional species pool, namely using targeted (i.e., nonrandom or balanced) sampling for assembling the regional species pool (i.e., the species at OSBS), the randomly sampled subsets from Question 1 were compared to nonrandomly sampled subsets with family representation in the subsets proportional to the family representation in the complete dataset. Species were sampled randomly from within each family with 100 replicates for each subset size (i.e., 89, 186, 328, 397, and 510 species; Table S13); subset sizes were designed to closely match those in Question 1 while keeping sampling proportional to family representation. This targeted sampling scheme was designed to mimic studies that sample the regional species pool according to the relative species richness within the regional species pool (e.g., Kellar et al., 2015). However, studies are often not explicit about decisions behind sampling methods; therefore, it is unclear whether our methods represent the majority of published studies. Our sampling scheme represents one example of a nonrandom sampling effort that is similar to a species richness‐based approach. Phylodiversity indices were then calculated for each community for these targeted subsets, and these effect sizes were compared to the phylodiversity effect sizes for the randomly sampled subsets calculated for Question 1. As for Question 1, all trees were represented as phylograms.
3. Individual clades
  We chose the six families (all of which are clades; APG IV, 2016) with highest species richness at OSBS (in ascending order: Fagaceae, Ericaceae, Cyperaceae, Fabaceae, Asteraceae, and Poaceae) and a more inclusive angiosperm clade, the rosids (a clade of approximately 90,000 species worldwide, Sun et al., 2016), and compared phylodiversity patterns among them. The ML phylogeny based on the complete dataset was pruned to produce seven trees, each representing one of these clades. Phylodiversity indices were calculated for the 14 communities for each tree and compared among clades and with the complete dataset. Where the phylodiversity pattern (clustering, overdispersion, or random) was consistent between an individual clade and the complete dataset, this was considered a match, while differences in patterns (e.g., clustering was found in an individual clade while no non‐random phylogenetic pattern was found in the complete dataset) were considered mismatches. Mismatches are further subdivided into “significant mismatches,” where one clade shows significant phylogenetic overdispersion while the other shows significant phylogenetic clustering, and “nonsignificant mismatches,” where one of the clades does not show a significant phylogenetic pattern but the other does. Matches were similarly subdivided, with “significant matches,” where both clades show a significant phylogenetic pattern, and “nonsignificant matches,” where both clades show a lack of significant pattern (i.e., random). Comparisons where there were no taxa from a given clade in the community were not considered (i.e., NA).
4. Pruned versus purpose‐built phylogenies
  Using the full tree and the same randomly sampled subsets described in Question 1, ML phylogenies were reconstructed for each subset size, and the three phylodiversity indices were calculated for each community. Due to difficulties resolving phylogenies with certain combinations of taxa, not all 100 replicates were run to completion for each subset. The calculated phylodiversity effect sizes for the reconstructed subset trees were compared to those calculated for the pruned subset phylogenies.
5. Phylograms versus chronograms
  The ML tree for the complete dataset was time‐calibrated (i.e., made ultrametric) and was then pruned to match the randomly sampled subsets from Question 1, resulting in 100 matching replicate chronograms and phylograms for each subset. Phylodiversity indices were then calculated for each community for these chronograms and compared to the phylodiversity effect sizes calculated in Question 1 for the phylograms.

## 3 RESULTS

Because the results for each question were consistent for NRI, NTI, and PD_SES_, only NRI, the most widely used index in community phylogenetics, is presented in the text; the results for the other indices are available in the Supporting Information.

### 3.1 Question 1: What effects do the number and proportion of taxa in the regional phylogeny have on estimates of phylodiversity?

**Figure 2:**
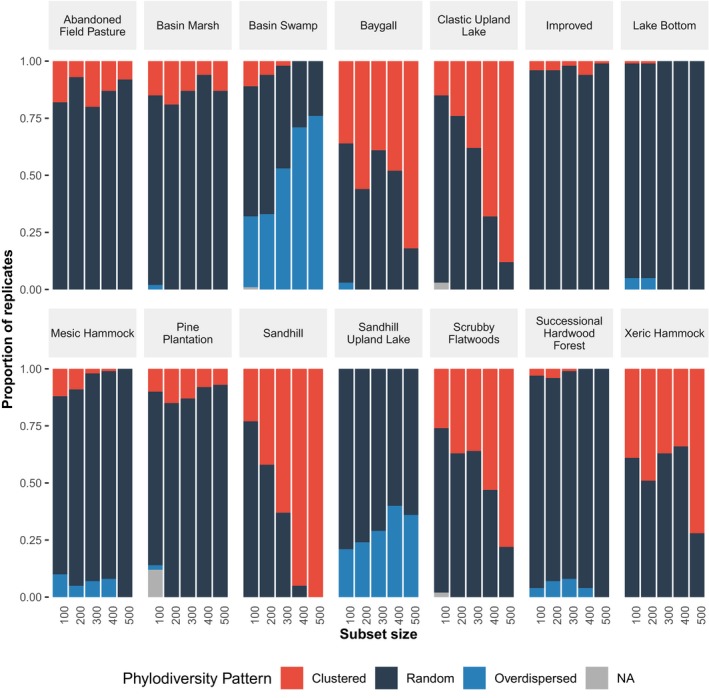
The effect of the number of taxa in the regional dataset on the detection of patterns of phylodiversity (i.e., clustered, overdispersed, or random). For each dataset size, 100 random replicates were drawn from the complete dataset, and NRI indices were calculated for each community for each replicate. For each community and dataset size, the proportions of replicates with a clustered, overdispersed, and random pattern sum to one

The number of taxa in the regional phylogeny can affect the likelihood of observing significantly non‐random phylogenetic patterns (Figure 2 and Tables S1–S6). For seven communities, the proportion of significant phylogenetic patterns increased with increasing numbers of taxa (overdispersion, 2 communities; clustering, 5 communities). In the remaining seven communities, the number of taxa in the regional phylogeny had little to no effect on the proportion of results that were significantly nonrandom. The communities that showed no effect of the number of taxa tended to have consistently lower proportions of significantly nonrandom results.

### 3.2 Question 2: For assembling the regional species pool, how does random versus targeted taxon sampling affect patterns of phylodiversity?

**Figure 3:**
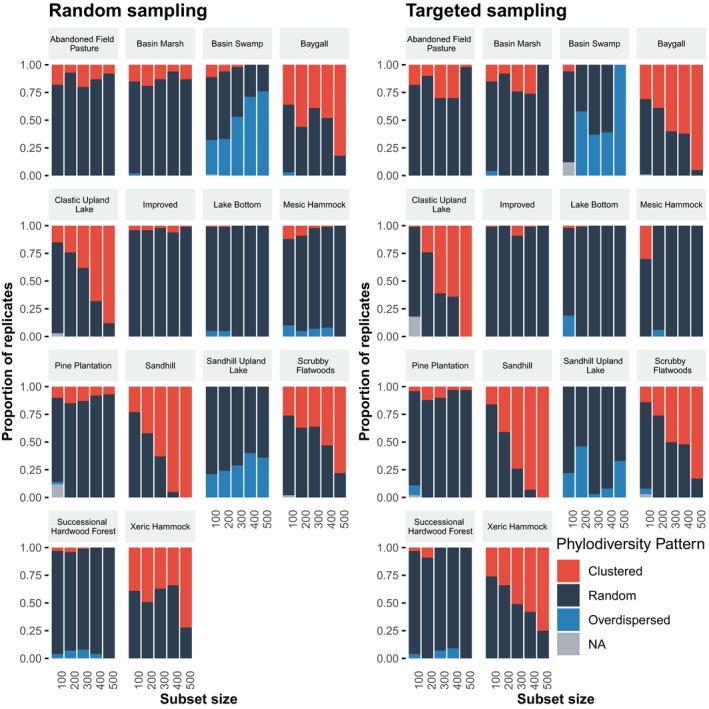
The effect of random (left) or targeted (right) sampling on the detection of patterns of phylodiversity (i.e., clustered, overdispersed, or random) across different dataset sizes. For each dataset size, 100 replicates were either randomly sampled from the complete dataset or sampled proportional to family representation in the complete dataset, and NRI indices were calculated for each community for each replicate. For each sampling strategy, community, and dataset size, the proportions of replicates with a clustered, overdispersed, and random pattern sum to one

Both targeted and random subsets showed an increase in the proportion of significantly nonrandom patterns with increasing numbers of taxa in approximately half of the communities (Figure 3 and Tables S7–S12). The random and targeted subsets resulted in similar proportions of significantly nonrandom results for most subset sizes for half the communities; however, in seven communities, the two types of subsets resulted in different proportions of significant phylogenetic patterns at one or more subset sizes (Figure 3). Also, there was no consistent pattern in how random or targeted subsets affected the phylogenetic patterns, as proportions of replicates that showed significant phylogenetic patterns were not consistently higher or lower for a given sampling strategy across different subset sizes.

### 3.3 Question 3: Do patterns of phylodiversity vary among clades, and if so, how?

**Figure 4:**
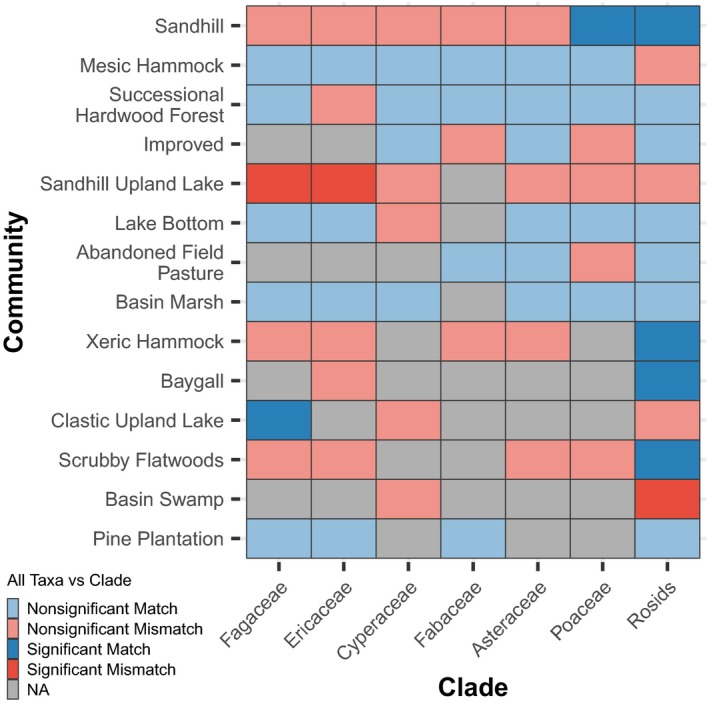
Comparison between the pattern of phylodiversity identified for a given clade compared to the complete dataset based on the calculation of NRI. Comparisons are classified as significant matches (the same significant phylogenetic pattern is found in an individual clade and in the complete dataset), nonsignificant matches (both an individual clade and the complete dataset show no significant pattern i.e., random), significant mismatches (different significant phylogenetic patterns are found in a clade and the complete dataset), nonsignificant mismatches (a significant phylogenetic pattern is found in either a clade or the complete dataset, but not in the other), and NA (taxa are too few to calculate or absent from the clade in a given community). The axes are sorted by increasing species richness in the complete dataset for both communities and clades

**Figure 5:**
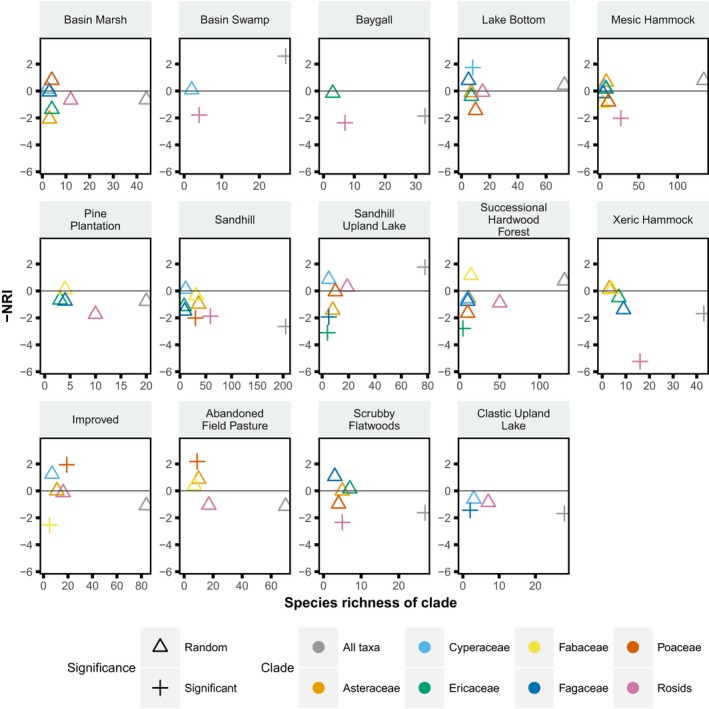
Values of NRI versus species richness for individual clades and the complete dataset for 14 communities. Significant phylogenetic patterns are indicated with a plus sign (+) while nonsignificant (random) patterns are indicated with a triangle (∆). Significant positive effect sizes are phylogenetically overdispersed while significant negative effect sizes are phylogenetically clustered

No consistent relationship was found between the phylodiversity patterns observed for individual clades and those found in the complete dataset (Figure 4). Several clades showed the same significant phylogenetic pattern as the overall dataset (e.g., rosids and Poaceae in the Sandhill community), while others showed a different significant phylogenetic pattern than in the overall dataset (e.g., Fagaceae and Ericaceae in the Sandhill Upland Lake). The majority of clades showed a nonsignificant match or mismatch, where one or both of the clades failed to identify a significant phylogenetic pattern. There was also no clear relationship between the phylodiversity of individual clades and the number of taxa represented in that clade, as some clades differed in numbers of taxa yet resulted in similar effect sizes for a given community (e.g., rosids and Fagaceae in the Successional Hardwood Forest) or had similar numbers of taxa but different effect sizes (e.g., Cyperaceae and Fagaceae in the Sandhill Upland Lake, or Poaceae and Fabaceae in the Sandhill; Figure 5).

### 3.4 Question 4: Do phylodiversity estimates derived from regional phylogenies pruned from a larger phylogeny differ from those based on regional phylogenies built specifically for the analysis?

**Figure 6:**
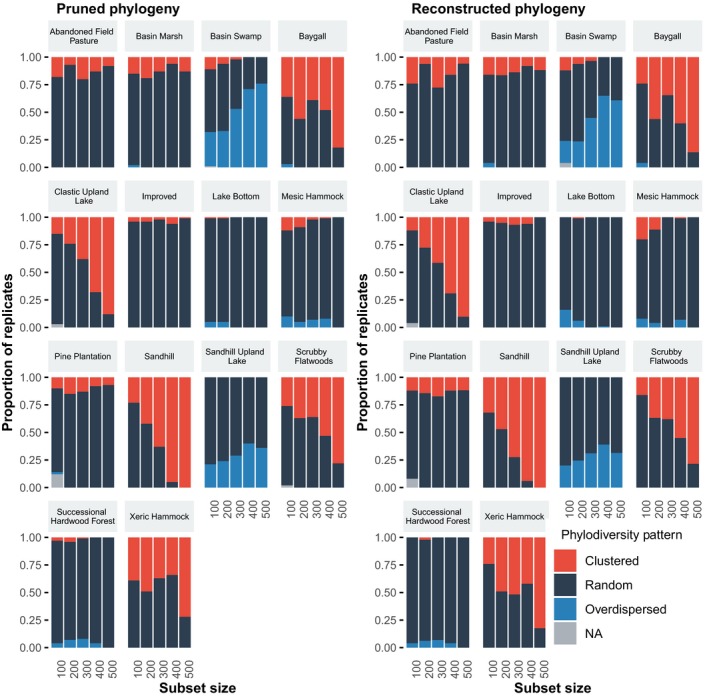
The effect of pruned phylogenies (left) and reconstructed phylogenies (right) on the detection of patterns of phylodiversity (i.e., clustered, overdispersed, or random) across different dataset sizes. For each dataset size, up to 100 replicates were either reconstructed directly from a randomly sampled set of sequences or pruned from the complete phylogeny to match the randomly sampled taxa, and NRI indices were calculated for each community for each replicate. For each tree reconstruction method, community, and dataset size, the proportions of replicates with a clustered, overdispersed, and random pattern sum to one

Measures of phylodiversity were, on average, not significantly different between the pruned and reconstructed phylogenies (Tables S14–S19), although values from individual replicates often differed based on the use of pruned versus reconstructed phylogenies, with some replicates showing large differences between the two methods. Despite this slight variability, the proportions of replicates showing significantly nonrandom patterns based on pruned phylogenies corresponded closely with the proportions found using reconstructed phylogenies (Figure 6). This pattern is consistent across subset sizes and both in communities that show an increase in proportion of nonrandom patterns with increasing subset size and in communities that are predominantly random.

### 3.5 Question 5: Do measures of phylodiversity differ when based on chronograms versus phylograms?

**Figure 7:**
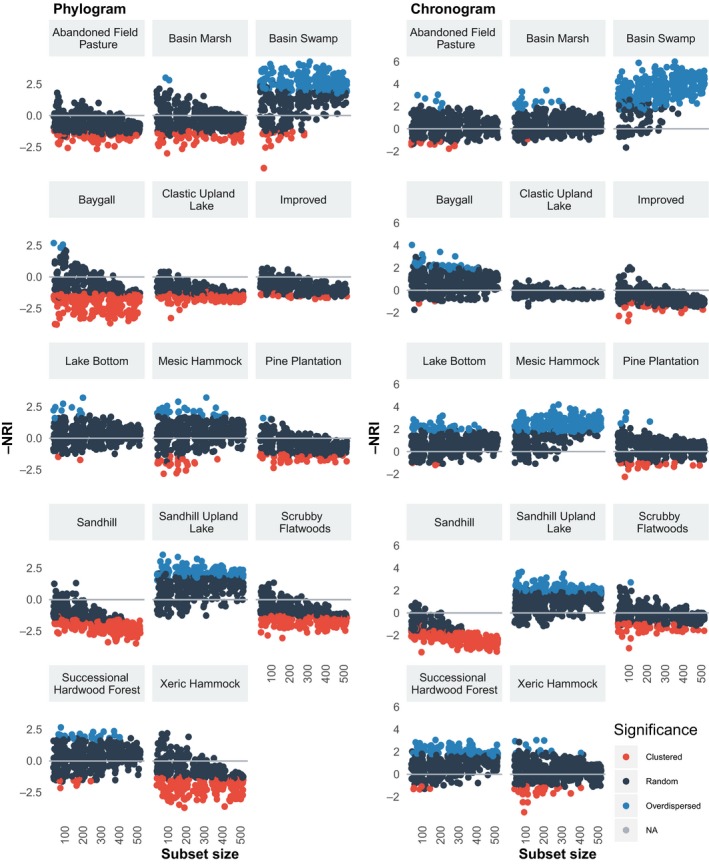
Scatterplot of the distribution of values of NRI for phylograms (left) and chronograms (right). For each of 100 randomly sampled replicates, a phylogram was pruned from the complete phylogram, and a chronogram was pruned from the time‐calibrated complete chronogram. NRI indices were calculated for each community for each replicate for these two tree types. Phylogenetic patterns are colored by clustered (red), random (dark gray), and overdispersed (blue)

**Figure 8:**
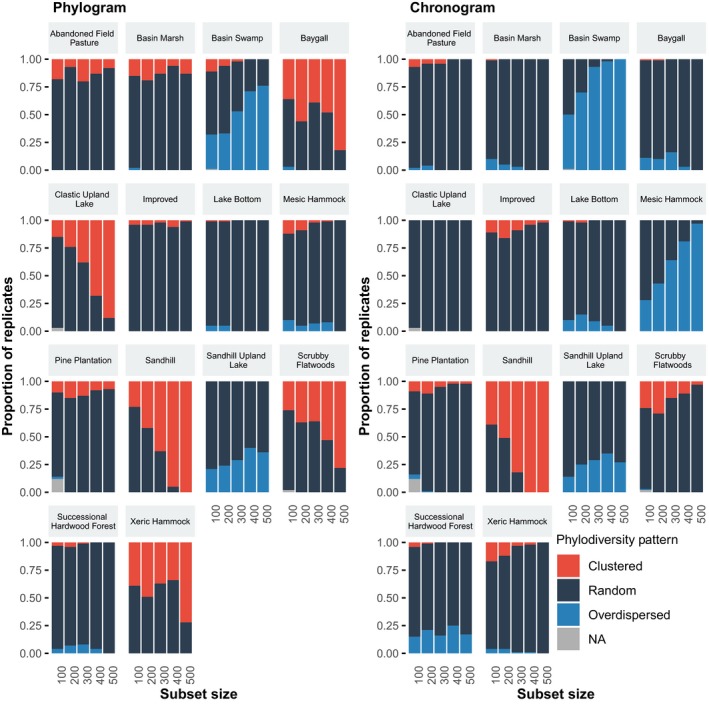
The effect of phylograms (left) versus chronograms (right) on the detection of patterns of phylodiversity (i.e., clustered, overdispersed, or random) across different dataset sizes. For each dataset size, phylograms were pruned from the complete phylogeny for 100 randomly sampled replicates, and chronograms were pruned from the time‐calibrated complete chronogram, and NRI indices were calculated for each community for each replicate and each type of phylogeny. For each type of phylogeny, community, and dataset size, the proportions of replicates with a clustered, overdispersed, and random pattern sum to one

In general, there were highly significant differences in the phylodiversity indices calculated based on phylograms versus chronograms (Tables S20–S25). Chronograms generally resulted in higher NRI, although phylograms showed higher NRI for several communities (Figure 7). For most communities, the two types of trees follow a parallel pattern of decreasing or increasing phylodiversity with increasing numbers of taxa in the subset. However, a number of communities (e.g., Xeric Hammock, Pine Plantation, Basin Marsh, Baygall) show diverging patterns, where the sign of the index differs between the chronograms and phylograms. Hence, for certain communities, the proportion of nonrandom phylogenetic patterns and the type of pattern differed widely between chronograms and phylograms (Figure 8). For the Xeric Hammock, Scrubby Flatwoods, Baygall, and Clastic Upland Lake communities, phylograms resulted in high proportions of significantly clustered patterns, whereas the corresponding chronograms showed random or overdispersed results; for the Mesic Hammock and Basin Swamp communities, the chronograms resulted in significantly overdispersed patterns while the phylograms showed either lower proportions of overdispersed patterns or random results. In general, chronograms resulted in phylogenetic patterns that were more overdispersed (i.e., had higher values) than the phylograms (Figure 7; Tables S22–S24).

## 4 DISCUSSION

We found that different tree reconstruction methods and sampling strategies have significant impacts on metrics and patterns of phylodiversity. Better understanding of the ways in which these methods may affect inferences drawn from these patterns (e.g., potentially influencing conservation decisions) will help inform the appropriate choice of methods in the future community phylogenetic studies.

### 4.1 Question 1

The number of taxa included in the regional dataset had a significant impact on the proportion of significant phylogenetic patterns that were observed in various communities, suggesting that studies that include higher proportions of the complete regional species pool are more likely to find significant phylogenetic patterns when they exist in the community. Depending on the question under investigation, this phenomenon could have a significant impact on the conclusions that are drawn. Studies that use only a small fraction of the overall species richness of a community may be unable to detect noteworthy phylogenetic patterns that exist in the community as a whole. Based on simulated data, Park et al. (2018) found that taxonomic undersampling can result in the underestimation of measures of phylodiversity, with communities that are phylogenetically clustered more likely to show these effects. Our findings, which show that higher species numbers in the regional phylogeny more often result in significant phylogenetic patterns (primarily clustering), also support this conclusion.

The increased likelihood of recovering significant phylogenetic patterns with increasing species richness is related to the power of these statistical tests. As described by Cadotte and Davies (2016, p. 51), it is expected that, due to decreasing variance with increasing species richness, NRI will increase with increased sampling. This means that communities with lower species richness would be less likely to show significant phylogenetic patterns due to the decreased statistical power of the test (Herrera, 2016). Therefore, researchers should use caution when using these methods to estimate the phylodiversity of species‐poor communities, as there may not be enough statistical power to detect nonrandom patterns even when they may exist in nature.

While our observation that phylodiversity (i.e., NRI) increases with species richness is not surprising, it is important to note that there is no point at which incomplete sampling equals complete taxonomic sampling. This finding, that NRI has a linear relationship with species richness, indicates that increased taxon sampling will always give a more accurate estimate of phylodiversity, and that limited taxon sampling cannot be guaranteed to ever represent the phylodiversity of the community as a whole. Our findings also suggest that the effects of reduced sampling on the detection of phylodiversity patterns may be quite variable, depending on the community being studied, and that researchers should aim to maximize the number of species that they sample to ensure that they are accurately representing the phylodiversity of the community. For indices such as PD_SES_, NRI, or NTI, we therefore recommend capturing as much of the species richness, and therefore phylodiversity, of a community as possible, to avoid potentially missing or misinterpreting phylogenetic patterns.

### 4.2 Question 2

Sampling decisions that are made for community phylogenetic studies can have an effect on the phylogenetic patterns observed, but studies are often not explicit about the reasons behind their taxon sampling scheme and how sampling decisions may impact results. Although studies would rarely sample taxa from the regional species pool in a truly random manner, there may be cases where elements of taxon selection may be partially or entirely random (e.g., Godoy et al., 2014; Kellar et al., 2015). Moreover, rather than trying to identify the optimal sampling method, this question addresses whether different taxon sampling approaches may affect the phylogenetic patterns that are observed, and in some cases, they can.

In some cases, we found that randomly sampling taxa for the regional phylogeny can result in either significantly higher or significantly lower effect sizes than sampling taxa proportional to family‐level species richness. However, there does not seem to be a consistent pattern for when these differences will be significant, based on the number of taxa either in the regional phylogeny or the community being studied. There is no consistent relationship between the likelihood of significant phylogenetic patterns within a community and whether the regional phylogeny is sampled randomly or proportionally to infrafamilial species richness.

We expect the impact of sampling strategies to vary depending on the community, because the relative diversity of species within a family is likely to vary widely among highly different communities. Some communities have fairly even species representation by family (e.g., Mesic Hammock or Clastic Upland Lake), while in others, the majority of species belong to one or a few highly diverse families (e.g., Ericaceae in Scrubby Flatwoods, Fagaceae in Xeric Hammock). Because targeted sampling is conducted on the regional dataset rather than on a community‐by‐community basis, taxa may not be sampled proportionally to their representation in individual communities; thus, the phylodiversity represented by this targeted sampling may more closely represent the phylodiversity of the larger region rather than an individual community. Moreover, targeted sampling essentially incorporates taxonomic information, and potentially bias, into the calculation of phylodiversity based on the arbitrary taxonomic rank of, in our case, family. Alternative sampling strategies can result in significantly different patterns depending on the community, such that decisions about sampling strategies should be made on a case‐by‐case basis and explicitly justified. The selected approach should be dictated by the question being asked.

### 4.3 Question 3

Phylogenetic patterns can vary widely when including only members of specific clades, such as the most diverse families at a site, in agreement with the findings of Ndiribe et al. (2013) and others who found that community phylogenetic structure can be lineage specific. Our study, which covers a broader range of communities and clades, represents further evidence that patterns can be lineage specific. Choosing the appropriate set of taxa for community phylogenetic studies may vary depending on the question, but is crucial (e.g., Cavender‐Bares et al., 2004; Cavender‐Bares, Keen, & Miles, 2006; Münkemüller et al., 2014). For example, in the Sandhill Upland Lake community, we found phylogenetic clustering for Fagaceae and Ericaeae, phylogenetic overdispersion for the complete dataset, and no significant phylogenetic pattern for the remaining five clades (Figure 5). With the exception of the complete dataset, each of these clades had similar species richness in this community. Thus, conclusions about the ecological processes taking place in the community would only be applicable to each clade and could not be generalized to the overall community (also suggested by Münkemüller et al., 2014).

We note that when targeting a specific clade, the patterns that are observed are phylodiversity patterns within that clade, not patterns of the clade within the entire community. For example, when Cavender‐Bares et al. (2004) studied patterns of phylodiversity of Floridian oaks, they identified patterns of overdispersion within Floridian oaks, not overdispersion of the overall community with more oaks present than expected from the regional species pool. To avoid biasing results or incorrectly interpreting phylogenetic patterns, it is important to ensure that the clade being studied is appropriate for the study question. Investigators hoping to answer questions about local processes affecting coexistence within a community may be misled if they only include individual clades rather than representatives from the overall diversity of the community.

Identifying matches or mismatches between the patterns that are found based on different clades or sets of taxa may highlight differences in the processes that have been involved in the assembly of the community as a whole. Traits of species may drive these differences in phylodiversity patterns among clades. For example, within a clade where functional traits are conserved, there may be more competitive interactions that reduce co‐occurrence, leading to phylogenetic overdispersion, while in another clade where certain functional traits are variable, competition may be reduced and coexistence may be facilitated, leading to phylogenetic clustering. Comparing the phylogenetic patterns observed in different clades may lead to unique insights into community assembly, and whether certain processes are clade specific or may apply to a functional group (e.g., trees) or the community as a whole. Future studies will further investigate the clade‐ and community‐specific phylodiversity patterns at the OSBS and will seek to measure trait diversity as well.

### 4.4 Question 4

When investigating the effect of using phylogenies that are pruned from a larger reconstructed phylogeny for a broader species pool versus those that are reconstructed directly for the taxa in the more limited regional species pool, we found that the difference between pruned and reconstructed phylogenies was not significant. Additionally, the proportions of results that show significantly nonrandom patterns are also similar for estimates based on pruned versus reconstructed phylogenies. We stress that these reconstructed and pruned phylogenies differed in the taxa included only at the time of phylogeny reconstruction, and that the final phylogenies from which the phylodiversity indices were calculated included the same taxa. This finding indicates that, in general, the differences in branch lengths that may be expected in phylogenies reconstructed based on different taxon sampling schemes do not have a significant effect on the detection of phylodiversity patterns. However, the range of potential differences in phylodiversity values for each replicate indicates that substantially different results could be obtained using a pruned versus reconstructed phylogeny, depending on the specific taxonomic composition of that tree. Based on their simulated and empirical data, Park et al. (2018) suggest that pruned phylogenies may be more reliable than reconstructed phylogenies, as the increased taxon sampling used in the more complete phylogeny to be pruned will result in better resolution and will improve the accuracy of the phylogeny. Erickson et al. (2014) also found that reconstructing a single, large phylogeny for multiple communities, rather than individual phylogenies for each community, improved the resolution of relationships in community phylogenies and resulted in more consistent estimates of phylodiversity. Therefore, using pruned phylogenies rather than phylogenies reconstructed for specific community phylogenetic studies has a number of potential advantages: community phylogenies may be pruned from phylogenies reconstructed using more complete and broader taxon sampling and which are, therefore, more well‐resolved and reliable; and when genetic data are unavailable, previously published phylogenies may be co‐opted for community phylogenetic studies, and supertrees, such as the Open Tree of Life (Hinchliff et al., 2015), may be used to represent relationships among distantly related organisms.

Using simulations and chronograms, Park et al. (2018) found that reconstructed phylogenies produce lower estimates of phylodiversity relative to pruned trees. In contrast, our results do not show a consistent pattern of shorter branch lengths in the reconstructed phylogenies. Instead, our results show that pruned and reconstructed phylogenies typically produce similar measures of phylodiversity and do not tend to alter the signal, and therefore the interpretation, of the phylogenetic pattern. In other words, our pruned and reconstructed phylogenies did not vary greatly, in part due to the specific tree reconstruction methods. These results may be limited to phylogenies reconstructed using few, slow‐evolving loci such as barcoding loci; future research could investigate how the number of loci and their rates of molecular evolution may impact the use of pruned versus reconstructed phylogenies. These results indicate that researchers could be able to use pruned and reconstructed phylogenies interchangeably, supporting findings from Cadotte, Cardinale, and Oakley (2008) and Cadotte et al. (2009). However, if researchers are concerned about potential differences in phylodiversity estimates from reconstructed and pruned phylogenies, we recommend, rather than individual trees, using a distribution of trees, such as by bootstrapping the taxon sampling process. We recommend that researchers use the best possible tree available and note that increased taxon sampling tends to improve the accuracy of phylogeny reconstruction (e.g., Linder, Hardy, & Rutschmann, 2005; Park et al., 2018). Although we did not test the effects of using taxonomy‐based trees that lack reconstructed branch lengths (e.g., Phylomatic trees), the use of pruned phylogenies from sources such as the Open Tree of Life, combined with methods of adding branch lengths (such as time‐calibration or using GenBank sequence data, Allen et al., 2019; Smith & Brown, 2018), will likely facilitate many more studies, as newly reconstructed phylogenies will not be required.

### 4.5 Question 5

We found that using chronograms versus phylograms can have a large effect on estimates of phylodiversity and the detection of phylogenetic patterns, as also reported by Elliott et al. (2018) and Allen et al. (2019). Our study complements this earlier work by demonstrating these patterns in a variety of communities and using different metrics. Chronograms tend to produce higher NRI than phylograms and may identify different phylogenetic patterns. During the process of making trees ultrametric (i.e., time‐calibrating), the trees are smoothed, where long branches are shortened and short branches are lengthened. This smoothing process may disproportionately affect branches deeper in the tree (i.e., internal branches representing older lineages), making short branches longer, and therefore inflating the total tree length. The effect of tree smoothing on phylodiversity is most pronounced with NRI, a metric that quantifies phylodiversity over the entire tree. NTI, a metric which quantifies the phylodiversity represented near the tips of the trees (i.e., terminal branches), shows this pattern to a lesser degree (see Supporting Information), indicating that this phenomenon is likely related to these branches deeper in the tree which contribute more to measures of NRI than NTI.

Chronograms measure the time since divergence and can be used to study the relative influence of biogeographic history and ecological processes on phylogenetic patterns of diversity (Elliott et al., 2018; Mishler et al., 2014), while phylograms measure the divergence in the characters used to reconstruct the phylogeny and can be used to compare phylodiversity with trait diversity within a community (Anderson, Shaw, & Olff, 2011; Elliott et al., 2018). As discussed by Elliott et al. (2018), phylograms are used under the assumption that changes in the characters used to reconstruct the tree are correlated with changes in the genes responsible for the traits affecting species coexistence and community assembly. The use of chronograms relies on this assumption as well, but has the added assumption of a molecular clock (or relaxed molecular clock; Elliott et al., 2018). The phylodiversity represented by chronograms and phylograms is related to changes in time or characters, respectively, so whether it is more appropriate to use a chronogram or a phylogram depends on the question being asked. Either both types of trees should be used and compared or the rationale for choosing one or the other tree representation should therefore be clearly stated, as this choice can alter the conclusions that are drawn.

## 5 CONCLUSIONS

Few studies have explicitly tested the impact of different tree reconstruction or taxon sampling methods on patterns of phylodiversity using an empirical dataset with multiple communities. This study examines five gaps in our understanding of how these methods may affect the detection and characterization of phylodiversity patterns. Although the specific patterns or trends that we observed in our dataset may not be generalizable to all community phylogenetic studies, our findings reinforce the idea that the methods that are used in community phylogenetic studies should be justified and explicitly stated, as these methods can often have a significant impact on the conclusions drawn. This is especially true for both the taxon sampling strategies and the tree reconstruction methods. Alternative taxon sampling strategies, whether random, targeted, or focused on specific clades, can result in different outcomes, so taxon sampling should be appropriate to the question being asked. Our study supports the use of pruned phylogenies as interchangeable with reconstructed phylogenies, with the caveat that there may be unpredictable instances where these trees give significantly different results. However, we found that greater care should be taken when choosing whether to use chronograms or phylograms, as these different tree representations can produce highly different results, with the chronograms more likely to show higher levels of phylodiversity and significant overdispersion rather than clustering. Ensuring that the methods are appropriate to the question is vital to correctly interpreting results of community phylogenetic studies.

## CONFLICT OF INTEREST

None declared.

## AUTHOR CONTRIBUTIONS

JJ, DS, and PS conceived the ideas and designed methodology; MW, LM, and KN collected the species from OSBS and obtained sequence data; JJ analyzed the data; JJ led the writing of the manuscript. All authors contributed critically to the drafts and gave final approval for publication.

## Figures and Tables

**Figure C1:**
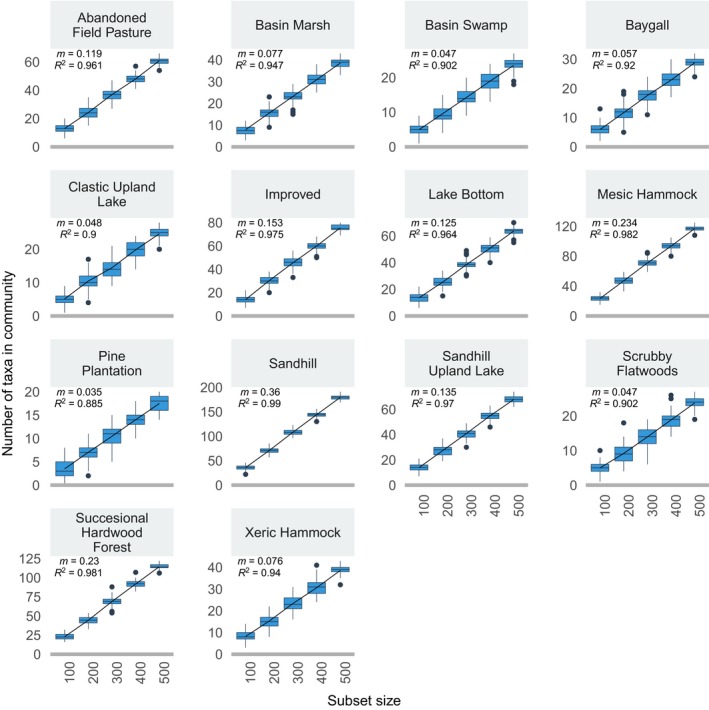
Correlation of the number of taxa in each community and number of taxa in the regional dataset for randomly sampled subsets for 14 communities

**Figure C2:**
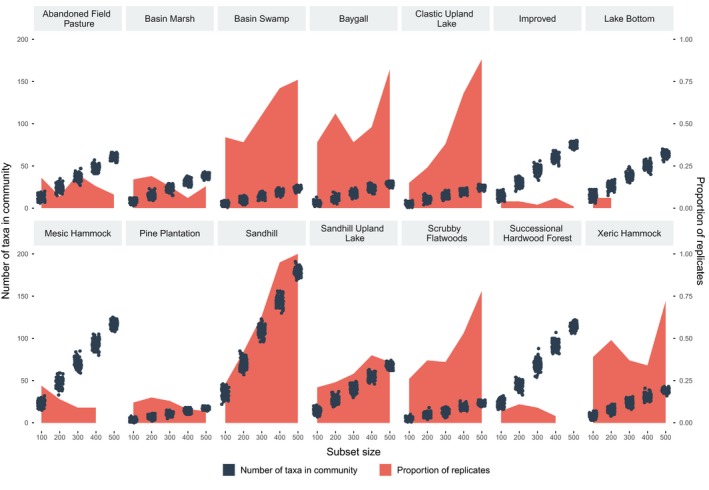
Number of taxa in a community plotted on the left *y*‐axis against the number of taxa in a subset, with the proportion of replicates showing a significant phylogenetic pattern plotted on the right *y*‐axis

## Data Availability

The alignment matrix, phylogenies, and community data are deposited on Dryad (doi:10.5061/dryad.5m9n159). Barcoding loci have been archived on GenBank (Table S26). Scripts are available at https://github.com/jjantzen/CommPhylogeneticsOSBS.

## APPENDIX A

**Table A1:** Primers for PCR and sequencing of barcoding DNA loci with references

Locus	Primer	Primer sequence	Reference
rbcL	a‐F	ATGTCACCACAAACAGAGACTAAAGC	Levin et al. (2003)
rbcL	a‐R	GTAAAATCAAGTCCACCRCG	Kress et al. (2009)
matK	xf	TAATTTACGATCAATTCATTC	Ford et al. (2009)
matK	MALP	ACAAGAAAGTCGAAGTAT	Dunning and Savolainen (2010)
matK	1R_Kim	ACCCAGTCCATCTGGAAATCTTGGTTC	CBOL Plant Working Group (2009)
matK	3F_Kim	CGTACAGTACTTTTGTGTTTACGAG	CBOL Plant Working Group (2009)
matK	472F	CCCRTYCATCTGGAAATCTTGGTTC	Yu et al. (2011)
matK	1248R	GCTRTRATAATGAGAAAGATTTCTGC	Yu et al. (2011)

## APPENDIX B

**Table B1:** Calibration Points from (Bell et al., 2010) for time‐calibrating the maximum‐likelihood phylogeny for the complete dataset of 572 vascular plant species from the Ordway‐Swisher Biological Station. The node (clade) to be calibrated is given with two internal specifiers and the minimum and maximum ages

Clade	Internal specifier 1	Internal specifier 2	Min age (my)	Max age (my)
Asteraceae	Asteraceae_Cirsium_horridulum	Asteraceae_Liatris_gracilis	47.69	53.83
Ericales	Ericaceae_Vaccinium_arboreum	Primulaceae_Ardisia_crenata	107.44	117.07
Eudicots	Ranunculaceae_Clematis_reticulata	Asteraceae_Liatris_gracilis	133	135.6
Fabales	Polygalaceae_Asemeia_violacea	Fabaceae_Desmodium_floridanum	108.59	117.35
Magnoliales	Magnoliaceae_Magnolia_grandiflora	Annonaceae_Asimina_incana	121.8	131.77
Monilophytes	Psilotaceae_Psilotum_nudum	Osmundaceae_Osmunda_regalis	354	354
Monocots	Araceae_Lemna_valdiviana	Poaceae_Dichanthelium_commutatum	134.74	136.72
Poales	Bromeliaceae_Tillandsia_usneoides	Poaceae_Dichanthelium_commutatum	99.77	116.38
Root	Lycopodiaceae_Lycopodiella_appressa	Asteraceae_Liatris_gracilis	–	454

## APPENDIX C

To confirm that an increase in taxon sampling at the regional species pool level resulted in increased taxon sampling in each community, we plotted the correlation between taxa in the regional species pool and in each community (Figure C1). The increase in the number of taxa in the regional phylogeny was highly correlated with an increase in number of taxa in a given community, although the rate of increase varied widely between communities. The number of taxa in a community increases with increasing number of taxa in the subset, but this increase in species richness does not consistently correspond with an increase in the proportion of replicates showing a significant phylogenetic pattern (Figure C2).
