# Supplementary material for: Effects of taxon sampling and tree reconstruction methods on phylodiversity metrics
Source: Ecol Evol. 2019 Aug 21;9(17):9479–99. doi: 10.1002/ece3.5425 (PMC6745870; doi:10.1002/ece3.5425)
Supplement: Supplementary file 1 [file ECE3-9-9479-s001.docx]

**Supporting Information**

**Appendix IV**

Summary of statistical tests, data that were plotted and additional data which support the findings.

Table S1: Results of Tukey Test comparing the values of NRI for five randomly sampled subset sizes for 14 communities (Question 1).

| Subsets compared | Difference | Lower 95% confidence interval | Upper 95% confidence interval | Adjusted p-value | Community |
| --- | --- | --- | --- | --- | --- |
| 200-100 | -0.15625 | -0.47787 | 0.165365 | 0.67252 | Basin.Marsh |
| 300-100 | -0.03845 | -0.36007 | 0.283163 | 0.997516 | Basin.Marsh |
| 400-100 | -0.02625 | -0.34786 | 0.29537 | 0.999446 | Basin.Marsh |
| 500-100 | -0.30704 | -0.62865 | 0.014577 | 0.069393 | Basin.Marsh |
| 300-200 | 0.117798 | -0.20382 | 0.439413 | 0.854065 | Basin.Marsh |
| 400-200 | 0.130004 | -0.19161 | 0.45162 | 0.803015 | Basin.Marsh |
| 500-200 | -0.15079 | -0.4724 | 0.170827 | 0.701448 | Basin.Marsh |
| 400-300 | 0.012207 | -0.30941 | 0.333822 | 0.999974 | Basin.Marsh |
| 500-300 | -0.26859 | -0.5902 | 0.053029 | 0.150966 | Basin.Marsh |
| 500-400 | -0.28079 | -0.60241 | 0.040823 | 0.119618 | Basin.Marsh |
| 200-100 | 0.227307 | -0.25604 | 0.710651 | 0.699033 | Basin.Swamp |
| 300-100 | 0.934801 | 0.451456 | 1.418146 | 1.78E-06 | Basin.Swamp |
| 400-100 | 1.326353 | 0.843008 | 1.809697 | 1.09E-10 | Basin.Swamp |
| 500-100 | 1.313083 | 0.829738 | 1.796428 | 1.10E-10 | Basin.Swamp |
| 300-200 | 0.707494 | 0.225366 | 1.189623 | 0.000647 | Basin.Swamp |
| 400-200 | 1.099046 | 0.616917 | 1.581175 | 9.45E-09 | Basin.Swamp |
| 500-200 | 1.085777 | 0.603648 | 1.567905 | 1.47E-08 | Basin.Swamp |
| 400-300 | 0.391552 | -0.09058 | 0.87368 | 0.172696 | Basin.Swamp |
| 500-300 | 0.378282 | -0.10385 | 0.860411 | 0.201558 | Basin.Swamp |
| 500-400 | -0.01327 | -0.4954 | 0.468859 | 0.999993 | Basin.Swamp |
| 200-100 | -0.45585 | -0.84402 | -0.06768 | 0.012062 | Baygall |
| 300-100 | -0.38389 | -0.77206 | 0.004282 | 0.05424 | Baygall |
| 400-100 | -0.81358 | -1.20175 | -0.42541 | 1.66E-07 | Baygall |
| 500-100 | -0.95946 | -1.34763 | -0.57129 | 4.82E-10 | Baygall |
| 300-200 | 0.071959 | -0.31621 | 0.46013 | 0.986612 | Baygall |
| 400-200 | -0.35773 | -0.7459 | 0.030441 | 0.087163 | Baygall |
| 500-200 | -0.50361 | -0.89179 | -0.11544 | 0.00382 | Baygall |
| 400-300 | -0.42969 | -0.81786 | -0.04152 | 0.021527 | Baygall |
| 500-300 | -0.57557 | -0.96374 | -0.1874 | 0.000545 | Baygall |
| 500-400 | -0.14588 | -0.53405 | 0.242288 | 0.841919 | Baygall |
| 200-100 | 0.049243 | -0.21011 | 0.308594 | 0.985352 | Lake.Bottom |
| 300-100 | 0.060279 | -0.19907 | 0.319629 | 0.96908 | Lake.Bottom |
| 400-100 | 0.105125 | -0.15423 | 0.364475 | 0.801406 | Lake.Bottom |
| 500-100 | 0.194105 | -0.06525 | 0.453455 | 0.244291 | Lake.Bottom |
| 300-200 | 0.011036 | -0.24831 | 0.270386 | 0.999958 | Lake.Bottom |
| 400-200 | 0.055881 | -0.20347 | 0.315232 | 0.976561 | Lake.Bottom |
| 500-200 | 0.144862 | -0.11449 | 0.404212 | 0.543891 | Lake.Bottom |
| 400-300 | 0.044846 | -0.2145 | 0.304196 | 0.989708 | Lake.Bottom |
| 500-300 | 0.133826 | -0.12552 | 0.393177 | 0.61972 | Lake.Bottom |
| 500-400 | 0.08898 | -0.17037 | 0.348331 | 0.881431 | Lake.Bottom |
| 200-100 | 0.105571 | -0.26285 | 0.473995 | 0.935053 | Mesic.Hammock |
| 300-100 | 0.559991 | 0.191568 | 0.928415 | 0.000358 | Mesic.Hammock |
| 400-100 | 0.641659 | 0.273235 | 1.010082 | 2.41E-05 | Mesic.Hammock |
| 500-100 | 0.630355 | 0.261931 | 0.998779 | 3.57E-05 | Mesic.Hammock |
| 300-200 | 0.45442 | 0.085997 | 0.822844 | 0.007048 | Mesic.Hammock |
| 400-200 | 0.536088 | 0.167664 | 0.904511 | 0.000741 | Mesic.Hammock |
| 500-200 | 0.524784 | 0.15636 | 0.893208 | 0.001034 | Mesic.Hammock |
| 400-300 | 0.081667 | -0.28676 | 0.450091 | 0.973987 | Mesic.Hammock |
| 500-300 | 0.070363 | -0.29806 | 0.438787 | 0.985027 | Mesic.Hammock |
| 500-400 | -0.0113 | -0.37973 | 0.35712 | 0.999989 | Mesic.Hammock |
| 200-100 | -0.21203 | -0.44995 | 0.025888 | 0.10653 | Pine.Plantation |
| 300-100 | -0.30105 | -0.53897 | -0.06314 | 0.005215 | Pine.Plantation |
| 400-100 | -0.43547 | -0.67339 | -0.19755 | 7.51E-06 | Pine.Plantation |
| 500-100 | -0.52544 | -0.76335 | -0.28752 | 2.96E-08 | Pine.Plantation |
| 300-200 | -0.08902 | -0.31922 | 0.141176 | 0.827415 | Pine.Plantation |
| 400-200 | -0.22344 | -0.45364 | 0.006763 | 0.06196 | Pine.Plantation |
| 500-200 | -0.31341 | -0.54361 | -0.08321 | 0.002008 | Pine.Plantation |
| 400-300 | -0.13441 | -0.36461 | 0.095787 | 0.498872 | Pine.Plantation |
| 500-300 | -0.22438 | -0.45458 | 0.005818 | 0.060157 | Pine.Plantation |
| 500-400 | -0.08997 | -0.32017 | 0.140231 | 0.821813 | Pine.Plantation |
| 200-100 | -0.4073 | -0.62564 | -0.18895 | 4.63E-06 | Sandhill |
| 300-100 | -0.90785 | -1.1262 | -0.68951 | 1.10E-10 | Sandhill |
| 400-100 | -1.23616 | -1.4545 | -1.01781 | 1.10E-10 | Sandhill |
| 500-100 | -1.46207 | -1.68041 | -1.24372 | 1.10E-10 | Sandhill |
| 300-200 | -0.50055 | -0.7189 | -0.28221 | 7.67E-09 | Sandhill |
| 400-200 | -0.82886 | -1.04721 | -0.61051 | 1.10E-10 | Sandhill |
| 500-200 | -1.05477 | -1.27311 | -0.83642 | 1.10E-10 | Sandhill |
| 400-300 | -0.3283 | -0.54665 | -0.10996 | 0.000432 | Sandhill |
| 500-300 | -0.55421 | -0.77256 | -0.33587 | 2.26E-10 | Sandhill |
| 500-400 | -0.22591 | -0.44425 | -0.00756 | 0.038461 | Sandhill |
| 200-100 | 0.216789 | -0.11799 | 0.551568 | 0.390613 | Sandhill.Upland.Lake |
| 300-100 | 0.532741 | 0.197962 | 0.86752 | 0.000156 | Sandhill.Upland.Lake |
| 400-100 | 0.716569 | 0.38179 | 1.051349 | 8.44E-08 | Sandhill.Upland.Lake |
| 500-100 | 0.726684 | 0.391905 | 1.061464 | 5.29E-08 | Sandhill.Upland.Lake |
| 300-200 | 0.315952 | -0.01883 | 0.650731 | 0.074891 | Sandhill.Upland.Lake |
| 400-200 | 0.49978 | 0.165001 | 0.83456 | 0.000487 | Sandhill.Upland.Lake |
| 500-200 | 0.509895 | 0.175116 | 0.844674 | 0.000346 | Sandhill.Upland.Lake |
| 400-300 | 0.183828 | -0.15095 | 0.518608 | 0.560759 | Sandhill.Upland.Lake |
| 500-300 | 0.193943 | -0.14084 | 0.528722 | 0.507024 | Sandhill.Upland.Lake |
| 500-400 | 0.010115 | -0.32466 | 0.344894 | 0.999989 | Sandhill.Upland.Lake |
| 200-100 | 0.103334 | -0.21565 | 0.422316 | 0.901695 | Succesional.Hardwood.Forest |
| 300-100 | 0.262979 | -0.056 | 0.581961 | 0.160801 | Succesional.Hardwood.Forest |
| 400-100 | 0.518175 | 0.199194 | 0.837157 | 0.000105 | Succesional.Hardwood.Forest |
| 500-100 | 0.414045 | 0.095063 | 0.733026 | 0.003797 | Succesional.Hardwood.Forest |
| 300-200 | 0.159645 | -0.15934 | 0.478626 | 0.647058 | Succesional.Hardwood.Forest |
| 400-200 | 0.414841 | 0.095859 | 0.733822 | 0.003705 | Succesional.Hardwood.Forest |
| 500-200 | 0.31071 | -0.00827 | 0.629692 | 0.060444 | Succesional.Hardwood.Forest |
| 400-300 | 0.255196 | -0.06379 | 0.574177 | 0.184999 | Succesional.Hardwood.Forest |
| 500-300 | 0.151065 | -0.16792 | 0.470047 | 0.693447 | Succesional.Hardwood.Forest |
| 500-400 | -0.10413 | -0.42311 | 0.214851 | 0.899174 | Succesional.Hardwood.Forest |
| 200-100 | -0.31112 | -0.66766 | 0.045413 | 0.119953 | Xeric.Hammock |
| 300-100 | -0.42801 | -0.78455 | -0.07148 | 0.009543 | Xeric.Hammock |
| 400-100 | -0.46473 | -0.82127 | -0.1082 | 0.003599 | Xeric.Hammock |
| 500-100 | -0.94521 | -1.30174 | -0.58867 | 1.26E-10 | Xeric.Hammock |
| 300-200 | -0.11689 | -0.47343 | 0.239645 | 0.897741 | Xeric.Hammock |
| 400-200 | -0.15361 | -0.51014 | 0.202926 | 0.76312 | Xeric.Hammock |
| 500-200 | -0.63409 | -0.99062 | -0.27755 | 1.49E-05 | Xeric.Hammock |
| 400-300 | -0.03672 | -0.39325 | 0.319817 | 0.998615 | Xeric.Hammock |
| 500-300 | -0.5172 | -0.87373 | -0.16066 | 0.000778 | Xeric.Hammock |
| 500-400 | -0.48048 | -0.83701 | -0.12394 | 0.002311 | Xeric.Hammock |
| 200-100 | -0.17867 | -0.3227 | -0.03463 | 0.006599 | Improved |
| 300-100 | -0.4283 | -0.57233 | -0.28426 | 1.10E-10 | Improved |
| 400-100 | -0.55587 | -0.6999 | -0.41183 | 1.10E-10 | Improved |
| 500-100 | -0.64334 | -0.78738 | -0.49931 | 1.10E-10 | Improved |
| 300-200 | -0.24963 | -0.39366 | -0.10559 | 2.69E-05 | Improved |
| 400-200 | -0.3772 | -0.52124 | -0.23316 | 1.38E-10 | Improved |
| 500-200 | -0.46468 | -0.60871 | -0.32064 | 1.10E-10 | Improved |
| 400-300 | -0.12757 | -0.27161 | 0.016465 | 0.110388 | Improved |
| 500-300 | -0.21505 | -0.35908 | -0.07101 | 0.000486 | Improved |
| 500-400 | -0.08748 | -0.23151 | 0.05656 | 0.458107 | Improved |
| 200-100 | 0.160262 | -0.08615 | 0.406679 | 0.386057 | Abandoned.Field.Pasture |
| 300-100 | -0.2361 | -0.48252 | 0.010314 | 0.067717 | Abandoned.Field.Pasture |
| 400-100 | -0.2713 | -0.51771 | -0.02488 | 0.022625 | Abandoned.Field.Pasture |
| 500-100 | -0.37996 | -0.62637 | -0.13354 | 0.000278 | Abandoned.Field.Pasture |
| 300-200 | -0.39636 | -0.64278 | -0.14995 | 0.000127 | Abandoned.Field.Pasture |
| 400-200 | -0.43156 | -0.67798 | -0.18514 | 2.12E-05 | Abandoned.Field.Pasture |
| 500-200 | -0.54022 | -0.78664 | -0.2938 | 3.77E-08 | Abandoned.Field.Pasture |
| 400-300 | -0.03519 | -0.28161 | 0.211223 | 0.995049 | Abandoned.Field.Pasture |
| 500-300 | -0.14385 | -0.39027 | 0.102563 | 0.499145 | Abandoned.Field.Pasture |
| 500-400 | -0.10866 | -0.35508 | 0.137757 | 0.747183 | Abandoned.Field.Pasture |
| 200-100 | -0.31483 | -0.55048 | -0.07918 | 0.002601 | Scrubby.Flatwoods |
| 300-100 | -0.50996 | -0.74561 | -0.27431 | 5.87E-08 | Scrubby.Flatwoods |
| 400-100 | -0.70618 | -0.94183 | -0.47053 | 1.01E-10 | Scrubby.Flatwoods |
| 500-100 | -0.89157 | -1.12723 | -0.65592 | 1.01E-10 | Scrubby.Flatwoods |
| 300-200 | -0.19513 | -0.42959 | 0.03933 | 0.153553 | Scrubby.Flatwoods |
| 400-200 | -0.39135 | -0.62581 | -0.15689 | 6.04E-05 | Scrubby.Flatwoods |
| 500-200 | -0.57675 | -0.8112 | -0.34229 | 5.59E-10 | Scrubby.Flatwoods |
| 400-300 | -0.19622 | -0.43068 | 0.038237 | 0.149353 | Scrubby.Flatwoods |
| 500-300 | -0.38162 | -0.61608 | -0.14716 | 0.000101 | Scrubby.Flatwoods |
| 500-400 | -0.1854 | -0.41985 | 0.049064 | 0.194856 | Scrubby.Flatwoods |
| 200-100 | -0.26916 | -0.42495 | -0.11337 | 2.88E-05 | Clastic.Upland.Lake |
| 300-100 | -0.44397 | -0.59976 | -0.28818 | 9.76E-11 | Clastic.Upland.Lake |
| 400-100 | -0.66924 | -0.82503 | -0.51345 | 9.72E-11 | Clastic.Upland.Lake |
| 500-100 | -0.82918 | -0.98497 | -0.67339 | 9.72E-11 | Clastic.Upland.Lake |
| 300-200 | -0.17481 | -0.32941 | -0.02021 | 0.017621 | Clastic.Upland.Lake |
| 400-200 | -0.40008 | -0.55468 | -0.24548 | 1.46E-10 | Clastic.Upland.Lake |
| 500-200 | -0.56002 | -0.71462 | -0.40542 | 9.73E-11 | Clastic.Upland.Lake |
| 400-300 | -0.22527 | -0.37987 | -0.07067 | 0.000725 | Clastic.Upland.Lake |
| 500-300 | -0.3852 | -0.5398 | -0.2306 | 3.62E-10 | Clastic.Upland.Lake |
| 500-400 | -0.15993 | -0.31453 | -0.00534 | 0.038498 | Clastic.Upland.Lake |

Table S2: Results of Tukey Test comparing the values of NTI for five randomly sampled subset sizes for 14 communities (Question 1).

| Subsets compared | Difference | Lower 95% confidence interval | Upper 95% confidence interval | Adjusted p-value | Community |
| --- | --- | --- | --- | --- | --- |
| 200-100 | 0.093465 | -0.24958 | 0.436511 | 0.945488 | Basin.Marsh |
| 300-100 | 0.317508 | -0.02554 | 0.660553 | 0.084852 | Basin.Marsh |
| 400-100 | 0.516704 | 0.173658 | 0.859749 | 0.000419 | Basin.Marsh |
| 500-100 | 0.29975 | -0.0433 | 0.642795 | 0.119076 | Basin.Marsh |
| 300-200 | 0.224042 | -0.119 | 0.567088 | 0.381658 | Basin.Marsh |
| 400-200 | 0.423238 | 0.080193 | 0.766284 | 0.007025 | Basin.Marsh |
| 500-200 | 0.206285 | -0.13676 | 0.54933 | 0.468459 | Basin.Marsh |
| 400-300 | 0.199196 | -0.14385 | 0.542242 | 0.504635 | Basin.Marsh |
| 500-300 | -0.01776 | -0.3608 | 0.325288 | 0.999909 | Basin.Marsh |
| 500-400 | -0.21695 | -0.56 | 0.126092 | 0.415509 | Basin.Marsh |
| 200-100 | 0.010896 | -0.32075 | 0.342541 | 0.999985 | Basin.Swamp |
| 300-100 | -0.41832 | -0.74996 | -0.08667 | 0.005416 | Basin.Swamp |
| 400-100 | -0.61458 | -0.94622 | -0.28293 | 5.48E-06 | Basin.Swamp |
| 500-100 | -0.83815 | -1.16979 | -0.5065 | 2.47E-10 | Basin.Swamp |
| 300-200 | -0.42921 | -0.76002 | -0.0984 | 0.003818 | Basin.Swamp |
| 400-200 | -0.62548 | -0.95629 | -0.29466 | 3.27E-06 | Basin.Swamp |
| 500-200 | -0.84904 | -1.17985 | -0.51823 | 1.76E-10 | Basin.Swamp |
| 400-300 | -0.19626 | -0.52707 | 0.134548 | 0.48248 | Basin.Swamp |
| 500-300 | -0.41983 | -0.75064 | -0.08902 | 0.005028 | Basin.Swamp |
| 500-400 | -0.22357 | -0.55438 | 0.107243 | 0.345924 | Basin.Swamp |
| 200-100 | -0.01718 | -0.37111 | 0.33675 | 0.99993 | Baygall |
| 300-100 | -0.05289 | -0.40683 | 0.301036 | 0.994104 | Baygall |
| 400-100 | -0.4227 | -0.77663 | -0.06877 | 0.010091 | Baygall |
| 500-100 | -0.72253 | -1.07646 | -0.3686 | 3.76E-07 | Baygall |
| 300-200 | -0.03571 | -0.38965 | 0.318218 | 0.998721 | Baygall |
| 400-200 | -0.40552 | -0.75945 | -0.05159 | 0.015492 | Baygall |
| 500-200 | -0.70535 | -1.05928 | -0.35142 | 7.66E-07 | Baygall |
| 400-300 | -0.36981 | -0.72374 | -0.01587 | 0.035528 | Baygall |
| 500-300 | -0.66963 | -1.02356 | -0.3157 | 3.21E-06 | Baygall |
| 500-400 | -0.29983 | -0.65376 | 0.054103 | 0.140439 | Baygall |
| 200-100 | -0.41998 | -0.73535 | -0.10462 | 0.002715 | Lake.Bottom |
| 300-100 | -0.95228 | -1.26764 | -0.63692 | 1.10E-10 | Lake.Bottom |
| 400-100 | -1.36918 | -1.68455 | -1.05382 | 1.10E-10 | Lake.Bottom |
| 500-100 | -1.81927 | -2.13463 | -1.50391 | 1.10E-10 | Lake.Bottom |
| 300-200 | -0.5323 | -0.84766 | -0.21694 | 4.78E-05 | Lake.Bottom |
| 400-200 | -0.9492 | -1.26456 | -0.63384 | 1.10E-10 | Lake.Bottom |
| 500-200 | -1.39929 | -1.71465 | -1.08392 | 1.10E-10 | Lake.Bottom |
| 400-300 | -0.4169 | -0.73226 | -0.10154 | 0.002994 | Lake.Bottom |
| 500-300 | -0.86699 | -1.18235 | -0.55162 | 1.13E-10 | Lake.Bottom |
| 500-400 | -0.45009 | -0.76545 | -0.13472 | 0.001004 | Lake.Bottom |
| 200-100 | -0.50504 | -0.79228 | -0.21779 | 1.94E-05 | Mesic.Hammock |
| 300-100 | -1.04667 | -1.33392 | -0.75943 | 1.10E-10 | Mesic.Hammock |
| 400-100 | -1.24651 | -1.53376 | -0.95927 | 1.10E-10 | Mesic.Hammock |
| 500-100 | -1.4299 | -1.71715 | -1.14266 | 1.10E-10 | Mesic.Hammock |
| 300-200 | -0.54164 | -0.82888 | -0.25439 | 3.50E-06 | Mesic.Hammock |
| 400-200 | -0.74148 | -1.02872 | -0.45423 | 1.64E-10 | Mesic.Hammock |
| 500-200 | -0.92487 | -1.21211 | -0.63762 | 1.10E-10 | Mesic.Hammock |
| 400-300 | -0.19984 | -0.48709 | 0.087405 | 0.316085 | Mesic.Hammock |
| 500-300 | -0.38323 | -0.67048 | -0.09598 | 0.002649 | Mesic.Hammock |
| 500-400 | -0.18339 | -0.47064 | 0.103855 | 0.405541 | Mesic.Hammock |
| 200-100 | -0.22865 | -0.52345 | 0.066159 | 0.211669 | Pine.Plantation |
| 300-100 | -0.73732 | -1.03213 | -0.44251 | 2.91E-10 | Pine.Plantation |
| 400-100 | -1.08199 | -1.37679 | -0.78718 | 6.25E-11 | Pine.Plantation |
| 500-100 | -1.44041 | -1.73521 | -1.1456 | 6.24E-11 | Pine.Plantation |
| 300-200 | -0.50867 | -0.79391 | -0.22343 | 1.40E-05 | Pine.Plantation |
| 400-200 | -0.85334 | -1.13858 | -0.5681 | 6.25E-11 | Pine.Plantation |
| 500-200 | -1.21176 | -1.497 | -0.92652 | 6.24E-11 | Pine.Plantation |
| 400-300 | -0.34467 | -0.62991 | -0.05943 | 0.008889 | Pine.Plantation |
| 500-300 | -0.70309 | -0.98833 | -0.41784 | 4.90E-10 | Pine.Plantation |
| 500-400 | -0.35842 | -0.64366 | -0.07318 | 0.005673 | Pine.Plantation |
| 200-100 | -0.85719 | -1.16565 | -0.54873 | 1.12E-10 | Sandhill |
| 300-100 | -1.3797 | -1.68816 | -1.07124 | 1.10E-10 | Sandhill |
| 400-100 | -1.7705 | -2.07896 | -1.46204 | 1.10E-10 | Sandhill |
| 500-100 | -1.96814 | -2.2766 | -1.65968 | 1.10E-10 | Sandhill |
| 300-200 | -0.52251 | -0.83097 | -0.21405 | 4.43E-05 | Sandhill |
| 400-200 | -0.91331 | -1.22177 | -0.60485 | 1.10E-10 | Sandhill |
| 500-200 | -1.11095 | -1.41941 | -0.80249 | 1.10E-10 | Sandhill |
| 400-300 | -0.3908 | -0.69926 | -0.08234 | 0.005132 | Sandhill |
| 500-300 | -0.58844 | -0.8969 | -0.27998 | 2.58E-06 | Sandhill |
| 500-400 | -0.19764 | -0.5061 | 0.110824 | 0.401795 | Sandhill |
| 200-100 | -0.23923 | -0.59482 | 0.116359 | 0.350625 | Sandhill.Upland.Lake |
| 300-100 | -1.32956 | -1.68515 | -0.97397 | 1.10E-10 | Sandhill.Upland.Lake |
| 400-100 | -2.28628 | -2.64186 | -1.93069 | 1.10E-10 | Sandhill.Upland.Lake |
| 500-100 | -3.21559 | -3.57117 | -2.86 | 1.10E-10 | Sandhill.Upland.Lake |
| 300-200 | -1.09033 | -1.44592 | -0.73474 | 1.10E-10 | Sandhill.Upland.Lake |
| 400-200 | -2.04705 | -2.40264 | -1.69146 | 1.10E-10 | Sandhill.Upland.Lake |
| 500-200 | -2.97636 | -3.33195 | -2.62077 | 1.10E-10 | Sandhill.Upland.Lake |
| 400-300 | -0.95672 | -1.3123 | -0.60113 | 1.18E-10 | Sandhill.Upland.Lake |
| 500-300 | -1.88603 | -2.24161 | -1.53044 | 1.10E-10 | Sandhill.Upland.Lake |
| 500-400 | -0.92931 | -1.2849 | -0.57372 | 1.41E-10 | Sandhill.Upland.Lake |
| 200-100 | 0.01759 | -0.28772 | 0.322896 | 0.999861 | Succesional.Hardwood.Forest |
| 300-100 | -0.02728 | -0.33259 | 0.278022 | 0.999207 | Succesional.Hardwood.Forest |
| 400-100 | 0.025555 | -0.27975 | 0.330861 | 0.999388 | Succesional.Hardwood.Forest |
| 500-100 | -0.06336 | -0.36866 | 0.241951 | 0.979599 | Succesional.Hardwood.Forest |
| 300-200 | -0.04487 | -0.35018 | 0.260432 | 0.99447 | Succesional.Hardwood.Forest |
| 400-200 | 0.007965 | -0.29734 | 0.313271 | 0.999994 | Succesional.Hardwood.Forest |
| 500-200 | -0.08095 | -0.38625 | 0.224361 | 0.950464 | Succesional.Hardwood.Forest |
| 400-300 | 0.052839 | -0.25247 | 0.358145 | 0.989673 | Succesional.Hardwood.Forest |
| 500-300 | -0.03607 | -0.34138 | 0.269235 | 0.997629 | Succesional.Hardwood.Forest |
| 500-400 | -0.08891 | -0.39422 | 0.216396 | 0.93134 | Succesional.Hardwood.Forest |
| 200-100 | -0.45118 | -0.80382 | -0.09853 | 0.004552 | Xeric.Hammock |
| 300-100 | -0.56565 | -0.91829 | -0.213 | 0.000134 | Xeric.Hammock |
| 400-100 | -0.76229 | -1.11493 | -0.40964 | 6.08E-08 | Xeric.Hammock |
| 500-100 | -1.29724 | -1.64989 | -0.9446 | 1.10E-10 | Xeric.Hammock |
| 300-200 | -0.11447 | -0.46711 | 0.238172 | 0.901036 | Xeric.Hammock |
| 400-200 | -0.31111 | -0.66375 | 0.041532 | 0.112865 | Xeric.Hammock |
| 500-200 | -0.84607 | -1.19871 | -0.49342 | 1.40E-09 | Xeric.Hammock |
| 400-300 | -0.19664 | -0.54928 | 0.156002 | 0.545561 | Xeric.Hammock |
| 500-300 | -0.7316 | -1.08424 | -0.37895 | 2.29E-07 | Xeric.Hammock |
| 500-400 | -0.53496 | -0.8876 | -0.18231 | 0.000371 | Xeric.Hammock |
| 200-100 | -0.39802 | -0.60874 | -0.18731 | 3.35E-06 | Improved |
| 300-100 | -0.87092 | -1.08163 | -0.6602 | 1.10E-10 | Improved |
| 400-100 | -1.16047 | -1.37118 | -0.94975 | 1.10E-10 | Improved |
| 500-100 | -1.29691 | -1.50763 | -1.0862 | 1.10E-10 | Improved |
| 300-200 | -0.4729 | -0.68361 | -0.26218 | 1.66E-08 | Improved |
| 400-200 | -0.76245 | -0.97316 | -0.55173 | 1.10E-10 | Improved |
| 500-200 | -0.89889 | -1.1096 | -0.68817 | 1.10E-10 | Improved |
| 400-300 | -0.28955 | -0.50027 | -0.07883 | 0.001759 | Improved |
| 500-300 | -0.42599 | -0.63671 | -0.21528 | 5.03E-07 | Improved |
| 500-400 | -0.13644 | -0.34716 | 0.074272 | 0.390669 | Improved |
| 200-100 | 0.091067 | -0.15506 | 0.337191 | 0.84937 | Abandoned.Field.Pasture |
| 300-100 | -0.0841 | -0.33023 | 0.162019 | 0.882956 | Abandoned.Field.Pasture |
| 400-100 | -0.07616 | -0.32228 | 0.169966 | 0.915631 | Abandoned.Field.Pasture |
| 500-100 | -0.19203 | -0.43815 | 0.054096 | 0.206466 | Abandoned.Field.Pasture |
| 300-200 | -0.17517 | -0.42129 | 0.070952 | 0.293124 | Abandoned.Field.Pasture |
| 400-200 | -0.16722 | -0.41335 | 0.078898 | 0.340398 | Abandoned.Field.Pasture |
| 500-200 | -0.28309 | -0.52922 | -0.03697 | 0.014905 | Abandoned.Field.Pasture |
| 400-300 | 0.007947 | -0.23818 | 0.25407 | 0.999986 | Abandoned.Field.Pasture |
| 500-300 | -0.10792 | -0.35405 | 0.1382 | 0.751104 | Abandoned.Field.Pasture |
| 500-400 | -0.11587 | -0.36199 | 0.130253 | 0.698191 | Abandoned.Field.Pasture |
| 200-100 | -0.54159 | -0.81211 | -0.27107 | 6.72E-07 | Scrubby.Flatwoods |
| 300-100 | -0.99183 | -1.26235 | -0.7213 | 1.01E-10 | Scrubby.Flatwoods |
| 400-100 | -1.78502 | -2.05555 | -1.5145 | 1.01E-10 | Scrubby.Flatwoods |
| 500-100 | -2.27638 | -2.54691 | -2.00586 | 1.01E-10 | Scrubby.Flatwoods |
| 300-200 | -0.45024 | -0.71939 | -0.18108 | 5.77E-05 | Scrubby.Flatwoods |
| 400-200 | -1.24343 | -1.51259 | -0.97428 | 1.01E-10 | Scrubby.Flatwoods |
| 500-200 | -1.73479 | -2.00395 | -1.46564 | 1.01E-10 | Scrubby.Flatwoods |
| 400-300 | -0.7932 | -1.06235 | -0.52404 | 1.02E-10 | Scrubby.Flatwoods |
| 500-300 | -1.28456 | -1.55371 | -1.0154 | 1.01E-10 | Scrubby.Flatwoods |
| 500-400 | -0.49136 | -0.76051 | -0.2222 | 7.96E-06 | Scrubby.Flatwoods |
| 200-100 | -0.0326 | -0.22609 | 0.160891 | 0.990671 | Clastic.Upland.Lake |
| 300-100 | -0.14601 | -0.3395 | 0.047485 | 0.23662 | Clastic.Upland.Lake |
| 400-100 | -0.19772 | -0.39121 | -0.00422 | 0.042429 | Clastic.Upland.Lake |
| 500-100 | -0.35083 | -0.54432 | -0.15734 | 9.41E-06 | Clastic.Upland.Lake |
| 300-200 | -0.11341 | -0.30542 | 0.078608 | 0.487134 | Clastic.Upland.Lake |
| 400-200 | -0.16512 | -0.35713 | 0.026898 | 0.129857 | Clastic.Upland.Lake |
| 500-200 | -0.31823 | -0.51024 | -0.12621 | 7.00E-05 | Clastic.Upland.Lake |
| 400-300 | -0.05171 | -0.24372 | 0.140304 | 0.947662 | Clastic.Upland.Lake |
| 500-300 | -0.20482 | -0.39684 | -0.01281 | 0.029896 | Clastic.Upland.Lake |
| 500-400 | -0.15311 | -0.34513 | 0.038902 | 0.187751 | Clastic.Upland.Lake |

Table S3: Results of Tukey Test comparing the values of PD_SES_ for five randomly sampled subset sizes for 14 communities (Question 1).

| Subsets compared | Difference | Lower 95% confidence interval | Upper 95% confidence interval | Adjusted p-value | Community |
| --- | --- | --- | --- | --- | --- |
| 200-100 | -0.10551 | -0.39392 | 0.182892 | 0.854583 | Basin.Marsh |
| 300-100 | 0.113393 | -0.17501 | 0.401798 | 0.818634 | Basin.Marsh |
| 400-100 | 0.289649 | 0.001243 | 0.578055 | 0.048421 | Basin.Marsh |
| 500-100 | 0.15807 | -0.13034 | 0.446476 | 0.56258 | Basin.Marsh |
| 300-200 | 0.218906 | -0.0695 | 0.507312 | 0.231216 | Basin.Marsh |
| 400-200 | 0.395163 | 0.106757 | 0.683569 | 0.001833 | Basin.Marsh |
| 500-200 | 0.263584 | -0.02482 | 0.55199 | 0.09175 | Basin.Marsh |
| 400-300 | 0.176256 | -0.11215 | 0.464662 | 0.451539 | Basin.Marsh |
| 500-300 | 0.044678 | -0.24373 | 0.333084 | 0.993232 | Basin.Marsh |
| 500-400 | -0.13158 | -0.41998 | 0.156827 | 0.722451 | Basin.Marsh |
| 200-100 | -0.1252 | -0.40511 | 0.154707 | 0.737007 | Basin.Swamp |
| 300-100 | -0.17147 | -0.45138 | 0.108439 | 0.44904 | Basin.Swamp |
| 400-100 | -0.29648 | -0.57639 | -0.01657 | 0.031735 | Basin.Swamp |
| 500-100 | -0.49812 | -0.77804 | -0.21821 | 1.47E-05 | Basin.Swamp |
| 300-200 | -0.04627 | -0.32548 | 0.232939 | 0.991244 | Basin.Swamp |
| 400-200 | -0.17128 | -0.45048 | 0.107929 | 0.447583 | Basin.Swamp |
| 500-200 | -0.37292 | -0.65213 | -0.09371 | 0.00261 | Basin.Swamp |
| 400-300 | -0.12501 | -0.40422 | 0.154197 | 0.736305 | Basin.Swamp |
| 500-300 | -0.32665 | -0.60586 | -0.04745 | 0.012544 | Basin.Swamp |
| 500-400 | -0.20164 | -0.48085 | 0.077564 | 0.278634 | Basin.Swamp |
| 200-100 | -0.30916 | -0.6159 | -0.00243 | 0.047128 | Baygall |
| 300-100 | -0.29913 | -0.60587 | 0.007597 | 0.05994 | Baygall |
| 400-100 | -0.56802 | -0.87475 | -0.26129 | 5.57E-06 | Baygall |
| 500-100 | -0.70178 | -1.00851 | -0.39505 | 8.26E-09 | Baygall |
| 300-200 | 0.010029 | -0.2967 | 0.316761 | 0.999985 | Baygall |
| 400-200 | -0.25886 | -0.56559 | 0.047874 | 0.143198 | Baygall |
| 500-200 | -0.39261 | -0.69935 | -0.08588 | 0.004527 | Baygall |
| 400-300 | -0.26889 | -0.57562 | 0.037845 | 0.116965 | Baygall |
| 500-300 | -0.40264 | -0.70938 | -0.09591 | 0.003285 | Baygall |
| 500-400 | -0.13376 | -0.44049 | 0.172976 | 0.754935 | Baygall |
| 200-100 | -0.23425 | -0.47446 | 0.005951 | 0.059943 | Lake.Bottom |
| 300-100 | -0.69385 | -0.93405 | -0.45365 | 1.10E-10 | Lake.Bottom |
| 400-100 | -0.9719 | -1.2121 | -0.73169 | 1.10E-10 | Lake.Bottom |
| 500-100 | -1.29207 | -1.53227 | -1.05186 | 1.10E-10 | Lake.Bottom |
| 300-200 | -0.4596 | -0.6998 | -0.2194 | 2.38E-06 | Lake.Bottom |
| 400-200 | -0.73764 | -0.97785 | -0.49744 | 1.10E-10 | Lake.Bottom |
| 500-200 | -1.05781 | -1.29802 | -0.81761 | 1.10E-10 | Lake.Bottom |
| 400-300 | -0.27804 | -0.51825 | -0.03784 | 0.013985 | Lake.Bottom |
| 500-300 | -0.59821 | -0.83842 | -0.35801 | 3.79E-10 | Lake.Bottom |
| 500-400 | -0.32017 | -0.56037 | -0.07997 | 0.002683 | Lake.Bottom |
| 200-100 | -0.16649 | -0.3824 | 0.049413 | 0.216836 | Mesic.Hammock |
| 300-100 | -0.34451 | -0.56042 | -0.1286 | 0.000148 | Mesic.Hammock |
| 400-100 | -0.4467 | -0.66261 | -0.23079 | 2.50E-07 | Mesic.Hammock |
| 500-100 | -0.71474 | -0.93065 | -0.49884 | 1.10E-10 | Mesic.Hammock |
| 300-200 | -0.17802 | -0.39392 | 0.03789 | 0.160731 | Mesic.Hammock |
| 400-200 | -0.28021 | -0.49611 | -0.0643 | 0.003804 | Mesic.Hammock |
| 500-200 | -0.54825 | -0.76416 | -0.33234 | 2.24E-10 | Mesic.Hammock |
| 400-300 | -0.10219 | -0.3181 | 0.113715 | 0.69391 | Mesic.Hammock |
| 500-300 | -0.37023 | -0.58614 | -0.15433 | 3.40E-05 | Mesic.Hammock |
| 500-400 | -0.26804 | -0.48395 | -0.05214 | 0.006535 | Mesic.Hammock |
| 200-100 | -0.44684 | -0.64661 | -0.24707 | 1.89E-08 | Pine.Plantation |
| 300-100 | -0.84326 | -1.04303 | -0.64349 | 6.24E-11 | Pine.Plantation |
| 400-100 | -1.14542 | -1.34519 | -0.94565 | 6.24E-11 | Pine.Plantation |
| 500-100 | -1.44116 | -1.64093 | -1.24139 | 6.24E-11 | Pine.Plantation |
| 300-200 | -0.39642 | -0.58971 | -0.20313 | 3.30E-07 | Pine.Plantation |
| 400-200 | -0.69858 | -0.89187 | -0.50529 | 6.25E-11 | Pine.Plantation |
| 500-200 | -0.99432 | -1.18761 | -0.80103 | 6.24E-11 | Pine.Plantation |
| 400-300 | -0.30216 | -0.49545 | -0.10887 | 0.000218 | Pine.Plantation |
| 500-300 | -0.5979 | -0.79119 | -0.40461 | 6.25E-11 | Pine.Plantation |
| 500-400 | -0.29574 | -0.48903 | -0.10245 | 0.00032 | Pine.Plantation |
| 200-100 | -1.02755 | -1.3074 | -0.74771 | 1.10E-10 | Sandhill |
| 300-100 | -1.99593 | -2.27578 | -1.71609 | 1.10E-10 | Sandhill |
| 400-100 | -2.80082 | -3.08066 | -2.52097 | 1.10E-10 | Sandhill |
| 500-100 | -3.32045 | -3.6003 | -3.04061 | 1.10E-10 | Sandhill |
| 300-200 | -0.96838 | -1.24823 | -0.68854 | 1.10E-10 | Sandhill |
| 400-200 | -1.77327 | -2.05311 | -1.49342 | 1.10E-10 | Sandhill |
| 500-200 | -2.2929 | -2.57274 | -2.01305 | 1.10E-10 | Sandhill |
| 400-300 | -0.80489 | -1.08473 | -0.52504 | 1.11E-10 | Sandhill |
| 500-300 | -1.32452 | -1.60436 | -1.04467 | 1.10E-10 | Sandhill |
| 500-400 | -0.51963 | -0.79948 | -0.23979 | 5.20E-06 | Sandhill |
| 200-100 | 0.022496 | -0.21017 | 0.255167 | 0.998919 | Sandhill.Upland.Lake |
| 300-100 | -0.2256 | -0.45827 | 0.007067 | 0.062405 | Sandhill.Upland.Lake |
| 400-100 | -0.53482 | -0.76749 | -0.30215 | 6.95E-09 | Sandhill.Upland.Lake |
| 500-100 | -0.99962 | -1.23229 | -0.76695 | 1.10E-10 | Sandhill.Upland.Lake |
| 300-200 | -0.2481 | -0.48077 | -0.01543 | 0.029988 | Sandhill.Upland.Lake |
| 400-200 | -0.55732 | -0.78999 | -0.32465 | 1.48E-09 | Sandhill.Upland.Lake |
| 500-200 | -1.02212 | -1.25479 | -0.78945 | 1.10E-10 | Sandhill.Upland.Lake |
| 400-300 | -0.30922 | -0.54189 | -0.07655 | 0.00279 | Sandhill.Upland.Lake |
| 500-300 | -0.77402 | -1.00669 | -0.54135 | 1.10E-10 | Sandhill.Upland.Lake |
| 500-400 | -0.4648 | -0.69747 | -0.23213 | 7.14E-07 | Sandhill.Upland.Lake |
| 200-100 | 0.083979 | -0.15908 | 0.327038 | 0.878715 | Succesional.Hardwood.Forest |
| 300-100 | 0.145978 | -0.09708 | 0.389037 | 0.469752 | Succesional.Hardwood.Forest |
| 400-100 | 0.229534 | -0.01352 | 0.472593 | 0.074583 | Succesional.Hardwood.Forest |
| 500-100 | 0.110026 | -0.13303 | 0.353085 | 0.728276 | Succesional.Hardwood.Forest |
| 300-200 | 0.061999 | -0.18106 | 0.305058 | 0.956801 | Succesional.Hardwood.Forest |
| 400-200 | 0.145555 | -0.0975 | 0.388613 | 0.472778 | Succesional.Hardwood.Forest |
| 500-200 | 0.026047 | -0.21701 | 0.269106 | 0.998381 | Succesional.Hardwood.Forest |
| 400-300 | 0.083556 | -0.1595 | 0.326615 | 0.880672 | Succesional.Hardwood.Forest |
| 500-300 | -0.03595 | -0.27901 | 0.207107 | 0.994333 | Succesional.Hardwood.Forest |
| 500-400 | -0.11951 | -0.36257 | 0.123551 | 0.662398 | Succesional.Hardwood.Forest |
| 200-100 | -0.41292 | -0.71827 | -0.10757 | 0.002204 | Xeric.Hammock |
| 300-100 | -0.60777 | -0.91312 | -0.30243 | 7.94E-07 | Xeric.Hammock |
| 400-100 | -0.7634 | -1.06875 | -0.45805 | 3.37E-10 | Xeric.Hammock |
| 500-100 | -1.31681 | -1.62216 | -1.01146 | 1.10E-10 | Xeric.Hammock |
| 300-200 | -0.19486 | -0.5002 | 0.110492 | 0.406037 | Xeric.Hammock |
| 400-200 | -0.35048 | -0.65583 | -0.04514 | 0.015218 | Xeric.Hammock |
| 500-200 | -0.90389 | -1.20924 | -0.59855 | 1.10E-10 | Xeric.Hammock |
| 400-300 | -0.15563 | -0.46097 | 0.14972 | 0.63091 | Xeric.Hammock |
| 500-300 | -0.70904 | -1.01438 | -0.40369 | 4.77E-09 | Xeric.Hammock |
| 500-400 | -0.55341 | -0.85876 | -0.24806 | 9.49E-06 | Xeric.Hammock |
| 200-100 | -0.62843 | -0.80519 | -0.45167 | 1.10E-10 | Improved |
| 300-100 | -1.21501 | -1.39177 | -1.03824 | 1.10E-10 | Improved |
| 400-100 | -1.66417 | -1.84093 | -1.4874 | 1.10E-10 | Improved |
| 500-100 | -2.03839 | -2.21515 | -1.86162 | 1.10E-10 | Improved |
| 300-200 | -0.58658 | -0.76334 | -0.40981 | 1.10E-10 | Improved |
| 400-200 | -1.03574 | -1.2125 | -0.85897 | 1.10E-10 | Improved |
| 500-200 | -1.40996 | -1.58672 | -1.23319 | 1.10E-10 | Improved |
| 400-300 | -0.44916 | -0.62593 | -0.2724 | 2.21E-10 | Improved |
| 500-300 | -0.82338 | -1.00015 | -0.64662 | 1.10E-10 | Improved |
| 500-400 | -0.37422 | -0.55098 | -0.19746 | 1.21E-07 | Improved |
| 200-100 | -0.09582 | -0.29812 | 0.106469 | 0.693278 | Abandoned.Field.Pasture |
| 300-100 | -0.44244 | -0.64473 | -0.24014 | 4.09E-08 | Abandoned.Field.Pasture |
| 400-100 | -0.54178 | -0.74407 | -0.33948 | 1.20E-10 | Abandoned.Field.Pasture |
| 500-100 | -0.66393 | -0.86622 | -0.46164 | 1.10E-10 | Abandoned.Field.Pasture |
| 300-200 | -0.34661 | -0.54891 | -0.14432 | 3.46E-05 | Abandoned.Field.Pasture |
| 400-200 | -0.44595 | -0.64825 | -0.24366 | 3.11E-08 | Abandoned.Field.Pasture |
| 500-200 | -0.56811 | -0.7704 | -0.36582 | 1.11E-10 | Abandoned.Field.Pasture |
| 400-300 | -0.09934 | -0.30163 | 0.102951 | 0.663445 | Abandoned.Field.Pasture |
| 500-300 | -0.2215 | -0.42379 | -0.0192 | 0.023781 | Abandoned.Field.Pasture |
| 500-400 | -0.12215 | -0.32445 | 0.080139 | 0.464108 | Abandoned.Field.Pasture |
| 200-100 | -0.59962 | -0.79329 | -0.40595 | 1.01E-10 | Scrubby.Flatwoods |
| 300-100 | -1.08075 | -1.27442 | -0.88708 | 1.01E-10 | Scrubby.Flatwoods |
| 400-100 | -1.64512 | -1.83879 | -1.45145 | 1.01E-10 | Scrubby.Flatwoods |
| 500-100 | -2.10343 | -2.2971 | -1.90976 | 1.01E-10 | Scrubby.Flatwoods |
| 300-200 | -0.48113 | -0.67382 | -0.28844 | 3.42E-10 | Scrubby.Flatwoods |
| 400-200 | -1.0455 | -1.23819 | -0.85281 | 1.01E-10 | Scrubby.Flatwoods |
| 500-200 | -1.50381 | -1.6965 | -1.31112 | 1.01E-10 | Scrubby.Flatwoods |
| 400-300 | -0.56437 | -0.75706 | -0.37168 | 1.02E-10 | Scrubby.Flatwoods |
| 500-300 | -1.02269 | -1.21538 | -0.83 | 1.01E-10 | Scrubby.Flatwoods |
| 500-400 | -0.45832 | -0.65101 | -0.26563 | 1.93E-09 | Scrubby.Flatwoods |
| 200-100 | -0.30783 | -0.49022 | -0.12545 | 4.78E-05 | Clastic.Upland.Lake |
| 300-100 | -0.46598 | -0.64836 | -0.28359 | 1.84E-10 | Clastic.Upland.Lake |
| 400-100 | -0.61046 | -0.79285 | -0.42808 | 9.73E-11 | Clastic.Upland.Lake |
| 500-100 | -0.73633 | -0.91872 | -0.55395 | 9.72E-11 | Clastic.Upland.Lake |
| 300-200 | -0.15815 | -0.33914 | 0.022845 | 0.119082 | Clastic.Upland.Lake |
| 400-200 | -0.30263 | -0.48362 | -0.12164 | 5.83E-05 | Clastic.Upland.Lake |
| 500-200 | -0.4285 | -0.60949 | -0.24751 | 2.30E-09 | Clastic.Upland.Lake |
| 400-300 | -0.14448 | -0.32547 | 0.036507 | 0.186811 | Clastic.Upland.Lake |
| 500-300 | -0.27035 | -0.45134 | -0.08936 | 0.000483 | Clastic.Upland.Lake |
| 500-400 | -0.12587 | -0.30686 | 0.055124 | 0.316477 | Clastic.Upland.Lake |

Table S4: Numbers and proportions of replicates showing significant phylogenetic patterns of NRI for pruned phylogenies of randomly sampled subsets for each community (Question 1). Results are separated into clustered and overdispersed patterns.

| Community | Subset | Number Clustered | Proportion Clustered | Number Overdispersed | Proportion Overdispersed |
| --- | --- | --- | --- | --- | --- |
| Basin.Marsh | 100 | 15 | 0.15 | 2 | 0.02 |
| Basin.Swamp | 100 | 11 | 0.11 | 31 | 0.31 |
| Baygall | 100 | 36 | 0.36 | 3 | 0.03 |
| Lake.Bottom | 100 | 1 | 0.01 | 5 | 0.05 |
| Mesic.Hammock | 100 | 12 | 0.12 | 10 | 0.1 |
| Pine.Plantation | 100 | 10 | 0.1 | 2 | 0.02 |
| Sandhill | 100 | 23 | 0.23 | 0 | 0 |
| Sandhill.Upland.Lake | 100 | 0 | 0 | 21 | 0.21 |
| Succesional.Hardwood.Forest | 100 | 3 | 0.03 | 4 | 0.04 |
| Xeric.Hammock | 100 | 39 | 0.39 | 0 | 0 |
| Improved | 100 | 4 | 0.04 | 0 | 0 |
| Abandoned.Field.Pasture | 100 | 18 | 0.18 | 0 | 0 |
| Scrubby.Flatwoods | 100 | 26 | 0.26 | 0 | 0 |
| Clastic.Upland.Lake | 100 | 15 | 0.15 | 0 | 0 |
| Basin.Marsh | 200 | 19 | 0.19 | 0 | 0 |
| Basin.Swamp | 200 | 6 | 0.06 | 33 | 0.33 |
| Baygall | 200 | 56 | 0.56 | 0 | 0 |
| Lake.Bottom | 200 | 1 | 0.01 | 5 | 0.05 |
| Mesic.Hammock | 200 | 9 | 0.09 | 5 | 0.05 |
| Pine.Plantation | 200 | 15 | 0.15 | 0 | 0 |
| Sandhill | 200 | 42 | 0.42 | 0 | 0 |
| Sandhill.Upland.Lake | 200 | 0 | 0 | 24 | 0.24 |
| Succesional.Hardwood.Forest | 200 | 4 | 0.04 | 7 | 0.07 |
| Xeric.Hammock | 200 | 49 | 0.49 | 0 | 0 |
| Improved | 200 | 4 | 0.04 | 0 | 0 |
| Abandoned.Field.Pasture | 200 | 7 | 0.07 | 0 | 0 |
| Scrubby.Flatwoods | 200 | 37 | 0.37 | 0 | 0 |
| Clastic.Upland.Lake | 200 | 24 | 0.24 | 0 | 0 |
| Basin.Marsh | 300 | 13 | 0.13 | 0 | 0 |
| Basin.Swamp | 300 | 2 | 0.02 | 53 | 0.53 |
| Baygall | 300 | 39 | 0.39 | 0 | 0 |
| Lake.Bottom | 300 | 0 | 0 | 0 | 0 |
| Mesic.Hammock | 300 | 2 | 0.02 | 7 | 0.07 |
| Pine.Plantation | 300 | 13 | 0.13 | 0 | 0 |
| Sandhill | 300 | 63 | 0.63 | 0 | 0 |
| Sandhill.Upland.Lake | 300 | 0 | 0 | 29 | 0.29 |
| Succesional.Hardwood.Forest | 300 | 1 | 0.01 | 8 | 0.08 |
| Xeric.Hammock | 300 | 37 | 0.37 | 0 | 0 |
| Improved | 300 | 2 | 0.02 | 0 | 0 |
| Abandoned.Field.Pasture | 300 | 20 | 0.2 | 0 | 0 |
| Scrubby.Flatwoods | 300 | 36 | 0.36 | 0 | 0 |
| Clastic.Upland.Lake | 300 | 38 | 0.38 | 0 | 0 |
| Basin.Marsh | 400 | 6 | 0.06 | 0 | 0 |
| Basin.Swamp | 400 | 0 | 0 | 71 | 0.71 |
| Baygall | 400 | 48 | 0.48 | 0 | 0 |
| Lake.Bottom | 400 | 0 | 0 | 0 | 0 |
| Mesic.Hammock | 400 | 1 | 0.01 | 8 | 0.08 |
| Pine.Plantation | 400 | 8 | 0.08 | 0 | 0 |
| Sandhill | 400 | 95 | 0.95 | 0 | 0 |
| Sandhill.Upland.Lake | 400 | 0 | 0 | 40 | 0.4 |
| Succesional.Hardwood.Forest | 400 | 0 | 0 | 4 | 0.04 |
| Xeric.Hammock | 400 | 34 | 0.34 | 0 | 0 |
| Improved | 400 | 6 | 0.06 | 0 | 0 |
| Abandoned.Field.Pasture | 400 | 13 | 0.13 | 0 | 0 |
| Scrubby.Flatwoods | 400 | 53 | 0.53 | 0 | 0 |
| Clastic.Upland.Lake | 400 | 68 | 0.68 | 0 | 0 |
| Basin.Marsh | 500 | 13 | 0.13 | 0 | 0 |
| Basin.Swamp | 500 | 0 | 0 | 76 | 0.76 |
| Baygall | 500 | 82 | 0.82 | 0 | 0 |
| Lake.Bottom | 500 | 0 | 0 | 0 | 0 |
| Mesic.Hammock | 500 | 0 | 0 | 0 | 0 |
| Pine.Plantation | 500 | 7 | 0.07 | 0 | 0 |
| Sandhill | 500 | 100 | 1 | 0 | 0 |
| Sandhill.Upland.Lake | 500 | 0 | 0 | 36 | 0.36 |
| Succesional.Hardwood.Forest | 500 | 0 | 0 | 0 | 0 |
| Xeric.Hammock | 500 | 72 | 0.72 | 0 | 0 |
| Improved | 500 | 1 | 0.01 | 0 | 0 |
| Abandoned.Field.Pasture | 500 | 8 | 0.08 | 0 | 0 |
| Scrubby.Flatwoods | 500 | 78 | 0.78 | 0 | 0 |
| Clastic.Upland.Lake | 500 | 88 | 0.88 | 0 | 0 |

Table S5: Numbers and proportions of replicates showing significant phylogenetic patterns of NTI for pruned phylogenies of randomly sampled subsets for each community (Question 1). Results are separated into clustered and overdispersed patterns.

| Community | Subset | Number Overdispersed | Proportion Overdispersed | Number  Clustered | Proportion Clustered |
| --- | --- | --- | --- | --- | --- |
| Basin.Marsh | 100 | 8 | 0.08 | 1 | 0.01 |
| Basin.Swamp | 100 | 15 | 0.15 | 4 | 0.04 |
| Baygall | 100 | 2 | 0.02 | 18 | 0.18 |
| Lake.Bottom | 100 | 0 | 0 | 11 | 0.11 |
| Mesic.Hammock | 100 | 4 | 0.04 | 2 | 0.02 |
| Pine.Plantation | 100 | 2 | 0.02 | 17 | 0.17 |
| Sandhill | 100 | 0 | 0 | 42 | 0.42 |
| Sandhill.Upland.Lake | 100 | 11 | 0.11 | 3 | 0.03 |
| Succesional.Hardwood.Forest | 100 | 4 | 0.04 | 5 | 0.05 |
| Xeric.Hammock | 100 | 0 | 0 | 33 | 0.33 |
| Improved | 100 | 0 | 0 | 8 | 0.08 |
| Abandoned.Field.Pasture | 100 | 3 | 0.03 | 4 | 0.04 |
| Scrubby.Flatwoods | 100 | 0 | 0 | 29 | 0.29 |
| Clastic.Upland.Lake | 100 | 0 | 0 | 7 | 0.07 |
| Basin.Marsh | 200 | 8 | 0.08 | 2 | 0.02 |
| Basin.Swamp | 200 | 5 | 0.05 | 2 | 0.02 |
| Baygall | 200 | 0 | 0 | 20 | 0.2 |
| Lake.Bottom | 200 | 0 | 0 | 17 | 0.17 |
| Mesic.Hammock | 200 | 0 | 0 | 6 | 0.06 |
| Pine.Plantation | 200 | 0 | 0 | 26 | 0.26 |
| Sandhill | 200 | 0 | 0 | 73 | 0.73 |
| Sandhill.Upland.Lake | 200 | 11 | 0.11 | 3 | 0.03 |
| Succesional.Hardwood.Forest | 200 | 6 | 0.06 | 3 | 0.03 |
| Xeric.Hammock | 200 | 0 | 0 | 47 | 0.47 |
| Improved | 200 | 0 | 0 | 28 | 0.28 |
| Abandoned.Field.Pasture | 200 | 0 | 0 | 2 | 0.02 |
| Scrubby.Flatwoods | 200 | 0 | 0 | 50 | 0.5 |
| Clastic.Upland.Lake | 200 | 0 | 0 | 5 | 0.05 |
| Basin.Marsh | 300 | 10 | 0.1 | 1 | 0.01 |
| Basin.Swamp | 300 | 1 | 0.01 | 2 | 0.02 |
| Baygall | 300 | 0 | 0 | 22 | 0.22 |
| Lake.Bottom | 300 | 0 | 0 | 35 | 0.35 |
| Mesic.Hammock | 300 | 0 | 0 | 18 | 0.18 |
| Pine.Plantation | 300 | 0 | 0 | 44 | 0.44 |
| Sandhill | 300 | 0 | 0 | 93 | 0.93 |
| Sandhill.Upland.Lake | 300 | 0 | 0 | 23 | 0.23 |
| Succesional.Hardwood.Forest | 300 | 2 | 0.02 | 0 | 0 |
| Xeric.Hammock | 300 | 0 | 0 | 37 | 0.37 |
| Improved | 300 | 0 | 0 | 48 | 0.48 |
| Abandoned.Field.Pasture | 300 | 0 | 0 | 3 | 0.03 |
| Scrubby.Flatwoods | 300 | 0 | 0 | 72 | 0.72 |
| Clastic.Upland.Lake | 300 | 0 | 0 | 9 | 0.09 |
| Basin.Marsh | 400 | 5 | 0.05 | 0 | 0 |
| Basin.Swamp | 400 | 0 | 0 | 0 | 0 |
| Baygall | 400 | 0 | 0 | 31 | 0.31 |
| Lake.Bottom | 400 | 0 | 0 | 53 | 0.53 |
| Mesic.Hammock | 400 | 0 | 0 | 16 | 0.16 |
| Pine.Plantation | 400 | 0 | 0 | 67 | 0.67 |
| Sandhill | 400 | 0 | 0 | 98 | 0.98 |
| Sandhill.Upland.Lake | 400 | 0 | 0 | 64 | 0.64 |
| Succesional.Hardwood.Forest | 400 | 0 | 0 | 0 | 0 |
| Xeric.Hammock | 400 | 0 | 0 | 44 | 0.44 |
| Improved | 400 | 0 | 0 | 73 | 0.73 |
| Abandoned.Field.Pasture | 400 | 0 | 0 | 1 | 0.01 |
| Scrubby.Flatwoods | 400 | 0 | 0 | 97 | 0.97 |
| Clastic.Upland.Lake | 400 | 0 | 0 | 5 | 0.05 |
| Basin.Marsh | 500 | 0 | 0 | 0 | 0 |
| Basin.Swamp | 500 | 0 | 0 | 0 | 0 |
| Baygall | 500 | 0 | 0 | 19 | 0.19 |
| Lake.Bottom | 500 | 0 | 0 | 81 | 0.81 |
| Mesic.Hammock | 500 | 0 | 0 | 16 | 0.16 |
| Pine.Plantation | 500 | 0 | 0 | 93 | 0.93 |
| Sandhill | 500 | 0 | 0 | 100 | 1 |
| Sandhill.Upland.Lake | 500 | 0 | 0 | 98 | 0.98 |
| Succesional.Hardwood.Forest | 500 | 0 | 0 | 0 | 0 |
| Xeric.Hammock | 500 | 0 | 0 | 86 | 0.86 |
| Improved | 500 | 0 | 0 | 93 | 0.93 |
| Abandoned.Field.Pasture | 500 | 0 | 0 | 1 | 0.01 |
| Scrubby.Flatwoods | 500 | 0 | 0 | 100 | 1 |
| Clastic.Upland.Lake | 500 | 0 | 0 | 1 | 0.01 |

Table S6: Numbers and proportions of replicates showing significant phylogenetic patterns of PD_SES_ for pruned phylogenies of randomly sampled subsets for each community (Question 1). Results are separated into clustered and overdispersed patterns.

| Community | Subset | Number Overdispersed | Proportion Overdispersed | Number Clustered | Proportion Clustered |
| --- | --- | --- | --- | --- | --- |
| Basin.Marsh | 100 | 6 | 0.06 | 0 | 0 |
| Basin.Swamp | 100 | 21 | 0.21 | 2 | 0.02 |
| Baygall | 100 | 6 | 0.06 | 4 | 0.04 |
| Lake.Bottom | 100 | 1 | 0.01 | 1 | 0.01 |
| Mesic.Hammock | 100 | 5 | 0.05 | 0 | 0 |
| Pine.Plantation | 100 | 11 | 0.11 | 1 | 0.01 |
| Sandhill | 100 | 0 | 0 | 13 | 0.13 |
| Sandhill.Upland.Lake | 100 | 20 | 0.2 | 0 | 0 |
| Succesional.Hardwood.Forest | 100 | 8 | 0.08 | 0 | 0 |
| Xeric.Hammock | 100 | 0 | 0 | 11 | 0.11 |
| Improved | 100 | 0 | 0 | 1 | 0.01 |
| Abandoned.Field.Pasture | 100 | 3 | 0.03 | 1 | 0.01 |
| Scrubby.Flatwoods | 100 | 5 | 0.05 | 0 | 0 |
| Clastic.Upland.Lake | 100 | 5 | 0.05 | 3 | 0.03 |
| Basin.Marsh | 200 | 5 | 0.05 | 0 | 0 |
| Basin.Swamp | 200 | 7 | 0.07 | 0 | 0 |
| Baygall | 200 | 0 | 0 | 4 | 0.04 |
| Lake.Bottom | 200 | 3 | 0.03 | 0 | 0 |
| Mesic.Hammock | 200 | 5 | 0.05 | 0 | 0 |
| Pine.Plantation | 200 | 1 | 0.01 | 0 | 0 |
| Sandhill | 200 | 0 | 0 | 59 | 0.59 |
| Sandhill.Upland.Lake | 200 | 30 | 0.3 | 0 | 0 |
| Succesional.Hardwood.Forest | 200 | 17 | 0.17 | 0 | 0 |
| Xeric.Hammock | 200 | 0 | 0 | 20 | 0.2 |
| Improved | 200 | 0 | 0 | 4 | 0.04 |
| Abandoned.Field.Pasture | 200 | 0 | 0 | 0 | 0 |
| Scrubby.Flatwoods | 200 | 0 | 0 | 3 | 0.03 |
| Clastic.Upland.Lake | 200 | 0 | 0 | 0 | 0 |
| Basin.Marsh | 300 | 10 | 0.1 | 0 | 0 |
| Basin.Swamp | 300 | 6 | 0.06 | 0 | 0 |
| Baygall | 300 | 0 | 0 | 6 | 0.06 |
| Lake.Bottom | 300 | 0 | 0 | 4 | 0.04 |
| Mesic.Hammock | 300 | 1 | 0.01 | 0 | 0 |
| Pine.Plantation | 300 | 0 | 0 | 0 | 0 |
| Sandhill | 300 | 0 | 0 | 94 | 0.94 |
| Sandhill.Upland.Lake | 300 | 8 | 0.08 | 0 | 0 |
| Succesional.Hardwood.Forest | 300 | 17 | 0.17 | 0 | 0 |
| Xeric.Hammock | 300 | 0 | 0 | 21 | 0.21 |
| Improved | 300 | 0 | 0 | 25 | 0.25 |
| Abandoned.Field.Pasture | 300 | 0 | 0 | 0 | 0 |
| Scrubby.Flatwoods | 300 | 0 | 0 | 16 | 0.16 |
| Clastic.Upland.Lake | 300 | 0 | 0 | 0 | 0 |
| Basin.Marsh | 400 | 8 | 0.08 | 0 | 0 |
| Basin.Swamp | 400 | 0 | 0 | 0 | 0 |
| Baygall | 400 | 0 | 0 | 13 | 0.13 |
| Lake.Bottom | 400 | 0 | 0 | 0 | 0 |
| Mesic.Hammock | 400 | 0 | 0 | 0 | 0 |
| Pine.Plantation | 400 | 0 | 0 | 0 | 0 |
| Sandhill | 400 | 0 | 0 | 100 | 1 |
| Sandhill.Upland.Lake | 400 | 1 | 0.01 | 0 | 0 |
| Succesional.Hardwood.Forest | 400 | 7 | 0.07 | 0 | 0 |
| Xeric.Hammock | 400 | 0 | 0 | 15 | 0.15 |
| Improved | 400 | 0 | 0 | 53 | 0.53 |
| Abandoned.Field.Pasture | 400 | 0 | 0 | 0 | 0 |
| Scrubby.Flatwoods | 400 | 0 | 0 | 56 | 0.56 |
| Clastic.Upland.Lake | 400 | 0 | 0 | 0 | 0 |
| Basin.Marsh | 500 | 1 | 0.01 | 0 | 0 |
| Basin.Swamp | 500 | 0 | 0 | 0 | 0 |
| Baygall | 500 | 0 | 0 | 11 | 0.11 |
| Lake.Bottom | 500 | 0 | 0 | 7 | 0.07 |
| Mesic.Hammock | 500 | 0 | 0 | 0 | 0 |
| Pine.Plantation | 500 | 0 | 0 | 0 | 0 |
| Sandhill | 500 | 0 | 0 | 100 | 1 |
| Sandhill.Upland.Lake | 500 | 0 | 0 | 0 | 0 |
| Succesional.Hardwood.Forest | 500 | 1 | 0.01 | 0 | 0 |
| Xeric.Hammock | 500 | 0 | 0 | 20 | 0.2 |
| Improved | 500 | 0 | 0 | 97 | 0.97 |
| Abandoned.Field.Pasture | 500 | 0 | 0 | 1 | 0.01 |
| Scrubby.Flatwoods | 500 | 0 | 0 | 98 | 0.98 |
| Clastic.Upland.Lake | 500 | 0 | 0 | 0 | 0 |

Table S7: Results of Student’s t-test comparing the values of NRI for randomly sampled and targeted subsets for 14 communities (Question 2).

| Community | Subset (targeted) | Subset (random) | P-value | Statistic |
| --- | --- | --- | --- | --- |
| Basin.Marsh | 89 | 100 | 0.121198 | -1.55645 |
| Basin.Swamp | 89 | 100 | 6.18E-12 | -7.46087 |
| Baygall | 89 | 100 | 0.017275 | -2.40187 |
| Lake.Bottom | 89 | 100 | 0.013588 | 2.491616 |
| Mesic.Hammock | 89 | 100 | 3.04E-15 | -8.62424 |
| Pine.Plantation | 89 | 100 | 1.45E-06 | 4.982017 |
| Sandhill | 89 | 100 | 0.81474 | 0.234634 |
| Sandhill.Upland.Lake | 89 | 100 | 0.43721 | 0.778577 |
| Succesional.Hardwood.Forest | 89 | 100 | 0.595974 | 0.531089 |
| Xeric.Hammock | 89 | 100 | 0.09636 | -1.67108 |
| Improved | 89 | 100 | 0.006331 | 2.761487 |
| Abandoned.Field.Pasture | 89 | 100 | 0.654652 | 0.448053 |
| Scrubby.Flatwoods | 89 | 100 | 0.030343 | 2.184141 |
| Clastic.Upland.Lake | 89 | 100 | 6.90E-16 | 8.957288 |
| Basin.Marsh | 186 | 200 | 1.07E-07 | 5.520451 |
| Basin.Swamp | 186 | 200 | 5.32E-09 | 6.128744 |
| Baygall | 186 | 200 | 0.000537 | 3.519111 |
| Lake.Bottom | 186 | 200 | 6.27E-08 | -5.72093 |
| Mesic.Hammock | 186 | 200 | 1.13E-07 | 5.582078 |
| Pine.Plantation | 186 | 200 | 0.285736 | 1.070429 |
| Sandhill | 186 | 200 | 0.002631 | -3.05128 |
| Sandhill.Upland.Lake | 186 | 200 | 3.77E-07 | 5.268212 |
| Succesional.Hardwood.Forest | 186 | 200 | 6.54E-05 | -4.08139 |
| Xeric.Hammock | 186 | 200 | 1.66E-05 | 4.414806 |
| Improved | 186 | 200 | 9.06E-05 | 4.003215 |
| Abandoned.Field.Pasture | 186 | 200 | 0.004675 | 2.862431 |
| Scrubby.Flatwoods | 186 | 200 | 0.245528 | 1.164778 |
| Clastic.Upland.Lake | 186 | 200 | 0.347368 | 0.941965 |
| Basin.Marsh | 328 | 300 | 0.002983 | -3.0073 |
| Basin.Swamp | 328 | 300 | 0.018192 | -2.38252 |
| Baygall | 328 | 300 | 5.91E-06 | -4.66204 |
| Lake.Bottom | 328 | 300 | 2.19E-07 | 5.452131 |
| Mesic.Hammock | 328 | 300 | 0.008082 | -2.68779 |
| Pine.Plantation | 328 | 300 | 3.47E-05 | -4.24984 |
| Sandhill | 328 | 300 | 0.920226 | 0.100304 |
| Sandhill.Upland.Lake | 328 | 300 | 2.04E-07 | -5.41668 |
| Succesional.Hardwood.Forest | 328 | 300 | 2.58E-06 | 4.875076 |
| Xeric.Hammock | 328 | 300 | 0.102407 | -1.64093 |
| Improved | 328 | 300 | 3.55E-06 | -4.78009 |
| Abandoned.Field.Pasture | 328 | 300 | 0.00058 | -3.50042 |
| Scrubby.Flatwoods | 328 | 300 | 0.137018 | -1.49305 |
| Clastic.Upland.Lake | 328 | 300 | 0.000157 | -3.85854 |
| Basin.Marsh | 397 | 400 | 1.46E-07 | -5.45703 |
| Basin.Swamp | 397 | 400 | 3.52E-06 | -4.77395 |
| Baygall | 397 | 400 | 0.098881 | -1.6581 |
| Lake.Bottom | 397 | 400 | 1.81E-09 | 6.431825 |
| Mesic.Hammock | 397 | 400 | 9.79E-06 | -4.58434 |
| Pine.Plantation | 397 | 400 | 0.674804 | 0.420276 |
| Sandhill | 397 | 400 | 0.000977 | 3.353644 |
| Sandhill.Upland.Lake | 397 | 400 | 5.33E-11 | -6.94995 |
| Succesional.Hardwood.Forest | 397 | 400 | 0.003396 | 2.966419 |
| Xeric.Hammock | 397 | 400 | 1.38E-06 | -4.98283 |
| Improved | 397 | 400 | 0.538882 | -0.61563 |
| Abandoned.Field.Pasture | 397 | 400 | 0.000593 | -3.49119 |
| Scrubby.Flatwoods | 397 | 400 | 0.746408 | 0.323868 |
| Clastic.Upland.Lake | 397 | 400 | 0.240293 | 1.178116 |
| Basin.Marsh | 510 | 500 | 0.633736 | 0.477638 |
| Basin.Swamp | 510 | 500 | 0.132211 | 1.5166 |
| Baygall | 510 | 500 | 0.466204 | -0.73079 |
| Lake.Bottom | 510 | 500 | 0.337859 | -0.96153 |
| Mesic.Hammock | 510 | 500 | 0.002781 | 3.051643 |
| Pine.Plantation | 510 | 500 | 0.14208 | -1.47477 |
| Sandhill | 510 | 500 | 0.068705 | -1.83231 |
| Sandhill.Upland.Lake | 510 | 500 | 0.067271 | 1.846574 |
| Succesional.Hardwood.Forest | 510 | 500 | 0.379912 | 0.881125 |
| Xeric.Hammock | 510 | 500 | 0.001489 | 3.248237 |
| Improved | 510 | 500 | 0.258633 | -1.13371 |
| Abandoned.Field.Pasture | 510 | 500 | 0.008016 | -2.68365 |
| Scrubby.Flatwoods | 510 | 500 | 0.031619 | -2.16499 |
| Clastic.Upland.Lake | 510 | 500 | 7.47E-10 | -6.52479 |

Table S8: Results of Student’s t-test comparing the values of NTI for randomly sampled and targeted subsets for 14 communities (Question 2).

| Community | Subset (targeted) | Subset (random) | P-value | Statistic |
| --- | --- | --- | --- | --- |
| Basin.Marsh | 89 | 100 | 0.908537 | -0.11503 |
| Basin.Swamp | 89 | 100 | 1.37E-07 | -5.52089 |
| Baygall | 89 | 100 | 0.112243 | 1.596706 |
| Lake.Bottom | 89 | 100 | 1.81E-07 | 5.409466 |
| Mesic.Hammock | 89 | 100 | 0.315127 | 1.007079 |
| Pine.Plantation | 89 | 100 | 5.45E-09 | 6.13204 |
| Sandhill | 89 | 100 | 0.029622 | 2.190977 |
| Sandhill.Upland.Lake | 89 | 100 | 6.57E-06 | 4.632961 |
| Succesional.Hardwood.Forest | 89 | 100 | 0.047508 | 1.994318 |
| Xeric.Hammock | 89 | 100 | 0.692413 | 0.396163 |
| Improved | 89 | 100 | 0.000423 | -3.58655 |
| Abandoned.Field.Pasture | 89 | 100 | 0.117828 | 1.570794 |
| Scrubby.Flatwoods | 89 | 100 | 0.009606 | 2.617157 |
| Clastic.Upland.Lake | 89 | 100 | 5.61E-12 | 7.468122 |
| Basin.Marsh | 186 | 200 | 0.003636 | 2.943542 |
| Basin.Swamp | 186 | 200 | 2.94E-05 | 4.2826 |
| Baygall | 186 | 200 | 0.03631 | 2.10781 |
| Lake.Bottom | 186 | 200 | 0.032168 | -2.15985 |
| Mesic.Hammock | 186 | 200 | 0.084 | -1.7372 |
| Pine.Plantation | 186 | 200 | 0.005238 | 2.823341 |
| Sandhill | 186 | 200 | 0.01201 | -2.53774 |
| Sandhill.Upland.Lake | 186 | 200 | 9.95E-07 | 5.054004 |
| Succesional.Hardwood.Forest | 186 | 200 | 0.011343 | 2.555862 |
| Xeric.Hammock | 186 | 200 | 8.64E-08 | 5.561391 |
| Improved | 186 | 200 | 0.144033 | 1.466765 |
| Abandoned.Field.Pasture | 186 | 200 | 0.90615 | 0.118048 |
| Scrubby.Flatwoods | 186 | 200 | 0.065986 | 1.848742 |
| Clastic.Upland.Lake | 186 | 200 | 9.70E-05 | 3.981123 |
| Basin.Marsh | 328 | 300 | 0.807132 | 0.244457 |
| Basin.Swamp | 328 | 300 | 7.89E-06 | -4.59061 |
| Baygall | 328 | 300 | 0.092936 | -1.68826 |
| Lake.Bottom | 328 | 300 | 8.95E-08 | 5.565525 |
| Mesic.Hammock | 328 | 300 | 0.117771 | 1.571425 |
| Pine.Plantation | 328 | 300 | 0.215294 | 1.243134 |
| Sandhill | 328 | 300 | 0.500371 | 0.675332 |
| Sandhill.Upland.Lake | 328 | 300 | 0.005369 | 2.815266 |
| Succesional.Hardwood.Forest | 328 | 300 | 0.021248 | 2.324736 |
| Xeric.Hammock | 328 | 300 | 0.004157 | -2.89981 |
| Improved | 328 | 300 | 9.16E-17 | -9.12476 |
| Abandoned.Field.Pasture | 328 | 300 | 0.014287 | -2.47181 |
| Scrubby.Flatwoods | 328 | 300 | 0.186794 | -1.32473 |
| Clastic.Upland.Lake | 328 | 300 | 0.002214 | -3.10211 |
| Basin.Marsh | 397 | 400 | 0.00429 | -2.88992 |
| Basin.Swamp | 397 | 400 | 0.625227 | 0.489232 |
| Baygall | 397 | 400 | 0.587562 | -0.54326 |
| Lake.Bottom | 397 | 400 | 1.43E-06 | 4.994797 |
| Mesic.Hammock | 397 | 400 | 0.009809 | 2.607759 |
| Pine.Plantation | 397 | 400 | 0.017092 | -2.40952 |
| Sandhill | 397 | 400 | 0.147851 | -1.45364 |
| Sandhill.Upland.Lake | 397 | 400 | 1.84E-14 | 8.282875 |
| Succesional.Hardwood.Forest | 397 | 400 | 0.190346 | 1.314913 |
| Xeric.Hammock | 397 | 400 | 3.59E-10 | -6.60733 |
| Improved | 397 | 400 | 2.65E-05 | -4.30706 |
| Abandoned.Field.Pasture | 397 | 400 | 0.308756 | -1.02055 |
| Scrubby.Flatwoods | 397 | 400 | 0.177254 | 1.354082 |
| Clastic.Upland.Lake | 397 | 400 | 0.00095 | 3.355248 |
| Basin.Marsh | 510 | 500 | 0.897844 | 0.128604 |
| Basin.Swamp | 510 | 500 | 0.005236 | -2.8361 |
| Baygall | 510 | 500 | 0.076353 | 1.782668 |
| Lake.Bottom | 510 | 500 | 0.008443 | -2.6722 |
| Mesic.Hammock | 510 | 500 | 0.077458 | 1.775055 |
| Pine.Plantation | 510 | 500 | 0.688741 | -0.40129 |
| Sandhill | 510 | 500 | 0.805922 | 0.246085 |
| Sandhill.Upland.Lake | 510 | 500 | 3.72E-06 | -4.78781 |
| Succesional.Hardwood.Forest | 510 | 500 | 0.017835 | 2.392266 |
| Xeric.Hammock | 510 | 500 | 0.336169 | 0.965624 |
| Improved | 510 | 500 | 0.00018 | -3.82058 |
| Abandoned.Field.Pasture | 510 | 500 | 0.092565 | -1.69031 |
| Scrubby.Flatwoods | 510 | 500 | 0.007924 | 2.683791 |
| Clastic.Upland.Lake | 510 | 500 | 7.44E-10 | -6.51278 |

Table S9: Results of Student’s t-test comparing the values of PD_SES_ for randomly sampled and targeted subsets for 14 communities (Question 2).

| Community | Subset (targeted) | Subset (random) | P-value | Statistic |
| --- | --- | --- | --- | --- |
| Basin.Marsh | 89 | 100 | 0.07404 | -1.79588 |
| Basin.Swamp | 89 | 100 | 7.43E-14 | -8.21344 |
| Baygall | 89 | 100 | 3.56E-05 | -4.24938 |
| Lake.Bottom | 89 | 100 | 9.59E-07 | 5.074791 |
| Mesic.Hammock | 89 | 100 | 0.001572 | -3.20634 |
| Pine.Plantation | 89 | 100 | 0.565946 | 0.575094 |
| Sandhill | 89 | 100 | 0.093744 | 1.684069 |
| Sandhill.Upland.Lake | 89 | 100 | 0.00017 | 3.832722 |
| Succesional.Hardwood.Forest | 89 | 100 | 0.007292 | 2.711254 |
| Xeric.Hammock | 89 | 100 | 0.00122 | -3.28322 |
| Improved | 89 | 100 | 0.007894 | -2.68472 |
| Abandoned.Field.Pasture | 89 | 100 | 0.051763 | 1.956927 |
| Scrubby.Flatwoods | 89 | 100 | 0.382099 | -0.87623 |
| Clastic.Upland.Lake | 89 | 100 | 1.70E-08 | 5.912325 |
| Basin.Marsh | 186 | 200 | 7.08E-05 | 4.062126 |
| Basin.Swamp | 186 | 200 | 2.37E-08 | 5.85127 |
| Baygall | 186 | 200 | 0.015272 | 2.44741 |
| Lake.Bottom | 186 | 200 | 0.001988 | -3.14199 |
| Mesic.Hammock | 186 | 200 | 0.026738 | 2.234767 |
| Pine.Plantation | 186 | 200 | 0.06576 | 1.850538 |
| Sandhill | 186 | 200 | 0.672107 | -0.42402 |
| Sandhill.Upland.Lake | 186 | 200 | 1.30E-08 | 5.9536 |
| Succesional.Hardwood.Forest | 186 | 200 | 0.291171 | -1.05839 |
| Xeric.Hammock | 186 | 200 | 1.07E-05 | 4.519478 |
| Improved | 186 | 200 | 0.006283 | 2.762155 |
| Abandoned.Field.Pasture | 186 | 200 | 0.263961 | 1.120279 |
| Scrubby.Flatwoods | 186 | 200 | 0.222153 | 1.224677 |
| Clastic.Upland.Lake | 186 | 200 | 0.046881 | 1.999906 |
| Basin.Marsh | 328 | 300 | 0.108519 | -1.61217 |
| Basin.Swamp | 328 | 300 | 2.78E-08 | -5.85626 |
| Baygall | 328 | 300 | 0.001051 | -3.32584 |
| Lake.Bottom | 328 | 300 | 2.33E-09 | 6.331038 |
| Mesic.Hammock | 328 | 300 | 0.791585 | 0.264622 |
| Pine.Plantation | 328 | 300 | 0.320932 | -0.99506 |
| Sandhill | 328 | 300 | 0.887356 | -0.14185 |
| Sandhill.Upland.Lake | 328 | 300 | 0.03072 | 2.176753 |
| Succesional.Hardwood.Forest | 328 | 300 | 9.90E-05 | 3.98227 |
| Xeric.Hammock | 328 | 300 | 0.009998 | -2.601 |
| Improved | 328 | 300 | 6.92E-11 | -6.90102 |
| Abandoned.Field.Pasture | 328 | 300 | 0.000133 | -3.89675 |
| Scrubby.Flatwoods | 328 | 300 | 0.236755 | -1.18694 |
| Clastic.Upland.Lake | 328 | 300 | 2.09E-08 | -5.85511 |
| Basin.Marsh | 397 | 400 | 2.43E-05 | -4.32473 |
| Basin.Swamp | 397 | 400 | 0.684747 | 0.406654 |
| Baygall | 397 | 400 | 0.754749 | -0.31282 |
| Lake.Bottom | 397 | 400 | 1.33E-10 | 6.883356 |
| Mesic.Hammock | 397 | 400 | 0.00331 | 2.974263 |
| Pine.Plantation | 397 | 400 | 0.820779 | 0.226856 |
| Sandhill | 397 | 400 | 0.022183 | 2.308096 |
| Sandhill.Upland.Lake | 397 | 400 | 7.33E-06 | 4.612947 |
| Succesional.Hardwood.Forest | 397 | 400 | 0.209457 | 1.259216 |
| Xeric.Hammock | 397 | 400 | 4.18E-07 | -5.24067 |
| Improved | 397 | 400 | 0.503167 | -0.67082 |
| Abandoned.Field.Pasture | 397 | 400 | 0.302832 | -1.03308 |
| Scrubby.Flatwoods | 397 | 400 | 0.076133 | 1.783101 |
| Clastic.Upland.Lake | 397 | 400 | 0.004643 | 2.863706 |
| Basin.Marsh | 510 | 500 | 0.352324 | 0.933188 |
| Basin.Swamp | 510 | 500 | 1.09E-14 | -8.45966 |
| Baygall | 510 | 500 | 0.203889 | 1.277307 |
| Lake.Bottom | 510 | 500 | 0.841579 | 0.200236 |
| Mesic.Hammock | 510 | 500 | 0.427093 | 0.795969 |
| Pine.Plantation | 510 | 500 | 0.092554 | 1.690256 |
| Sandhill | 510 | 500 | 0.740879 | -0.3312 |
| Sandhill.Upland.Lake | 510 | 500 | 0.190671 | -1.31323 |
| Succesional.Hardwood.Forest | 510 | 500 | 5.53E-05 | 4.144081 |
| Xeric.Hammock | 510 | 500 | 0.007886 | 2.699982 |
| Improved | 510 | 500 | 0.000436 | -3.57935 |
| Abandoned.Field.Pasture | 510 | 500 | 4.83E-06 | -4.70949 |
| Scrubby.Flatwoods | 510 | 500 | 0.162139 | 1.403168 |
| Clastic.Upland.Lake | 510 | 500 | 3.79E-14 | -8.18016 |

Table S10: Numbers and proportions of replicates showing significant phylogenetic patterns of NRI for targeted subsets for each community (Question 2). Results are separated into clustered and overdispersed patterns.

| Community | Subset | Number Overdispersed | Proportion Overdispersed | Number Clustered | Proportion Clustered |
| --- | --- | --- | --- | --- | --- |
| Basin.Marsh | 89 | 4 | 0.04 | 15 | 0.15 |
| Basin.Swamp | 89 | 0 | 0 | 6 | 0.06 |
| Baygall | 89 | 0 | 0 | 31 | 0.31 |
| Lake.Bottom | 89 | 19 | 0.19 | 2 | 0.02 |
| Mesic.Hammock | 89 | 0 | 0 | 30 | 0.3 |
| Pine.Plantation | 89 | 9 | 0.09 | 4 | 0.04 |
| Sandhill | 89 | 0 | 0 | 16 | 0.16 |
| Sandhill.Upland.Lake | 89 | 22 | 0.22 | 0 | 0 |
| Succesional.Hardwood.Forest | 89 | 4 | 0.04 | 3 | 0.03 |
| Xeric.Hammock | 89 | 0 | 0 | 26 | 0.26 |
| Improved | 89 | 0 | 0 | 1 | 0.01 |
| Abandoned.Field.Pasture | 89 | 0 | 0 | 18 | 0.18 |
| Scrubby.Flatwoods | 89 | 5 | 0.05 | 14 | 0.14 |
| Clastic.Upland.Lake | 89 | 0 | 0 | 1 | 0.01 |
| Basin.Marsh | 186 | 0 | 0 | 8 | 0.08 |
| Basin.Swamp | 186 | 58 | 0.58 | 0 | 0 |
| Baygall | 186 | 0 | 0 | 39 | 0.39 |
| Lake.Bottom | 186 | 0 | 0 | 1 | 0.01 |
| Mesic.Hammock | 186 | 6 | 0.06 | 0 | 0 |
| Pine.Plantation | 186 | 0 | 0 | 12 | 0.12 |
| Sandhill | 186 | 0 | 0 | 41 | 0.41 |
| Sandhill.Upland.Lake | 186 | 46 | 0.46 | 0 | 0 |
| Succesional.Hardwood.Forest | 186 | 0 | 0 | 9 | 0.09 |
| Xeric.Hammock | 186 | 0 | 0 | 34 | 0.34 |
| Improved | 186 | 0 | 0 | 0 | 0 |
| Abandoned.Field.Pasture | 186 | 0 | 0 | 10 | 0.1 |
| Scrubby.Flatwoods | 186 | 0 | 0 | 26 | 0.26 |
| Clastic.Upland.Lake | 186 | 0 | 0 | 24 | 0.24 |
| Basin.Marsh | 328 | 0 | 0 | 24 | 0.24 |
| Basin.Swamp | 328 | 37 | 0.37 | 0 | 0 |
| Baygall | 328 | 0 | 0 | 60 | 0.6 |
| Lake.Bottom | 328 | 0 | 0 | 0 | 0 |
| Mesic.Hammock | 328 | 0 | 0 | 0 | 0 |
| Pine.Plantation | 328 | 0 | 0 | 10 | 0.1 |
| Sandhill | 328 | 0 | 0 | 74 | 0.74 |
| Sandhill.Upland.Lake | 328 | 3 | 0.03 | 0 | 0 |
| Succesional.Hardwood.Forest | 328 | 7 | 0.07 | 0 | 0 |
| Xeric.Hammock | 328 | 0 | 0 | 51 | 0.51 |
| Improved | 328 | 0 | 0 | 9 | 0.09 |
| Abandoned.Field.Pasture | 328 | 0 | 0 | 30 | 0.3 |
| Scrubby.Flatwoods | 328 | 0 | 0 | 50 | 0.5 |
| Clastic.Upland.Lake | 328 | 0 | 0 | 61 | 0.61 |
| Basin.Marsh | 397 | 0 | 0 | 26 | 0.26 |
| Basin.Swamp | 397 | 39 | 0.39 | 0 | 0 |
| Baygall | 397 | 0 | 0 | 62 | 0.62 |
| Lake.Bottom | 397 | 0 | 0 | 0 | 0 |
| Mesic.Hammock | 397 | 0 | 0 | 0 | 0 |
| Pine.Plantation | 397 | 0 | 0 | 3 | 0.03 |
| Sandhill | 397 | 0 | 0 | 93 | 0.93 |
| Sandhill.Upland.Lake | 397 | 8 | 0.08 | 0 | 0 |
| Succesional.Hardwood.Forest | 397 | 9 | 0.09 | 0 | 0 |
| Xeric.Hammock | 397 | 0 | 0 | 58 | 0.58 |
| Improved | 397 | 0 | 0 | 1 | 0.01 |
| Abandoned.Field.Pasture | 397 | 0 | 0 | 30 | 0.3 |
| Scrubby.Flatwoods | 397 | 0 | 0 | 52 | 0.52 |
| Clastic.Upland.Lake | 397 | 0 | 0 | 64 | 0.64 |
| Basin.Marsh | 510 | 0 | 0 | 0 | 0 |
| Basin.Swamp | 510 | 100 | 1 | 0 | 0 |
| Baygall | 510 | 0 | 0 | 95 | 0.95 |
| Lake.Bottom | 510 | 0 | 0 | 0 | 0 |
| Mesic.Hammock | 510 | 0 | 0 | 0 | 0 |
| Pine.Plantation | 510 | 0 | 0 | 3 | 0.03 |
| Sandhill | 510 | 0 | 0 | 100 | 1 |
| Sandhill.Upland.Lake | 510 | 33 | 0.33 | 0 | 0 |
| Succesional.Hardwood.Forest | 510 | 0 | 0 | 0 | 0 |
| Xeric.Hammock | 510 | 0 | 0 | 75 | 0.75 |
| Improved | 510 | 0 | 0 | 0 | 0 |
| Abandoned.Field.Pasture | 510 | 0 | 0 | 2 | 0.02 |
| Scrubby.Flatwoods | 510 | 0 | 0 | 83 | 0.83 |
| Clastic.Upland.Lake | 510 | 0 | 0 | 100 | 1 |

Table S11: Numbers and proportions of replicates showing significant phylogenetic patterns of NTI for targeted subsets for each community (Question 2). Results are separated into clustered and overdispersed patterns.

| Community | Subset | Number Overdispersed | Proportion Overdispersed | Number Clustered | Proportion Clustered |
| --- | --- | --- | --- | --- | --- |
| Basin.Marsh | 89 | 2 | 0.02 | 2 | 0.02 |
| Basin.Swamp | 89 | 0 | 0 | 1 | 0.01 |
| Baygall | 89 | 0 | 0 | 3 | 0.03 |
| Lake.Bottom | 89 | 10 | 0.1 | 1 | 0.01 |
| Mesic.Hammock | 89 | 6 | 0.06 | 2 | 0.02 |
| Pine.Plantation | 89 | 6 | 0.06 | 3 | 0.03 |
| Sandhill | 89 | 0 | 0 | 28 | 0.28 |
| Sandhill.Upland.Lake | 89 | 26 | 0.26 | 0 | 0 |
| Succesional.Hardwood.Forest | 89 | 5 | 0.05 | 1 | 0.01 |
| Xeric.Hammock | 89 | 0 | 0 | 19 | 0.19 |
| Improved | 89 | 0 | 0 | 29 | 0.29 |
| Abandoned.Field.Pasture | 89 | 3 | 0.03 | 0 | 0 |
| Scrubby.Flatwoods | 89 | 3 | 0.03 | 16 | 0.16 |
| Clastic.Upland.Lake | 89 | 2 | 0.02 | 1 | 0.01 |
| Basin.Marsh | 186 | 18 | 0.18 | 2 | 0.02 |
| Basin.Swamp | 186 | 11 | 0.11 | 0 | 0 |
| Baygall | 186 | 0 | 0 | 17 | 0.17 |
| Lake.Bottom | 186 | 0 | 0 | 19 | 0.19 |
| Mesic.Hammock | 186 | 0 | 0 | 3 | 0.03 |
| Pine.Plantation | 186 | 2 | 0.02 | 11 | 0.11 |
| Sandhill | 186 | 0 | 0 | 93 | 0.93 |
| Sandhill.Upland.Lake | 186 | 18 | 0.18 | 0 | 0 |
| Succesional.Hardwood.Forest | 186 | 13 | 0.13 | 0 | 0 |
| Xeric.Hammock | 186 | 0 | 0 | 22 | 0.22 |
| Improved | 186 | 0 | 0 | 26 | 0.26 |
| Abandoned.Field.Pasture | 186 | 0 | 0 | 3 | 0.03 |
| Scrubby.Flatwoods | 186 | 0 | 0 | 39 | 0.39 |
| Clastic.Upland.Lake | 186 | 0 | 0 | 1 | 0.01 |
| Basin.Marsh | 328 | 11 | 0.11 | 0 | 0 |
| Basin.Swamp | 328 | 0 | 0 | 6 | 0.06 |
| Baygall | 328 | 0 | 0 | 29 | 0.29 |
| Lake.Bottom | 328 | 0 | 0 | 9 | 0.09 |
| Mesic.Hammock | 328 | 0 | 0 | 6 | 0.06 |
| Pine.Plantation | 328 | 0 | 0 | 38 | 0.38 |
| Sandhill | 328 | 0 | 0 | 96 | 0.96 |
| Sandhill.Upland.Lake | 328 | 0 | 0 | 12 | 0.12 |
| Succesional.Hardwood.Forest | 328 | 1 | 0.01 | 0 | 0 |
| Xeric.Hammock | 328 | 0 | 0 | 57 | 0.57 |
| Improved | 328 | 0 | 0 | 92 | 0.92 |
| Abandoned.Field.Pasture | 328 | 0 | 0 | 5 | 0.05 |
| Scrubby.Flatwoods | 328 | 0 | 0 | 82 | 0.82 |
| Clastic.Upland.Lake | 328 | 0 | 0 | 5 | 0.05 |
| Basin.Marsh | 397 | 4 | 0.04 | 0 | 0 |
| Basin.Swamp | 397 | 0 | 0 | 2 | 0.02 |
| Baygall | 397 | 0 | 0 | 34 | 0.34 |
| Lake.Bottom | 397 | 0 | 0 | 17 | 0.17 |
| Mesic.Hammock | 397 | 0 | 0 | 7 | 0.07 |
| Pine.Plantation | 397 | 0 | 0 | 87 | 0.87 |
| Sandhill | 397 | 0 | 0 | 100 | 1 |
| Sandhill.Upland.Lake | 397 | 0 | 0 | 17 | 0.17 |
| Succesional.Hardwood.Forest | 397 | 0 | 0 | 0 | 0 |
| Xeric.Hammock | 397 | 0 | 0 | 74 | 0.74 |
| Improved | 397 | 0 | 0 | 93 | 0.93 |
| Abandoned.Field.Pasture | 397 | 0 | 0 | 5 | 0.05 |
| Scrubby.Flatwoods | 397 | 0 | 0 | 96 | 0.96 |
| Clastic.Upland.Lake | 397 | 0 | 0 | 2 | 0.02 |
| Basin.Marsh | 510 | 0 | 0 | 0 | 0 |
| Basin.Swamp | 510 | 100 | 1 | 0 | 0 |
| Baygall | 510 | 0 | 0 | 95 | 0.95 |
| Lake.Bottom | 510 | 0 | 0 | 0 | 0 |
| Mesic.Hammock | 510 | 0 | 0 | 0 | 0 |
| Pine.Plantation | 510 | 0 | 0 | 3 | 0.03 |
| Sandhill | 510 | 0 | 0 | 100 | 1 |
| Sandhill.Upland.Lake | 510 | 33 | 0.33 | 0 | 0 |
| Succesional.Hardwood.Forest | 510 | 0 | 0 | 0 | 0 |
| Xeric.Hammock | 510 | 0 | 0 | 75 | 0.75 |
| Improved | 510 | 0 | 0 | 0 | 0 |
| Abandoned.Field.Pasture | 510 | 0 | 0 | 2 | 0.02 |
| Scrubby.Flatwoods | 510 | 0 | 0 | 83 | 0.83 |
| Clastic.Upland.Lake | 510 | 0 | 0 | 100 | 1 |

Table S12: Numbers and proportions of replicates showing significant phylogenetic patterns of PD_SES_ for targeted subsets for each community (Question 2). Results are separated into clustered and overdispersed patterns.

| Community | Subset | Number Overdispersed | Proportion Overdispersed | Number Clustered | Proportion Clustered |
| --- | --- | --- | --- | --- | --- |
| Basin.Marsh | 89 | 7 | 0.07 | 3 | 0.03 |
| Basin.Swamp | 89 | 3 | 0.03 | 0 | 0 |
| Baygall | 89 | 0 | 0 | 5 | 0.05 |
| Lake.Bottom | 89 | 21 | 0.21 | 1 | 0.01 |
| Mesic.Hammock | 89 | 3 | 0.03 | 1 | 0.01 |
| Pine.Plantation | 89 | 12 | 0.12 | 1 | 0.01 |
| Sandhill | 89 | 0 | 0 | 10 | 0.1 |
| Sandhill.Upland.Lake | 89 | 46 | 0.46 | 0 | 0 |
| Succesional.Hardwood.Forest | 89 | 11 | 0.11 | 0 | 0 |
| Xeric.Hammock | 89 | 0 | 0 | 14 | 0.14 |
| Improved | 89 | 0 | 0 | 7 | 0.07 |
| Abandoned.Field.Pasture | 89 | 1 | 0.01 | 1 | 0.01 |
| Scrubby.Flatwoods | 89 | 5 | 0.05 | 6 | 0.06 |
| Clastic.Upland.Lake | 89 | 14 | 0.14 | 0 | 0 |
| Basin.Marsh | 186 | 19 | 0.19 | 0 | 0 |
| Basin.Swamp | 186 | 17 | 0.17 | 0 | 0 |
| Baygall | 186 | 1 | 0.01 | 3 | 0.03 |
| Lake.Bottom | 186 | 0 | 0 | 0 | 0 |
| Mesic.Hammock | 186 | 1 | 0.01 | 0 | 0 |
| Pine.Plantation | 186 | 4 | 0.04 | 1 | 0.01 |
| Sandhill | 186 | 0 | 0 | 60 | 0.6 |
| Sandhill.Upland.Lake | 186 | 53 | 0.53 | 0 | 0 |
| Succesional.Hardwood.Forest | 186 | 12 | 0.12 | 0 | 0 |
| Xeric.Hammock | 186 | 0 | 0 | 4 | 0.04 |
| Improved | 186 | 0 | 0 | 1 | 0.01 |
| Abandoned.Field.Pasture | 186 | 0 | 0 | 0 | 0 |
| Scrubby.Flatwoods | 186 | 0 | 0 | 4 | 0.04 |
| Clastic.Upland.Lake | 186 | 0 | 0 | 0 | 0 |
| Basin.Marsh | 328 | 8 | 0.08 | 0 | 0 |
| Basin.Swamp | 328 | 0 | 0 | 0 | 0 |
| Baygall | 328 | 0 | 0 | 10 | 0.1 |
| Lake.Bottom | 328 | 0 | 0 | 0 | 0 |
| Mesic.Hammock | 328 | 1 | 0.01 | 0 | 0 |
| Pine.Plantation | 328 | 0 | 0 | 0 | 0 |
| Sandhill | 328 | 0 | 0 | 98 | 0.98 |
| Sandhill.Upland.Lake | 328 | 17 | 0.17 | 0 | 0 |
| Succesional.Hardwood.Forest | 328 | 23 | 0.23 | 0 | 0 |
| Xeric.Hammock | 328 | 0 | 0 | 34 | 0.34 |
| Improved | 328 | 0 | 0 | 51 | 0.51 |
| Abandoned.Field.Pasture | 328 | 0 | 0 | 0 | 0 |
| Scrubby.Flatwoods | 328 | 0 | 0 | 9 | 0.09 |
| Clastic.Upland.Lake | 328 | 0 | 0 | 0 | 0 |
| Basin.Marsh | 397 | 4 | 0.04 | 0 | 0 |
| Basin.Swamp | 397 | 0 | 0 | 0 | 0 |
| Baygall | 397 | 0 | 0 | 4 | 0.04 |
| Lake.Bottom | 397 | 0 | 0 | 0 | 0 |
| Mesic.Hammock | 397 | 0 | 0 | 0 | 0 |
| Pine.Plantation | 397 | 0 | 0 | 0 | 0 |
| Sandhill | 397 | 0 | 0 | 100 | 1 |
| Sandhill.Upland.Lake | 397 | 2 | 0.02 | 0 | 0 |
| Succesional.Hardwood.Forest | 397 | 9 | 0.09 | 0 | 0 |
| Xeric.Hammock | 397 | 0 | 0 | 51 | 0.51 |
| Improved | 397 | 0 | 0 | 61 | 0.61 |
| Abandoned.Field.Pasture | 397 | 0 | 0 | 0 | 0 |
| Scrubby.Flatwoods | 397 | 0 | 0 | 41 | 0.41 |
| Clastic.Upland.Lake | 397 | 0 | 0 | 0 | 0 |
| Basin.Marsh | 510 | 0 | 0 | 0 | 0 |
| Basin.Swamp | 510 | 0 | 0 | 0 | 0 |
| Baygall | 510 | 0 | 0 | 0 | 0 |
| Lake.Bottom | 510 | 0 | 0 | 0 | 0 |
| Mesic.Hammock | 510 | 0 | 0 | 0 | 0 |
| Pine.Plantation | 510 | 0 | 0 | 0 | 0 |
| Sandhill | 510 | 0 | 0 | 100 | 1 |
| Sandhill.Upland.Lake | 510 | 0 | 0 | 0 | 0 |
| Succesional.Hardwood.Forest | 510 | 0 | 0 | 0 | 0 |
| Xeric.Hammock | 510 | 0 | 0 | 2 | 0.02 |
| Improved | 510 | 0 | 0 | 98 | 0.98 |
| Abandoned.Field.Pasture | 510 | 0 | 0 | 0 | 0 |
| Scrubby.Flatwoods | 510 | 0 | 0 | 100 | 1 |
| Clastic.Upland.Lake | 510 | 0 | 0 | 0 | 0 |

Table S13: Numbers of taxa in targeted datasets for each community (average, maximum, minimum and standard deviation) (Question 2).

| Community | Subset | Average | Maximum | Minimum | Standard deviation |
| --- | --- | --- | --- | --- | --- |
| Abandoned.Field.Pasture | 89 | 10.96 | 16 | 5 | 2.498161 |
| Basin.Marsh | 89 | 7.27 | 14 | 2 | 2.381982 |
| Basin.Swamp | 89 | 3.16 | 7 | 0 | 1.502321 |
| Baygall | 89 | 5.41 | 9 | 1 | 1.826267 |
| Clastic.Upland.Lake | 89 | 3.41 | 8 | 0 | 1.787131 |
| Improved | 89 | 11.26 | 19 | 5 | 2.691626 |
| Lake.Bottom | 89 | 13.9 | 22 | 8 | 2.779797 |
| Mesic.Hammock | 89 | 20.46 | 34 | 12 | 3.67745 |
| Pine.Plantation | 89 | 3.76 | 7 | 0 | 1.295836 |
| Sandhill | 89 | 34.32 | 43 | 24 | 4.004745 |
| Sandhill.Upland.Lake | 89 | 13.34 | 20 | 8 | 2.625516 |
| Scrubby.Flatwoods | 89 | 4.55 | 8 | 0 | 1.641476 |
| Succesional.Hardwood.Forest | 89 | 18.78 | 27 | 12 | 2.939044 |
| Xeric.Hammock | 89 | 7.73 | 12 | 4 | 1.824939 |
| Abandoned.Field.Pasture | 186 | 21.83 | 30 | 15 | 3.216294 |
| Basin.Marsh | 186 | 13.22 | 19 | 7 | 2.504864 |
| Basin.Swamp | 186 | 8.72 | 13 | 3 | 2.216012 |
| Baygall | 186 | 9.43 | 14 | 4 | 2.266377 |
| Clastic.Upland.Lake | 186 | 8.27 | 14 | 4 | 2.308767 |
| Improved | 186 | 25.94 | 35 | 16 | 3.727647 |
| Lake.Bottom | 186 | 21.76 | 35 | 15 | 3.598877 |
| Mesic.Hammock | 186 | 43.12 | 55 | 31 | 5.025752 |
| Pine.Plantation | 186 | 5.63 | 10 | 2 | 1.732955 |
| Sandhill | 186 | 68.09 | 76 | 58 | 3.992657 |
| Sandhill.Upland.Lake | 186 | 25.3 | 37 | 15 | 3.854684 |
| Scrubby.Flatwoods | 186 | 8 | 14 | 4 | 2.291839 |
| Succesional.Hardwood.Forest | 186 | 42.94 | 55 | 33 | 4.672767 |
| Xeric.Hammock | 186 | 13.47 | 18 | 9 | 2.047196 |
| Abandoned.Field.Pasture | 328 | 40.69 | 49 | 33 | 3.70829 |
| Basin.Marsh | 328 | 27.12 | 35 | 22 | 2.789881 |
| Basin.Swamp | 328 | 16.77 | 24 | 12 | 2.381982 |
| Baygall | 328 | 20.73 | 26 | 13 | 2.330539 |
| Clastic.Upland.Lake | 328 | 17.47 | 23 | 12 | 2.213161 |
| Improved | 328 | 50.58 | 58 | 43 | 3.651982 |
| Lake.Bottom | 328 | 42.95 | 50 | 30 | 3.488437 |
| Mesic.Hammock | 328 | 79.89 | 91 | 67 | 4.324922 |
| Pine.Plantation | 328 | 12.05 | 16 | 5 | 1.903612 |
| Sandhill | 328 | 112.7 | 128 | 98 | 5.547772 |
| Sandhill.Upland.Lake | 328 | 45.11 | 54 | 36 | 3.697925 |
| Scrubby.Flatwoods | 328 | 14.83 | 21 | 10 | 2.335735 |
| Succesional.Hardwood.Forest | 328 | 75.76 | 88 | 67 | 4.579533 |
| Xeric.Hammock | 328 | 24.63 | 31 | 19 | 2.545168 |
| Abandoned.Field.Pasture | 397 | 48.87 | 59 | 41 | 3.520947 |
| Basin.Marsh | 397 | 31.67 | 38 | 25 | 2.663085 |
| Basin.Swamp | 397 | 18.65 | 24 | 13 | 2.147938 |
| Baygall | 397 | 23.36 | 29 | 19 | 2.171998 |
| Clastic.Upland.Lake | 397 | 19.22 | 24 | 13 | 2.0869 |
| Improved | 397 | 60.06 | 69 | 52 | 3.329756 |
| Lake.Bottom | 397 | 52.69 | 61 | 45 | 3.183591 |
| Mesic.Hammock | 397 | 93.33 | 104 | 83 | 4.238007 |
| Pine.Plantation | 397 | 14.99 | 19 | 10 | 1.789391 |
| Sandhill | 397 | 143.06 | 156 | 133 | 5.048892 |
| Sandhill.Upland.Lake | 397 | 53.97 | 64 | 47 | 3.292032 |
| Scrubby.Flatwoods | 397 | 19.56 | 24 | 14 | 2.056377 |
| Succesional.Hardwood.Forest | 397 | 90.77 | 108 | 82 | 4.032331 |
| Xeric.Hammock | 397 | 30.6 | 37 | 23 | 2.251823 |
| Abandoned.Field.Pasture | 510 | 62.32 | 68 | 56 | 2.304497 |
| Basin.Marsh | 510 | 39.69 | 43 | 34 | 1.715615 |
| Basin.Swamp | 510 | 25.46 | 27 | 21 | 1.149616 |
| Baygall | 510 | 29.95 | 33 | 26 | 1.290016 |
| Clastic.Upland.Lake | 510 | 26.27 | 28 | 22 | 1.285938 |
| Improved | 510 | 76.03 | 82 | 70 | 2.455482 |
| Lake.Bottom | 510 | 64.46 | 70 | 57 | 2.422204 |
| Mesic.Hammock | 510 | 121.58 | 127 | 114 | 2.745722 |
| Pine.Plantation | 510 | 17.29 | 20 | 14 | 1.121822 |
| Sandhill | 510 | 178.88 | 187 | 171 | 3.618918 |
| Sandhill.Upland.Lake | 510 | 70.33 | 75 | 63 | 2.132268 |
| Scrubby.Flatwoods | 510 | 23.33 | 27 | 20 | 1.497844 |
| Succesional.Hardwood.Forest | 510 | 116.62 | 123 | 109 | 2.722076 |
| Xeric.Hammock | 510 | 37.41 | 41 | 32 | 1.944144 |

Table S14: Results of Student’s t-test comparing the values of NRI for reconstructed and pruned phylogenies for randomly sampled subsets for 14 communities (Question 4).

| Subset | Community | P-value | Statistic |
| --- | --- | --- | --- |
| 100 | Basin.Marsh | 0.894152 | -0.13447 |
| 100 | Basin.Swamp | 0.458214 | -0.75449 |
| 100 | Baygall | 0.506432 | 0.674511 |
| 100 | Lake.Bottom | 0.654214 | 0.453569 |
| 100 | Mesic.Hammock | 0.334061 | -0.98581 |
| 100 | Pine.Plantation | 0.877917 | -0.15559 |
| 100 | Sandhill | 0.788443 | -0.27135 |
| 100 | Sandhill.Upland.Lake | 0.928635 | -0.09051 |
| 100 | Succesional.Hardwood.Forest | 0.781731 | -0.28019 |
| 100 | Xeric.Hammock | 0.353512 | -0.94613 |
| 100 | Improved | 0.448055 | -0.7713 |
| 100 | Abandoned.Field.Pasture | 0.783042 | 0.278463 |
| 100 | Scrubby.Flatwoods | 0.786074 | 0.274467 |
| 100 | Clastic.Upland.Lake | 0.964325 | 0.045217 |
| 200 | Basin.Marsh | 0.267408 | -1.11546 |
| 200 | Basin.Swamp | 0.001982 | -3.17926 |
| 200 | Baygall | 0.046694 | -2.01479 |
| 200 | Lake.Bottom | 0.044837 | 2.032492 |
| 200 | Mesic.Hammock | 0.000169 | -3.91264 |
| 200 | Pine.Plantation | 0.896027 | 0.131025 |
| 200 | Sandhill | 0.243787 | -1.1727 |
| 200 | Sandhill.Upland.Lake | 0.571271 | -0.56811 |
| 200 | Succesional.Hardwood.Forest | 0.484676 | -0.70149 |
| 200 | Xeric.Hammock | 0.016385 | -2.44274 |
| 200 | Improved | 0.346954 | 0.945105 |
| 200 | Abandoned.Field.Pasture | 0.247681 | -1.163 |
| 200 | Scrubby.Flatwoods | 0.803708 | -0.24923 |
| 200 | Clastic.Upland.Lake | 0.514321 | 0.654527 |
| 300 | Basin.Marsh | 0.844626 | -0.19781 |
| 300 | Basin.Swamp | 0.676856 | -0.42116 |
| 300 | Baygall | 0.548229 | -0.60778 |
| 300 | Lake.Bottom | 0.69332 | 0.398452 |
| 300 | Mesic.Hammock | 0.573683 | -0.56931 |
| 300 | Pine.Plantation | 0.848273 | -0.1931 |
| 300 | Sandhill | 0.74582 | -0.32737 |
| 300 | Sandhill.Upland.Lake | 0.363421 | -0.92392 |
| 300 | Succesional.Hardwood.Forest | 0.949662 | 0.0637 |
| 300 | Xeric.Hammock | 0.585567 | -0.55165 |
| 300 | Improved | 0.869194 | 0.1662 |
| 300 | Abandoned.Field.Pasture | 0.765722 | -0.30089 |
| 300 | Scrubby.Flatwoods | 0.639867 | -0.47302 |
| 300 | Clastic.Upland.Lake | 0.933848 | -0.08375 |
| 400 | Basin.Marsh | 1.99E-07 | -5.59181 |
| 400 | Basin.Swamp | 5.50E-12 | -7.83135 |
| 400 | Baygall | 2.72E-09 | -6.53959 |
| 400 | Lake.Bottom | 4.17E-11 | 7.416456 |
| 400 | Mesic.Hammock | 2.42E-16 | -9.842 |
| 400 | Pine.Plantation | 0.990283 | -0.01221 |
| 400 | Sandhill | 0.000518 | -3.58925 |
| 400 | Sandhill.Upland.Lake | 0.006606 | -2.77469 |
| 400 | Succesional.Hardwood.Forest | 0.009084 | -2.66129 |
| 400 | Xeric.Hammock | 1.31E-13 | -8.58663 |
| 400 | Improved | 0.00195 | 3.18264 |
| 400 | Abandoned.Field.Pasture | 0.014977 | -2.47615 |
| 400 | Scrubby.Flatwoods | 0.012373 | -2.54797 |
| 400 | Clastic.Upland.Lake | 0.001551 | -3.25532 |
| 500 | Basin.Marsh | 0.753554 | 0.315687 |
| 500 | Basin.Swamp | 0.141185 | -1.49506 |
| 500 | Baygall | 0.209346 | -1.27175 |
| 500 | Lake.Bottom | 0.592401 | 0.538821 |
| 500 | Mesic.Hammock | 0.019492 | -2.41378 |
| 500 | Pine.Plantation | 0.77796 | 0.283506 |
| 500 | Sandhill | 0.817119 | 0.232474 |
| 500 | Sandhill.Upland.Lake | 0.596262 | -0.53319 |
| 500 | Succesional.Hardwood.Forest | 0.479979 | 0.711665 |
| 500 | Xeric.Hammock | 0.570643 | -0.57087 |
| 500 | Improved | 0.230173 | 1.214735 |
| 500 | Abandoned.Field.Pasture | 0.282174 | -1.08717 |
| 500 | Scrubby.Flatwoods | 0.764281 | -0.3015 |
| 500 | Clastic.Upland.Lake | 0.804749 | -0.24852 |

Table S15: Results of Student’s t-test comparing the values of NTI for reconstructed and pruned phylogenies for randomly sampled subsets for 14 communities (Question 4).

| Subset | Community | P-value | Statistic |
| --- | --- | --- | --- |
| 100 | Basin.Marsh | 0.567449 | -0.57981 |
| 100 | Basin.Swamp | 0.686028 | -0.40941 |
| 100 | Baygall | 0.69028 | 0.403325 |
| 100 | Lake.Bottom | 0.71706 | -0.36669 |
| 100 | Mesic.Hammock | 0.61058 | -0.51599 |
| 100 | Pine.Plantation | 0.429509 | -0.80638 |
| 100 | Sandhill | 0.687513 | -0.40714 |
| 100 | Sandhill.Upland.Lake | 0.571673 | -0.57345 |
| 100 | Succesional.Hardwood.Forest | 0.206944 | -1.29705 |
| 100 | Xeric.Hammock | 0.217253 | -1.26717 |
| 100 | Improved | 0.610951 | -0.51546 |
| 100 | Abandoned.Field.Pasture | 0.790612 | 0.268496 |
| 100 | Scrubby.Flatwoods | 0.852977 | 0.18733 |
| 100 | Clastic.Upland.Lake | 0.895368 | -0.13298 |
| 200 | Basin.Marsh | 0.883525 | -0.14689 |
| 200 | Basin.Swamp | 0.007562 | -2.72814 |
| 200 | Baygall | 0.10801 | -1.62218 |
| 200 | Lake.Bottom | 0.099171 | -1.66484 |
| 200 | Mesic.Hammock | 0.114223 | -1.59385 |
| 200 | Pine.Plantation | 0.55058 | -0.59898 |
| 200 | Sandhill | 0.105797 | -1.63259 |
| 200 | Sandhill.Upland.Lake | 0.063343 | -1.8783 |
| 200 | Succesional.Hardwood.Forest | 0.104765 | -1.63751 |
| 200 | Xeric.Hammock | 0.056837 | -1.92755 |
| 200 | Improved | 0.477649 | 0.712851 |
| 200 | Abandoned.Field.Pasture | 0.249978 | -1.15733 |
| 200 | Scrubby.Flatwoods | 0.589325 | -0.54162 |
| 200 | Clastic.Upland.Lake | 0.071611 | 1.821515 |
| 300 | Basin.Marsh | 0.951886 | 0.060881 |
| 300 | Basin.Swamp | 0.981758 | -0.02307 |
| 300 | Baygall | 0.630341 | -0.48658 |
| 300 | Lake.Bottom | 0.797397 | -0.25917 |
| 300 | Mesic.Hammock | 0.958723 | 0.052221 |
| 300 | Pine.Plantation | 0.815015 | 0.236176 |
| 300 | Sandhill | 0.93309 | -0.08472 |
| 300 | Sandhill.Upland.Lake | 0.936595 | -0.08027 |
| 300 | Succesional.Hardwood.Forest | 0.771856 | -0.29277 |
| 300 | Xeric.Hammock | 0.822725 | -0.22615 |
| 300 | Improved | 0.985177 | -0.01875 |
| 300 | Abandoned.Field.Pasture | 0.855836 | -0.18336 |
| 300 | Scrubby.Flatwoods | 0.708162 | -0.37816 |
| 300 | Clastic.Upland.Lake | 0.779226 | 0.283043 |
| 400 | Basin.Marsh | 0.000314 | -3.7348 |
| 400 | Basin.Swamp | 0.000104 | 4.043761 |
| 400 | Baygall | 1.73E-07 | -5.62464 |
| 400 | Lake.Bottom | 8.78E-06 | -4.68935 |
| 400 | Mesic.Hammock | 0.166509 | 1.393765 |
| 400 | Pine.Plantation | 0.01985 | -2.36756 |
| 400 | Sandhill | 0.012251 | -2.55165 |
| 400 | Sandhill.Upland.Lake | 0.168562 | -1.38698 |
| 400 | Succesional.Hardwood.Forest | 1.25E-15 | -9.5149 |
| 400 | Xeric.Hammock | 1.70E-12 | -8.06971 |
| 400 | Improved | 0.000242 | 3.809587 |
| 400 | Abandoned.Field.Pasture | 0.248259 | -1.16143 |
| 400 | Scrubby.Flatwoods | 2.64E-06 | -4.9848 |
| 400 | Clastic.Upland.Lake | 0.000265 | 3.783289 |
| 500 | Basin.Marsh | 0.767266 | 0.297567 |
| 500 | Basin.Swamp | 0.299067 | 1.04934 |
| 500 | Baygall | 0.210871 | -1.26743 |
| 500 | Lake.Bottom | 0.116232 | -1.59851 |
| 500 | Mesic.Hammock | 0.837908 | 0.205639 |
| 500 | Pine.Plantation | 0.894897 | -0.13278 |
| 500 | Sandhill | 0.123615 | -1.56618 |
| 500 | Sandhill.Upland.Lake | 0.837272 | -0.20646 |
| 500 | Succesional.Hardwood.Forest | 0.161963 | -1.41948 |
| 500 | Xeric.Hammock | 0.72464 | -0.35425 |
| 500 | Improved | 0.550528 | 0.601041 |
| 500 | Abandoned.Field.Pasture | 0.946118 | -0.06792 |
| 500 | Scrubby.Flatwoods | 0.740513 | 0.333018 |
| 500 | Clastic.Upland.Lake | 0.979602 | 0.025696 |

Table S16: Results of Student’s t-test comparing the values of PD_SES_ for reconstructed and pruned phylogenies for randomly sampled subsets for 14 communities (Question 4).

| Subset | Community | P-value | Statistic |
| --- | --- | --- | --- |
| 100 | Basin.Marsh | 0.208158 | 1.293475 |
| 100 | Basin.Swamp | 0.920401 | 0.101031 |
| 100 | Baygall | 0.08449 | 1.799723 |
| 100 | Lake.Bottom | 0.511122 | 0.66702 |
| 100 | Mesic.Hammock | 0.368773 | -0.91601 |
| 100 | Pine.Plantation | 0.312104 | 1.037018 |
| 100 | Sandhill | 0.867379 | -0.16879 |
| 100 | Sandhill.Upland.Lake | 0.626878 | 0.492447 |
| 100 | Succesional.Hardwood.Forest | 0.375838 | -0.90235 |
| 100 | Xeric.Hammock | 0.670207 | -0.43115 |
| 100 | Improved | 0.258205 | 1.158133 |
| 100 | Abandoned.Field.Pasture | 0.190186 | 1.348185 |
| 100 | Scrubby.Flatwoods | 0.365419 | 0.922561 |
| 100 | Clastic.Upland.Lake | 0.049596 | 2.072635 |
| 200 | Basin.Marsh | 0.567786 | 0.573274 |
| 200 | Basin.Swamp | 0.00056 | -3.56841 |
| 200 | Baygall | 0.130521 | -1.52497 |
| 200 | Lake.Bottom | 0.624068 | 0.491663 |
| 200 | Mesic.Hammock | 0.000211 | -3.85085 |
| 200 | Pine.Plantation | 0.285508 | 1.073953 |
| 200 | Sandhill | 0.050516 | -1.98018 |
| 200 | Sandhill.Upland.Lake | 0.05351 | -1.95459 |
| 200 | Succesional.Hardwood.Forest | 0.448733 | -0.76061 |
| 200 | Xeric.Hammock | 0.009002 | -2.66565 |
| 200 | Improved | 0.611908 | 0.509001 |
| 200 | Abandoned.Field.Pasture | 0.307968 | 1.024883 |
| 200 | Scrubby.Flatwoods | 0.6579 | 0.444182 |
| 200 | Clastic.Upland.Lake | 2.54E-08 | 6.065135 |
| 300 | Basin.Marsh | 0.93536 | 0.081835 |
| 300 | Basin.Swamp | 0.243229 | -1.1921 |
| 300 | Baygall | 0.478368 | -0.71856 |
| 300 | Lake.Bottom | 0.968223 | -0.0402 |
| 300 | Mesic.Hammock | 0.650361 | -0.45818 |
| 300 | Pine.Plantation | 0.817114 | 0.233445 |
| 300 | Sandhill | 0.988689 | -0.0143 |
| 300 | Sandhill.Upland.Lake | 0.397784 | -0.85872 |
| 300 | Succesional.Hardwood.Forest | 0.903927 | -0.1218 |
| 300 | Xeric.Hammock | 0.593201 | -0.54039 |
| 300 | Improved | 0.829333 | -0.21758 |
| 300 | Abandoned.Field.Pasture | 0.628236 | -0.48959 |
| 300 | Scrubby.Flatwoods | 0.712858 | -0.37178 |
| 300 | Clastic.Upland.Lake | 0.231701 | 1.222523 |
| 400 | Basin.Marsh | 0.014284 | -2.4941 |
| 400 | Basin.Swamp | 7.07E-05 | -4.14911 |
| 400 | Baygall | 6.19E-05 | -4.18471 |
| 400 | Lake.Bottom | 0.035654 | -2.12995 |
| 400 | Mesic.Hammock | 3.00E-06 | -4.95365 |
| 400 | Pine.Plantation | 0.142192 | -1.47946 |
| 400 | Sandhill | 0.822113 | 0.225425 |
| 400 | Sandhill.Upland.Lake | 1.93E-05 | -4.49025 |
| 400 | Succesional.Hardwood.Forest | 0.00024 | -3.8114 |
| 400 | Xeric.Hammock | 1.23E-11 | -7.66778 |
| 400 | Improved | 0.843718 | 0.197658 |
| 400 | Abandoned.Field.Pasture | 0.058709 | -1.91245 |
| 400 | Scrubby.Flatwoods | 0.000104 | -4.04346 |
| 400 | Clastic.Upland.Lake | 1.01E-08 | 6.255775 |
| 500 | Basin.Marsh | 0.603593 | 0.522553 |
| 500 | Basin.Swamp | 0.778498 | -0.2828 |
| 500 | Baygall | 0.12752 | -1.5497 |
| 500 | Lake.Bottom | 0.115169 | -1.60329 |
| 500 | Mesic.Hammock | 0.28704 | -1.07612 |
| 500 | Pine.Plantation | 0.582037 | -0.55402 |
| 500 | Sandhill | 0.849463 | -0.19079 |
| 500 | Sandhill.Upland.Lake | 0.041824 | -2.08894 |
| 500 | Succesional.Hardwood.Forest | 0.969169 | -0.03884 |
| 500 | Xeric.Hammock | 0.469405 | -0.729 |
| 500 | Improved | 0.440729 | 0.777155 |
| 500 | Abandoned.Field.Pasture | 0.558875 | -0.58846 |
| 500 | Scrubby.Flatwoods | 0.583329 | -0.55212 |
| 500 | Clastic.Upland.Lake | 0.409856 | 0.831123 |

Table S17: Numbers and proportions of replicates showing significant phylogenetic patterns of NRI for reconstructed phylogenies of randomly sampled subsets for each community (Question 4). Results are separated into clustered and overdispersed patterns.

| Community | Subset | Number of replicates | Number Overdispersed | Proportion Overdispersed | Number Clustered | Proportion Clustered |
| --- | --- | --- | --- | --- | --- | --- |
| Basin.Marsh | 100 | 25 | 1 | 0.04 | 4 | 0.16 |
| Basin.Swamp | 100 | 25 | 5 | 0.2 | 3 | 0.12 |
| Baygall | 100 | 25 | 1 | 0.04 | 6 | 0.24 |
| Lake.Bottom | 100 | 25 | 4 | 0.16 | 0 | 0 |
| Mesic.Hammock | 100 | 25 | 2 | 0.08 | 5 | 0.2 |
| Pine.Plantation | 100 | 25 | 0 | 0 | 3 | 0.12 |
| Sandhill | 100 | 25 | 0 | 0 | 8 | 0.32 |
| Sandhill.Upland.Lake | 100 | 25 | 5 | 0.2 | 0 | 0 |
| Succesional.Hardwood.Forest | 100 | 25 | 1 | 0.04 | 0 | 0 |
| Xeric.Hammock | 100 | 25 | 0 | 0 | 6 | 0.24 |
| Improved | 100 | 25 | 0 | 0 | 1 | 0.04 |
| Abandoned.Field.Pasture | 100 | 25 | 0 | 0 | 6 | 0.24 |
| Scrubby.Flatwoods | 100 | 25 | 0 | 0 | 4 | 0.16 |
| Clastic.Upland.Lake | 100 | 25 | 0 | 0 | 3 | 0.12 |
| Basin.Marsh | 200 | 98 | 0 | 0 | 16 | 0.16 |
| Basin.Swamp | 200 | 98 | 23 | 0.23 | 6 | 0.06 |
| Baygall | 200 | 98 | 0 | 0 | 55 | 0.56 |
| Lake.Bottom | 200 | 98 | 6 | 0.06 | 1 | 0.01 |
| Mesic.Hammock | 200 | 98 | 4 | 0.04 | 11 | 0.11 |
| Pine.Plantation | 200 | 98 | 0 | 0 | 14 | 0.14 |
| Sandhill | 200 | 98 | 0 | 0 | 46 | 0.47 |
| Sandhill.Upland.Lake | 200 | 98 | 24 | 0.24 | 0 | 0 |
| Succesional.Hardwood.Forest | 200 | 98 | 6 | 0.06 | 2 | 0.02 |
| Xeric.Hammock | 200 | 98 | 0 | 0 | 48 | 0.49 |
| Improved | 200 | 98 | 0 | 0 | 5 | 0.05 |
| Abandoned.Field.Pasture | 200 | 98 | 0 | 0 | 6 | 0.06 |
| Scrubby.Flatwoods | 200 | 98 | 0 | 0 | 36 | 0.37 |
| Clastic.Upland.Lake | 200 | 98 | 0 | 0 | 27 | 0.28 |
| Basin.Marsh | 300 | 29 | 0 | 0 | 4 | 0.14 |
| Basin.Swamp | 300 | 29 | 13 | 0.45 | 1 | 0.03 |
| Baygall | 300 | 29 | 0 | 0 | 10 | 0.34 |
| Lake.Bottom | 300 | 29 | 0 | 0 | 0 | 0 |
| Mesic.Hammock | 300 | 29 | 0 | 0 | 0 | 0 |
| Pine.Plantation | 300 | 29 | 0 | 0 | 5 | 0.17 |
| Sandhill | 300 | 29 | 0 | 0 | 21 | 0.72 |
| Sandhill.Upland.Lake | 300 | 29 | 9 | 0.31 | 0 | 0 |
| Succesional.Hardwood.Forest | 300 | 29 | 2 | 0.07 | 0 | 0 |
| Xeric.Hammock | 300 | 29 | 0 | 0 | 15 | 0.52 |
| Improved | 300 | 29 | 0 | 0 | 2 | 0.07 |
| Abandoned.Field.Pasture | 300 | 29 | 0 | 0 | 8 | 0.28 |
| Scrubby.Flatwoods | 300 | 29 | 0 | 0 | 11 | 0.38 |
| Clastic.Upland.Lake | 300 | 29 | 0 | 0 | 12 | 0.41 |
| Basin.Marsh | 400 | 100 | 0 | 0 | 8 | 0.08 |
| Basin.Swamp | 400 | 100 | 65 | 0.65 | 0 | 0 |
| Baygall | 400 | 100 | 0 | 0 | 60 | 0.6 |
| Lake.Bottom | 400 | 100 | 1 | 0.01 | 0 | 0 |
| Mesic.Hammock | 400 | 100 | 7 | 0.07 | 1 | 0.01 |
| Pine.Plantation | 400 | 100 | 0 | 0 | 12 | 0.12 |
| Sandhill | 400 | 100 | 0 | 0 | 94 | 0.94 |
| Sandhill.Upland.Lake | 400 | 100 | 39 | 0.39 | 0 | 0 |
| Succesional.Hardwood.Forest | 400 | 100 | 4 | 0.04 | 0 | 0 |
| Xeric.Hammock | 400 | 100 | 0 | 0 | 42 | 0.42 |
| Improved | 400 | 100 | 0 | 0 | 6 | 0.06 |
| Abandoned.Field.Pasture | 400 | 100 | 0 | 0 | 16 | 0.16 |
| Scrubby.Flatwoods | 400 | 100 | 0 | 0 | 55 | 0.55 |
| Clastic.Upland.Lake | 400 | 100 | 0 | 0 | 69 | 0.69 |
| Basin.Marsh | 500 | 51 | 0 | 0 | 6 | 0.12 |
| Basin.Swamp | 500 | 51 | 31 | 0.61 | 0 | 0 |
| Baygall | 500 | 51 | 0 | 0 | 44 | 0.86 |
| Lake.Bottom | 500 | 51 | 0 | 0 | 0 | 0 |
| Mesic.Hammock | 500 | 51 | 0 | 0 | 0 | 0 |
| Pine.Plantation | 500 | 51 | 0 | 0 | 6 | 0.12 |
| Sandhill | 500 | 51 | 0 | 0 | 51 | 1 |
| Sandhill.Upland.Lake | 500 | 51 | 16 | 0.31 | 0 | 0 |
| Succesional.Hardwood.Forest | 500 | 51 | 0 | 0 | 0 | 0 |
| Xeric.Hammock | 500 | 51 | 0 | 0 | 42 | 0.82 |
| Improved | 500 | 51 | 0 | 0 | 0 | 0 |
| Abandoned.Field.Pasture | 500 | 51 | 0 | 0 | 3 | 0.06 |
| Scrubby.Flatwoods | 500 | 51 | 0 | 0 | 40 | 0.78 |
| Clastic.Upland.Lake | 500 | 51 | 0 | 0 | 46 | 0.9 |

Table S18: Numbers and proportions of replicates showing significant phylogenetic patterns of NTI for reconstructed phylogenies of randomly sampled subsets for each community (Question 4). Results are separated into clustered and overdispersed patterns.

| Community | Subset | Number of replicates | Number Overdispersed | Proportion Overdispersed | Number Clustered | Proportion Clustered |
| --- | --- | --- | --- | --- | --- | --- |
| Basin.Marsh | 100 | 25 | 1 | 0.04 | 0 | 0 |
| Basin.Swamp | 100 | 25 | 2 | 0.08 | 0 | 0 |
| Baygall | 100 | 25 | 0 | 0 | 5 | 0.2 |
| Lake.Bottom | 100 | 25 | 0 | 0 | 4 | 0.16 |
| Mesic.Hammock | 100 | 25 | 0 | 0 | 3 | 0.12 |
| Pine.Plantation | 100 | 25 | 0 | 0 | 5 | 0.2 |
| Sandhill | 100 | 25 | 0 | 0 | 14 | 0.56 |
| Sandhill.Upland.Lake | 100 | 25 | 2 | 0.08 | 0 | 0 |
| Succesional.Hardwood.Forest | 100 | 25 | 2 | 0.08 | 1 | 0.04 |
| Xeric.Hammock | 100 | 25 | 0 | 0 | 6 | 0.24 |
| Improved | 100 | 25 | 0 | 0 | 1 | 0.04 |
| Abandoned.Field.Pasture | 100 | 25 | 0 | 0 | 1 | 0.04 |
| Scrubby.Flatwoods | 100 | 25 | 0 | 0 | 4 | 0.16 |
| Clastic.Upland.Lake | 100 | 25 | 0 | 0 | 1 | 0.04 |
| Basin.Marsh | 200 | 98 | 4 | 0.04 | 2 | 0.02 |
| Basin.Swamp | 200 | 98 | 4 | 0.04 | 2 | 0.02 |
| Baygall | 200 | 98 | 0 | 0 | 19 | 0.19 |
| Lake.Bottom | 200 | 98 | 0 | 0 | 18 | 0.18 |
| Mesic.Hammock | 200 | 98 | 0 | 0 | 8 | 0.08 |
| Pine.Plantation | 200 | 98 | 0 | 0 | 25 | 0.26 |
| Sandhill | 200 | 98 | 0 | 0 | 78 | 0.8 |
| Sandhill.Upland.Lake | 200 | 98 | 8 | 0.08 | 5 | 0.05 |
| Succesional.Hardwood.Forest | 200 | 98 | 4 | 0.04 | 4 | 0.04 |
| Xeric.Hammock | 200 | 98 | 0 | 0 | 48 | 0.49 |
| Improved | 200 | 98 | 0 | 0 | 23 | 0.23 |
| Abandoned.Field.Pasture | 200 | 98 | 1 | 0.01 | 2 | 0.02 |
| Scrubby.Flatwoods | 200 | 98 | 0 | 0 | 50 | 0.51 |
| Clastic.Upland.Lake | 200 | 98 | 0 | 0 | 4 | 0.04 |
| Basin.Marsh | 300 | 29 | 3 | 0.1 | 1 | 0.03 |
| Basin.Swamp | 300 | 29 | 0 | 0 | 0 | 0 |
| Baygall | 300 | 29 | 0 | 0 | 7 | 0.24 |
| Lake.Bottom | 300 | 29 | 0 | 0 | 19 | 0.66 |
| Mesic.Hammock | 300 | 29 | 0 | 0 | 7 | 0.24 |
| Pine.Plantation | 300 | 29 | 0 | 0 | 16 | 0.55 |
| Sandhill | 300 | 29 | 0 | 0 | 28 | 0.97 |
| Sandhill.Upland.Lake | 300 | 29 | 0 | 0 | 6 | 0.21 |
| Succesional.Hardwood.Forest | 300 | 29 | 0 | 0 | 1 | 0.03 |
| Xeric.Hammock | 300 | 29 | 0 | 0 | 11 | 0.38 |
| Improved | 300 | 29 | 0 | 0 | 8 | 0.28 |
| Abandoned.Field.Pasture | 300 | 29 | 0 | 0 | 2 | 0.07 |
| Scrubby.Flatwoods | 300 | 29 | 0 | 0 | 24 | 0.83 |
| Clastic.Upland.Lake | 300 | 29 | 0 | 0 | 2 | 0.07 |
| Basin.Marsh | 400 | 100 | 4 | 0.04 | 0 | 0 |
| Basin.Swamp | 400 | 100 | 0 | 0 | 1 | 0.01 |
| Baygall | 400 | 100 | 0 | 0 | 32 | 0.32 |
| Lake.Bottom | 400 | 100 | 0 | 0 | 57 | 0.57 |
| Mesic.Hammock | 400 | 100 | 0 | 0 | 20 | 0.2 |
| Pine.Plantation | 400 | 100 | 0 | 0 | 64 | 0.64 |
| Sandhill | 400 | 100 | 0 | 0 | 99 | 0.99 |
| Sandhill.Upland.Lake | 400 | 100 | 0 | 0 | 62 | 0.62 |
| Succesional.Hardwood.Forest | 400 | 100 | 0 | 0 | 2 | 0.02 |
| Xeric.Hammock | 400 | 100 | 0 | 0 | 57 | 0.57 |
| Improved | 400 | 100 | 0 | 0 | 68 | 0.68 |
| Abandoned.Field.Pasture | 400 | 100 | 0 | 0 | 2 | 0.02 |
| Scrubby.Flatwoods | 400 | 100 | 0 | 0 | 97 | 0.97 |
| Clastic.Upland.Lake | 400 | 100 | 0 | 0 | 4 | 0.04 |
| Basin.Marsh | 500 | 51 | 0 | 0 | 0 | 0 |
| Basin.Swamp | 500 | 51 | 0 | 0 | 0 | 0 |
| Baygall | 500 | 51 | 0 | 0 | 14 | 0.27 |
| Lake.Bottom | 500 | 51 | 0 | 0 | 44 | 0.86 |
| Mesic.Hammock | 500 | 51 | 0 | 0 | 7 | 0.14 |
| Pine.Plantation | 500 | 51 | 0 | 0 | 46 | 0.9 |
| Sandhill | 500 | 51 | 0 | 0 | 51 | 1 |
| Sandhill.Upland.Lake | 500 | 51 | 0 | 0 | 48 | 0.94 |
| Succesional.Hardwood.Forest | 500 | 51 | 0 | 0 | 0 | 0 |
| Xeric.Hammock | 500 | 51 | 0 | 0 | 45 | 0.88 |
| Improved | 500 | 51 | 0 | 0 | 46 | 0.9 |
| Abandoned.Field.Pasture | 500 | 51 | 0 | 0 | 0 | 0 |
| Scrubby.Flatwoods | 500 | 51 | 0 | 0 | 51 | 1 |
| Clastic.Upland.Lake | 500 | 51 | 0 | 0 | 0 | 0 |

Table S19: Numbers and proportions of replicates showing significant phylogenetic patterns of PD_SES_ for reconstructed phylogenies of randomly sampled subsets for each community (Question 4). Results are separated into clustered and overdispersed patterns.

| Community | Subset | Number of replicates | Number Overdispersed | Proportion Overdispersed | Number Clustered | Proportion Clustered |
| --- | --- | --- | --- | --- | --- | --- |
| Basin.Marsh | 100 | 25 | 1 | 0.04 | 0 | 0 |
| Basin.Swamp | 100 | 25 | 6 | 0.24 | 0 | 0 |
| Baygall | 100 | 25 | 2 | 0.08 | 0 | 0 |
| Lake.Bottom | 100 | 25 | 3 | 0.12 | 0 | 0 |
| Mesic.Hammock | 100 | 25 | 0 | 0 | 0 | 0 |
| Pine.Plantation | 100 | 25 | 5 | 0.2 | 0 | 0 |
| Sandhill | 100 | 25 | 0 | 0 | 3 | 0.12 |
| Sandhill.Upland.Lake | 100 | 25 | 9 | 0.36 | 0 | 0 |
| Succesional.Hardwood.Forest | 100 | 25 | 2 | 0.08 | 0 | 0 |
| Xeric.Hammock | 100 | 25 | 1 | 0.04 | 1 | 0.04 |
| Improved | 100 | 25 | 0 | 0 | 0 | 0 |
| Abandoned.Field.Pasture | 100 | 25 | 0 | 0 | 0 | 0 |
| Scrubby.Flatwoods | 100 | 25 | 4 | 0.16 | 0 | 0 |
| Clastic.Upland.Lake | 100 | 25 | 4 | 0.16 | 0 | 0 |
| Basin.Marsh | 200 | 98 | 3 | 0.03 | 0 | 0 |
| Basin.Swamp | 200 | 98 | 7 | 0.07 | 0 | 0 |
| Baygall | 200 | 98 | 0 | 0 | 6 | 0.06 |
| Lake.Bottom | 200 | 98 | 2 | 0.02 | 0 | 0 |
| Mesic.Hammock | 200 | 98 | 3 | 0.03 | 1 | 0.01 |
| Pine.Plantation | 200 | 98 | 1 | 0.01 | 0 | 0 |
| Sandhill | 200 | 98 | 0 | 0 | 63 | 0.64 |
| Sandhill.Upland.Lake | 200 | 98 | 22 | 0.22 | 0 | 0 |
| Succesional.Hardwood.Forest | 200 | 98 | 14 | 0.14 | 0 | 0 |
| Xeric.Hammock | 200 | 98 | 0 | 0 | 24 | 0.24 |
| Improved | 200 | 98 | 0 | 0 | 5 | 0.05 |
| Abandoned.Field.Pasture | 200 | 98 | 1 | 0.01 | 0 | 0 |
| Scrubby.Flatwoods | 200 | 98 | 0 | 0 | 4 | 0.04 |
| Clastic.Upland.Lake | 200 | 98 | 0 | 0 | 0 | 0 |
| Basin.Marsh | 300 | 29 | 2 | 0.07 | 0 | 0 |
| Basin.Swamp | 300 | 29 | 0 | 0 | 0 | 0 |
| Baygall | 300 | 29 | 0 | 0 | 4 | 0.14 |
| Lake.Bottom | 300 | 29 | 0 | 0 | 3 | 0.1 |
| Mesic.Hammock | 300 | 29 | 0 | 0 | 0 | 0 |
| Pine.Plantation | 300 | 29 | 0 | 0 | 0 | 0 |
| Sandhill | 300 | 29 | 0 | 0 | 28 | 0.97 |
| Sandhill.Upland.Lake | 300 | 29 | 3 | 0.1 | 0 | 0 |
| Succesional.Hardwood.Forest | 300 | 29 | 4 | 0.14 | 0 | 0 |
| Xeric.Hammock | 300 | 29 | 0 | 0 | 7 | 0.24 |
| Improved | 300 | 29 | 0 | 0 | 4 | 0.14 |
| Abandoned.Field.Pasture | 300 | 29 | 0 | 0 | 0 | 0 |
| Scrubby.Flatwoods | 300 | 29 | 0 | 0 | 6 | 0.21 |
| Clastic.Upland.Lake | 300 | 29 | 0 | 0 | 0 | 0 |
| Basin.Marsh | 400 | 100 | 8 | 0.08 | 0 | 0 |
| Basin.Swamp | 400 | 100 | 0 | 0 | 0 | 0 |
| Baygall | 400 | 100 | 0 | 0 | 14 | 0.14 |
| Lake.Bottom | 400 | 100 | 0 | 0 | 3 | 0.03 |
| Mesic.Hammock | 400 | 100 | 0 | 0 | 0 | 0 |
| Pine.Plantation | 400 | 100 | 0 | 0 | 1 | 0.01 |
| Sandhill | 400 | 100 | 0 | 0 | 100 | 1 |
| Sandhill.Upland.Lake | 400 | 100 | 1 | 0.01 | 0 | 0 |
| Succesional.Hardwood.Forest | 400 | 100 | 4 | 0.04 | 0 | 0 |
| Xeric.Hammock | 400 | 100 | 0 | 0 | 17 | 0.17 |
| Improved | 400 | 100 | 0 | 0 | 53 | 0.53 |
| Abandoned.Field.Pasture | 400 | 100 | 0 | 0 | 0 | 0 |
| Scrubby.Flatwoods | 400 | 100 | 0 | 0 | 55 | 0.55 |
| Clastic.Upland.Lake | 400 | 100 | 0 | 0 | 0 | 0 |
| Basin.Marsh | 500 | 51 | 1 | 0.02 | 0 | 0 |
| Basin.Swamp | 500 | 51 | 0 | 0 | 0 | 0 |
| Baygall | 500 | 51 | 0 | 0 | 5 | 0.1 |
| Lake.Bottom | 500 | 51 | 0 | 0 | 4 | 0.08 |
| Mesic.Hammock | 500 | 51 | 0 | 0 | 0 | 0 |
| Pine.Plantation | 500 | 51 | 0 | 0 | 0 | 0 |
| Sandhill | 500 | 51 | 0 | 0 | 51 | 1 |
| Sandhill.Upland.Lake | 500 | 51 | 0 | 0 | 0 | 0 |
| Succesional.Hardwood.Forest | 500 | 51 | 1 | 0.02 | 0 | 0 |
| Xeric.Hammock | 500 | 51 | 0 | 0 | 16 | 0.31 |
| Improved | 500 | 51 | 0 | 0 | 49 | 0.96 |
| Abandoned.Field.Pasture | 500 | 51 | 0 | 0 | 0 | 0 |
| Scrubby.Flatwoods | 500 | 51 | 0 | 0 | 48 | 0.94 |
| Clastic.Upland.Lake | 500 | 51 | 0 | 0 | 0 | 0 |

Table S20: Results of Student’s t-test comparing the values of NRI for chronograms and phylograms for randomly sampled subsets for 14 communities (Question 5).

| Subset | Community | P-value | Statistic |
| --- | --- | --- | --- |
| 100 | Basin.Marsh | 1.29E-14 | 9.049261 |
| 100 | Basin.Swamp | 2.29E-22 | 12.67187 |
| 100 | Baygall | 2.19E-35 | 19.33366 |
| 100 | Lake.Bottom | 0.000153 | 3.938818 |
| 100 | Mesic.Hammock | 1.11E-22 | 12.78356 |
| 100 | Pine.Plantation | 2.25E-20 | 12.1169 |
| 100 | Sandhill | 0.006233 | -2.79504 |
| 100 | Sandhill.Upland.Lake | 0.771092 | 0.291745 |
| 100 | Succesional.Hardwood.Forest | 8.75E-14 | 8.667106 |
| 100 | Xeric.Hammock | 1.03E-26 | 14.73954 |
| 100 | Improved | 0.79966 | 0.25447 |
| 100 | Abandoned.Field.Pasture | 2.27E-21 | 12.16627 |
| 100 | Scrubby.Flatwoods | 3.07E-18 | 10.76154 |
| 100 | Clastic.Upland.Lake | 1.58E-15 | 9.522198 |
| 200 | Basin.Marsh | 1.27E-30 | 16.7362 |
| 200 | Basin.Swamp | 1.25E-38 | 21.22821 |
| 200 | Baygall | 9.95E-56 | 33.424 |
| 200 | Lake.Bottom | 5.49E-08 | 5.883178 |
| 200 | Mesic.Hammock | 7.92E-43 | 23.85113 |
| 200 | Pine.Plantation | 9.23E-35 | 18.98143 |
| 200 | Sandhill | 1.73E-05 | -4.51772 |
| 200 | Sandhill.Upland.Lake | 0.813873 | 0.236061 |
| 200 | Succesional.Hardwood.Forest | 4.80E-22 | 12.48358 |
| 200 | Xeric.Hammock | 2.35E-49 | 28.38004 |
| 200 | Improved | 0.542899 | 0.610546 |
| 200 | Abandoned.Field.Pasture | 9.71E-30 | 16.27487 |
| 200 | Scrubby.Flatwoods | 1.47E-33 | 18.3131 |
| 200 | Clastic.Upland.Lake | 2.23E-29 | 16.08874 |
| 300 | Basin.Marsh | 3.34E-36 | 19.80045 |
| 300 | Basin.Swamp | 1.92E-48 | 27.71104 |
| 300 | Baygall | 3.04E-67 | 44.47629 |
| 300 | Lake.Bottom | 1.84E-16 | 9.896913 |
| 300 | Mesic.Hammock | 1.95E-50 | 29.18892 |
| 300 | Pine.Plantation | 3.12E-60 | 37.42166 |
| 300 | Sandhill | 2.96E-08 | -6.02075 |
| 300 | Sandhill.Upland.Lake | 0.568432 | -0.57228 |
| 300 | Succesional.Hardwood.Forest | 1.01E-25 | 14.25049 |
| 300 | Xeric.Hammock | 2.64E-66 | 43.46815 |
| 300 | Improved | 0.364898 | 0.910256 |
| 300 | Abandoned.Field.Pasture | 9.10E-44 | 24.46756 |
| 300 | Scrubby.Flatwoods | 3.25E-50 | 29.02138 |
| 300 | Clastic.Upland.Lake | 3.37E-47 | 26.81772 |
| 400 | Basin.Marsh | 5.55E-57 | 34.49847 |
| 400 | Basin.Swamp | 9.73E-56 | 33.43238 |
| 400 | Baygall | 7.26E-88 | 72.94029 |
| 400 | Lake.Bottom | 8.11E-28 | 15.29302 |
| 400 | Mesic.Hammock | 3.04E-65 | 42.35167 |
| 400 | Pine.Plantation | 4.59E-65 | 42.16686 |
| 400 | Sandhill | 4.01E-06 | -4.88359 |
| 400 | Sandhill.Upland.Lake | 0.006468 | -2.78212 |
| 400 | Succesional.Hardwood.Forest | 8.71E-31 | 16.82175 |
| 400 | Xeric.Hammock | 1.92E-74 | 52.9521 |
| 400 | Improved | 0.156779 | 1.426816 |
| 400 | Abandoned.Field.Pasture | 8.56E-63 | 39.87469 |
| 400 | Scrubby.Flatwoods | 2.64E-56 | 33.91459 |
| 400 | Clastic.Upland.Lake | 9.87E-55 | 32.59029 |
| 500 | Basin.Marsh | 6.38E-66 | 43.06143 |
| 500 | Basin.Swamp | 3.98E-78 | 57.84845 |
| 500 | Baygall | 8.18E-109 | 119.4093 |
| 500 | Lake.Bottom | 1.23E-40 | 22.45521 |
| 500 | Mesic.Hammock | 1.22E-79 | 59.98051 |
| 500 | Pine.Plantation | 9.16E-77 | 55.99023 |
| 500 | Sandhill | 1.54E-12 | -8.08996 |
| 500 | Sandhill.Upland.Lake | 0.077287 | -1.78522 |
| 500 | Succesional.Hardwood.Forest | 4.05E-50 | 28.94968 |
| 500 | Xeric.Hammock | 1.12E-86 | 70.91641 |
| 500 | Improved | 0.460837 | 0.740355 |
| 500 | Abandoned.Field.Pasture | 2.19E-78 | 58.20946 |
| 500 | Scrubby.Flatwoods | 5.59E-72 | 49.89727 |
| 500 | Clastic.Upland.Lake | 6.39E-74 | 52.2912 |

Table S21: Results of Student’s t-test comparing the values of NTI for chronograms and phylograms for randomly sampled subsets for 14 communities (Question 5).

| Subset | Community | P-value | Statistic |
| --- | --- | --- | --- |
| 100 | Basin.Marsh | 3.23E-10 | 6.990919 |
| 100 | Basin.Swamp | 4.85E-32 | 17.56634 |
| 100 | Baygall | 1.23E-39 | 21.83883 |
| 100 | Lake.Bottom | 0.033022 | 2.162069 |
| 100 | Mesic.Hammock | 2.01E-20 | 11.72299 |
| 100 | Pine.Plantation | 3.46E-06 | 4.95991 |
| 100 | Sandhill | 4.24E-05 | -4.28522 |
| 100 | Sandhill.Upland.Lake | 0.000799 | -3.45974 |
| 100 | Succesional.Hardwood.Forest | 0.198632 | -1.29413 |
| 100 | Xeric.Hammock | 5.17E-11 | 7.372246 |
| 100 | Improved | 0.274893 | 1.097943 |
| 100 | Abandoned.Field.Pasture | 1.40E-15 | 9.49215 |
| 100 | Scrubby.Flatwoods | 4.85E-10 | 6.920938 |
| 100 | Clastic.Upland.Lake | 4.04E-16 | 9.798143 |
| 200 | Basin.Marsh | 1.47E-16 | 9.94105 |
| 200 | Basin.Swamp | 1.51E-45 | 25.66645 |
| 200 | Baygall | 1.51E-44 | 24.98866 |
| 200 | Lake.Bottom | 0.034194 | -2.14751 |
| 200 | Mesic.Hammock | 8.08E-28 | 15.29374 |
| 200 | Pine.Plantation | 4.72E-06 | 4.843101 |
| 200 | Sandhill | 4.28E-07 | -5.41565 |
| 200 | Sandhill.Upland.Lake | 4.94E-12 | -7.85334 |
| 200 | Succesional.Hardwood.Forest | 0.002664 | -3.08194 |
| 200 | Xeric.Hammock | 6.91E-06 | 4.749028 |
| 200 | Improved | 0.008883 | 2.669346 |
| 200 | Abandoned.Field.Pasture | 2.78E-23 | 13.0708 |
| 200 | Scrubby.Flatwoods | 0.002968 | 3.046485 |
| 200 | Clastic.Upland.Lake | 4.70E-29 | 15.92184 |
| 300 | Basin.Marsh | 4.26E-07 | 5.416901 |
| 300 | Basin.Swamp | 3.53E-51 | 29.75584 |
| 300 | Baygall | 1.12E-40 | 22.48074 |
| 300 | Lake.Bottom | 0.81823 | -0.23043 |
| 300 | Mesic.Hammock | 1.34E-36 | 20.02883 |
| 300 | Pine.Plantation | 0.395095 | 0.854131 |
| 300 | Sandhill | 0.007457 | -2.73192 |
| 300 | Sandhill.Upland.Lake | 3.30E-13 | -8.40092 |
| 300 | Succesional.Hardwood.Forest | 2.56E-08 | -6.05221 |
| 300 | Xeric.Hammock | 0.014769 | 2.481453 |
| 300 | Improved | 7.76E-05 | 4.1237 |
| 300 | Abandoned.Field.Pasture | 2.20E-33 | 18.21732 |
| 300 | Scrubby.Flatwoods | 0.164266 | 1.401253 |
| 300 | Clastic.Upland.Lake | 1.36E-45 | 25.69919 |
| 400 | Basin.Marsh | 0.235592 | 1.19333 |
| 400 | Basin.Swamp | 1.80E-62 | 39.55893 |
| 400 | Baygall | 6.39E-45 | 25.24022 |
| 400 | Lake.Bottom | 0.627283 | -0.48707 |
| 400 | Mesic.Hammock | 1.20E-39 | 21.84458 |
| 400 | Pine.Plantation | 0.309612 | -1.02128 |
| 400 | Sandhill | 8.45E-05 | -4.10067 |
| 400 | Sandhill.Upland.Lake | 4.37E-15 | -9.26579 |
| 400 | Succesional.Hardwood.Forest | 1.56E-11 | -7.61914 |
| 400 | Xeric.Hammock | 0.000308 | -3.74086 |
| 400 | Improved | 9.32E-12 | 7.723923 |
| 400 | Abandoned.Field.Pasture | 4.11E-48 | 27.47172 |
| 400 | Scrubby.Flatwoods | 0.012555 | -2.54253 |
| 400 | Clastic.Upland.Lake | 1.06E-52 | 30.94396 |
| 500 | Basin.Marsh | 0.133668 | -1.5122 |
| 500 | Basin.Swamp | 7.97E-80 | 60.24575 |
| 500 | Baygall | 7.96E-57 | 34.36266 |
| 500 | Lake.Bottom | 0.657093 | -0.44527 |
| 500 | Mesic.Hammock | 1.02E-49 | 28.64824 |
| 500 | Pine.Plantation | 0.014093 | -2.49918 |
| 500 | Sandhill | 0.000354 | -3.7006 |
| 500 | Sandhill.Upland.Lake | 3.66E-13 | -8.37994 |
| 500 | Succesional.Hardwood.Forest | 1.00E-22 | -12.8055 |
| 500 | Xeric.Hammock | 1.62E-12 | -8.07966 |
| 500 | Improved | 2.92E-21 | 12.11462 |
| 500 | Abandoned.Field.Pasture | 1.08E-68 | 46.07338 |
| 500 | Scrubby.Flatwoods | 0.00018 | -3.89293 |
| 500 | Clastic.Upland.Lake | 1.02E-69 | 47.23765 |

Table S22: Results of Student’s t-test comparing the values of PD_SES_ for chronograms and phylograms for randomly sampled subsets for 14 communities (Question 5).

| Subset | Community | P-value | Statistic |
| --- | --- | --- | --- |
| 100 | Basin.Marsh | 1.12E-13 | 8.617075 |
| 100 | Basin.Swamp | 1.17E-20 | 11.86337 |
| 100 | Baygall | 1.05E-34 | 18.95037 |
| 100 | Lake.Bottom | 0.000119 | 4.007342 |
| 100 | Mesic.Hammock | 2.15E-15 | 9.40712 |
| 100 | Pine.Plantation | 2.45E-21 | 12.60837 |
| 100 | Sandhill | 1.88E-05 | -4.49716 |
| 100 | Sandhill.Upland.Lake | 0.000181 | -3.89097 |
| 100 | Succesional.Hardwood.Forest | 0.062491 | 1.884062 |
| 100 | Xeric.Hammock | 8.26E-23 | 12.84516 |
| 100 | Improved | 3.47E-05 | 4.337446 |
| 100 | Abandoned.Field.Pasture | 5.45E-23 | 12.9312 |
| 100 | Scrubby.Flatwoods | 9.36E-22 | 12.41633 |
| 100 | Clastic.Upland.Lake | 2.04E-22 | 12.77147 |
| 200 | Basin.Marsh | 8.66E-23 | 12.83544 |
| 200 | Basin.Swamp | 3.65E-35 | 19.20834 |
| 200 | Baygall | 4.86E-50 | 28.89031 |
| 200 | Lake.Bottom | 0.413645 | 0.820953 |
| 200 | Mesic.Hammock | 7.84E-30 | 16.32321 |
| 200 | Pine.Plantation | 2.42E-30 | 16.58894 |
| 200 | Sandhill | 1.79E-15 | -9.44364 |
| 200 | Sandhill.Upland.Lake | 1.70E-16 | -9.91201 |
| 200 | Succesional.Hardwood.Forest | 0.527772 | -0.63365 |
| 200 | Xeric.Hammock | 3.56E-24 | 13.49847 |
| 200 | Improved | 1.57E-07 | 5.646868 |
| 200 | Abandoned.Field.Pasture | 4.05E-31 | 16.9975 |
| 200 | Scrubby.Flatwoods | 2.33E-28 | 15.56706 |
| 200 | Clastic.Upland.Lake | 8.62E-35 | 18.998 |
| 300 | Basin.Marsh | 4.90E-11 | 7.383332 |
| 300 | Basin.Swamp | 1.49E-40 | 22.40426 |
| 300 | Baygall | 5.98E-56 | 33.61131 |
| 300 | Lake.Bottom | 0.021064 | 2.344223 |
| 300 | Mesic.Hammock | 1.81E-30 | 16.65498 |
| 300 | Pine.Plantation | 2.64E-32 | 17.63072 |
| 300 | Sandhill | 6.52E-12 | -7.79687 |
| 300 | Sandhill.Upland.Lake | 1.54E-29 | -16.171 |
| 300 | Succesional.Hardwood.Forest | 3.49E-07 | -5.46328 |
| 300 | Xeric.Hammock | 1.91E-16 | 9.888925 |
| 300 | Improved | 7.82E-11 | 7.286601 |
| 300 | Abandoned.Field.Pasture | 2.95E-41 | 22.84517 |
| 300 | Scrubby.Flatwoods | 2.82E-31 | 17.08072 |
| 300 | Clastic.Upland.Lake | 2.89E-44 | 24.79939 |
| 400 | Basin.Marsh | 7.01E-12 | 7.782196 |
| 400 | Basin.Swamp | 1.70E-58 | 35.83473 |
| 400 | Baygall | 6.77E-66 | 43.03451 |
| 400 | Lake.Bottom | 0.540584 | 0.614061 |
| 400 | Mesic.Hammock | 7.18E-41 | 22.60213 |
| 400 | Pine.Plantation | 8.92E-30 | 16.29404 |
| 400 | Sandhill | 3.61E-11 | -7.44614 |
| 400 | Sandhill.Upland.Lake | 6.62E-50 | -28.7897 |
| 400 | Succesional.Hardwood.Forest | 1.44E-10 | -7.16051 |
| 400 | Xeric.Hammock | 4.26E-10 | 6.932931 |
| 400 | Improved | 3.11E-16 | 9.792233 |
| 400 | Abandoned.Field.Pasture | 1.69E-59 | 36.74384 |
| 400 | Scrubby.Flatwoods | 1.86E-27 | 15.11114 |
| 400 | Clastic.Upland.Lake | 1.28E-56 | 34.18467 |
| 500 | Basin.Marsh | 1.82E-11 | 7.587377 |
| 500 | Basin.Swamp | 2.79E-83 | 65.41914 |
| 500 | Baygall | 4.58E-77 | 56.39669 |
| 500 | Lake.Bottom | 0.021487 | -2.33637 |
| 500 | Mesic.Hammock | 1.31E-58 | 35.93608 |
| 500 | Pine.Plantation | 8.25E-25 | 13.80581 |
| 500 | Sandhill | 1.05E-23 | -13.2731 |
| 500 | Sandhill.Upland.Lake | 2.67E-71 | -49.0837 |
| 500 | Succesional.Hardwood.Forest | 4.00E-22 | -12.5205 |
| 500 | Xeric.Hammock | 1.42E-07 | 5.668933 |
| 500 | Improved | 1.37E-21 | 12.26872 |
| 500 | Abandoned.Field.Pasture | 4.70E-73 | 51.21035 |
| 500 | Scrubby.Flatwoods | 1.25E-25 | 14.20599 |
| 500 | Clastic.Upland.Lake | 3.32E-71 | 48.97257 |

Table S23: Numbers and proportions of replicates showing significant phylogenetic patterns of NRI for chronograms of randomly sampled subsets for each community (Question 5). Results are separated into clustered and overdispersed patterns.

| Community | Subset | Number of replicates | Number Overdispersed | Proportion Overdispersed | Number Clustered | Proportion Clustered |
| --- | --- | --- | --- | --- | --- | --- |
| Basin.Marsh | 100 | 100 | 10 | 0.1 | 1 | 0.01 |
| Basin.Swamp | 100 | 100 | 49 | 0.49 | 0 | 0 |
| Baygall | 100 | 100 | 11 | 0.11 | 1 | 0.01 |
| Lake.Bottom | 100 | 100 | 10 | 0.1 | 1 | 0.01 |
| Mesic.Hammock | 100 | 100 | 28 | 0.28 | 0 | 0 |
| Pine.Plantation | 100 | 100 | 4 | 0.04 | 9 | 0.09 |
| Sandhill | 100 | 100 | 0 | 0 | 39 | 0.39 |
| Sandhill.Upland.Lake | 100 | 100 | 14 | 0.14 | 0 | 0 |
| Succesional.Hardwood.Forest | 100 | 100 | 15 | 0.15 | 4 | 0.04 |
| Xeric.Hammock | 100 | 100 | 4 | 0.04 | 17 | 0.17 |
| Improved | 100 | 100 | 0 | 0 | 11 | 0.11 |
| Abandoned.Field.Pasture | 100 | 100 | 2 | 0.02 | 7 | 0.07 |
| Scrubby.Flatwoods | 100 | 100 | 1 | 0.01 | 24 | 0.24 |
| Clastic.Upland.Lake | 100 | 100 | 0 | 0 | 0 | 0 |
| Basin.Marsh | 200 | 100 | 5 | 0.05 | 0 | 0 |
| Basin.Swamp | 200 | 100 | 70 | 0.7 | 0 | 0 |
| Baygall | 200 | 100 | 10 | 0.1 | 1 | 0.01 |
| Lake.Bottom | 200 | 100 | 15 | 0.15 | 2 | 0.02 |
| Mesic.Hammock | 200 | 100 | 43 | 0.43 | 0 | 0 |
| Pine.Plantation | 200 | 100 | 1 | 0.01 | 11 | 0.11 |
| Sandhill | 200 | 100 | 0 | 0 | 51 | 0.51 |
| Sandhill.Upland.Lake | 200 | 100 | 25 | 0.25 | 0 | 0 |
| Succesional.Hardwood.Forest | 200 | 100 | 21 | 0.21 | 1 | 0.01 |
| Xeric.Hammock | 200 | 100 | 4 | 0.04 | 12 | 0.12 |
| Improved | 200 | 100 | 0 | 0 | 16 | 0.16 |
| Abandoned.Field.Pasture | 200 | 100 | 4 | 0.04 | 4 | 0.04 |
| Scrubby.Flatwoods | 200 | 100 | 0 | 0 | 29 | 0.29 |
| Clastic.Upland.Lake | 200 | 100 | 0 | 0 | 0 | 0 |
| Basin.Marsh | 300 | 100 | 3 | 0.03 | 0 | 0 |
| Basin.Swamp | 300 | 100 | 93 | 0.93 | 0 | 0 |
| Baygall | 300 | 100 | 16 | 0.16 | 0 | 0 |
| Lake.Bottom | 300 | 100 | 9 | 0.09 | 0 | 0 |
| Mesic.Hammock | 300 | 100 | 64 | 0.64 | 0 | 0 |
| Pine.Plantation | 300 | 100 | 0 | 0 | 5 | 0.05 |
| Sandhill | 300 | 100 | 0 | 0 | 82 | 0.82 |
| Sandhill.Upland.Lake | 300 | 100 | 29 | 0.29 | 0 | 0 |
| Succesional.Hardwood.Forest | 300 | 100 | 16 | 0.16 | 0 | 0 |
| Xeric.Hammock | 300 | 100 | 1 | 0.01 | 3 | 0.03 |
| Improved | 300 | 100 | 0 | 0 | 9 | 0.09 |
| Abandoned.Field.Pasture | 300 | 100 | 0 | 0 | 4 | 0.04 |
| Scrubby.Flatwoods | 300 | 100 | 0 | 0 | 15 | 0.15 |
| Clastic.Upland.Lake | 300 | 100 | 0 | 0 | 0 | 0 |
| Basin.Marsh | 400 | 100 | 0 | 0 | 0 | 0 |
| Basin.Swamp | 400 | 100 | 98 | 0.98 | 0 | 0 |
| Baygall | 400 | 100 | 3 | 0.03 | 0 | 0 |
| Lake.Bottom | 400 | 100 | 5 | 0.05 | 0 | 0 |
| Mesic.Hammock | 400 | 100 | 81 | 0.81 | 0 | 0 |
| Pine.Plantation | 400 | 100 | 0 | 0 | 2 | 0.02 |
| Sandhill | 400 | 100 | 0 | 0 | 100 | 1 |
| Sandhill.Upland.Lake | 400 | 100 | 35 | 0.35 | 0 | 0 |
| Succesional.Hardwood.Forest | 400 | 100 | 25 | 0.25 | 0 | 0 |
| Xeric.Hammock | 400 | 100 | 1 | 0.01 | 2 | 0.02 |
| Improved | 400 | 100 | 0 | 0 | 4 | 0.04 |
| Abandoned.Field.Pasture | 400 | 100 | 0 | 0 | 0 | 0 |
| Scrubby.Flatwoods | 400 | 100 | 0 | 0 | 11 | 0.11 |
| Clastic.Upland.Lake | 400 | 100 | 0 | 0 | 0 | 0 |
| Basin.Marsh | 500 | 100 | 0 | 0 | 0 | 0 |
| Basin.Swamp | 500 | 100 | 100 | 1 | 0 | 0 |
| Baygall | 500 | 100 | 0 | 0 | 0 | 0 |
| Lake.Bottom | 500 | 100 | 0 | 0 | 0 | 0 |
| Mesic.Hammock | 500 | 100 | 97 | 0.97 | 0 | 0 |
| Pine.Plantation | 500 | 100 | 0 | 0 | 2 | 0.02 |
| Sandhill | 500 | 100 | 0 | 0 | 100 | 1 |
| Sandhill.Upland.Lake | 500 | 100 | 27 | 0.27 | 0 | 0 |
| Succesional.Hardwood.Forest | 500 | 100 | 17 | 0.17 | 0 | 0 |
| Xeric.Hammock | 500 | 100 | 0 | 0 | 0 | 0 |
| Improved | 500 | 100 | 0 | 0 | 2 | 0.02 |
| Abandoned.Field.Pasture | 500 | 100 | 0 | 0 | 0 | 0 |
| Scrubby.Flatwoods | 500 | 100 | 0 | 0 | 3 | 0.03 |
| Clastic.Upland.Lake | 500 | 100 | 0 | 0 | 0 | 0 |

Table S24: Numbers and proportions of replicates showing significant phylogenetic patterns of NTI for chronograms of randomly sampled subsets for each community (Question 5). Results are separated into clustered and overdispersed patterns.

| Community | Subset | Number of replicates | Number Overdispersed | Proportion Overdispersed | Number Clustered | Proportion Clustered |
| --- | --- | --- | --- | --- | --- | --- |
| Basin.Marsh | 100 | 100 | 9 | 0.09 | 1 | 0.01 |
| Basin.Swamp | 100 | 100 | 38 | 0.38 | 0 | 0 |
| Baygall | 100 | 100 | 16 | 0.16 | 4 | 0.04 |
| Lake.Bottom | 100 | 100 | 4 | 0.04 | 10 | 0.1 |
| Mesic.Hammock | 100 | 100 | 16 | 0.16 | 0 | 0 |
| Pine.Plantation | 100 | 100 | 4 | 0.04 | 18 | 0.18 |
| Sandhill | 100 | 100 | 0 | 0 | 48 | 0.48 |
| Sandhill.Upland.Lake | 100 | 100 | 8 | 0.08 | 3 | 0.03 |
| Succesional.Hardwood.Forest | 100 | 100 | 7 | 0.07 | 7 | 0.07 |
| Xeric.Hammock | 100 | 100 | 1 | 0.01 | 22 | 0.22 |
| Improved | 100 | 100 | 0 | 0 | 8 | 0.08 |
| Abandoned.Field.Pasture | 100 | 100 | 7 | 0.07 | 3 | 0.03 |
| Scrubby.Flatwoods | 100 | 100 | 2 | 0.02 | 21 | 0.21 |
| Clastic.Upland.Lake | 100 | 100 | 0 | 0 | 5 | 0.05 |
| Basin.Marsh | 200 | 100 | 14 | 0.14 | 1 | 0.01 |
| Basin.Swamp | 200 | 100 | 53 | 0.53 | 0 | 0 |
| Baygall | 200 | 100 | 16 | 0.16 | 2 | 0.02 |
| Lake.Bottom | 200 | 100 | 0 | 0 | 21 | 0.21 |
| Mesic.Hammock | 200 | 100 | 12 | 0.12 | 0 | 0 |
| Pine.Plantation | 200 | 100 | 1 | 0.01 | 23 | 0.23 |
| Sandhill | 200 | 100 | 0 | 0 | 89 | 0.89 |
| Sandhill.Upland.Lake | 200 | 100 | 5 | 0.05 | 10 | 0.1 |
| Succesional.Hardwood.Forest | 200 | 100 | 5 | 0.05 | 5 | 0.05 |
| Xeric.Hammock | 200 | 100 | 1 | 0.01 | 37 | 0.37 |
| Improved | 200 | 100 | 0 | 0 | 25 | 0.25 |
| Abandoned.Field.Pasture | 200 | 100 | 12 | 0.12 | 2 | 0.02 |
| Scrubby.Flatwoods | 200 | 100 | 0 | 0 | 45 | 0.45 |
| Clastic.Upland.Lake | 200 | 100 | 0 | 0 | 3 | 0.03 |
| Basin.Marsh | 300 | 100 | 18 | 0.18 | 1 | 0.01 |
| Basin.Swamp | 300 | 100 | 32 | 0.32 | 0 | 0 |
| Baygall | 300 | 100 | 14 | 0.14 | 4 | 0.04 |
| Lake.Bottom | 300 | 100 | 1 | 0.01 | 31 | 0.31 |
| Mesic.Hammock | 300 | 100 | 4 | 0.04 | 4 | 0.04 |
| Pine.Plantation | 300 | 100 | 0 | 0 | 38 | 0.38 |
| Sandhill | 300 | 100 | 0 | 0 | 95 | 0.95 |
| Sandhill.Upland.Lake | 300 | 100 | 0 | 0 | 33 | 0.33 |
| Succesional.Hardwood.Forest | 300 | 100 | 1 | 0.01 | 3 | 0.03 |
| Xeric.Hammock | 300 | 100 | 0 | 0 | 35 | 0.35 |
| Improved | 300 | 100 | 0 | 0 | 37 | 0.37 |
| Abandoned.Field.Pasture | 300 | 100 | 9 | 0.09 | 1 | 0.01 |
| Scrubby.Flatwoods | 300 | 100 | 0 | 0 | 60 | 0.6 |
| Clastic.Upland.Lake | 300 | 100 | 1 | 0.01 | 0 | 0 |
| Basin.Marsh | 400 | 100 | 10 | 0.1 | 0 | 0 |
| Basin.Swamp | 400 | 100 | 32 | 0.32 | 0 | 0 |
| Baygall | 400 | 100 | 11 | 0.11 | 2 | 0.02 |
| Lake.Bottom | 400 | 100 | 0 | 0 | 50 | 0.5 |
| Mesic.Hammock | 400 | 100 | 0 | 0 | 3 | 0.03 |
| Pine.Plantation | 400 | 100 | 0 | 0 | 65 | 0.65 |
| Sandhill | 400 | 100 | 0 | 0 | 100 | 1 |
| Sandhill.Upland.Lake | 400 | 100 | 0 | 0 | 75 | 0.75 |
| Succesional.Hardwood.Forest | 400 | 100 | 0 | 0 | 5 | 0.05 |
| Xeric.Hammock | 400 | 100 | 0 | 0 | 60 | 0.6 |
| Improved | 400 | 100 | 0 | 0 | 48 | 0.48 |
| Abandoned.Field.Pasture | 400 | 100 | 6 | 0.06 | 0 | 0 |
| Scrubby.Flatwoods | 400 | 100 | 0 | 0 | 92 | 0.92 |
| Clastic.Upland.Lake | 400 | 100 | 2 | 0.02 | 0 | 0 |
| Basin.Marsh | 500 | 100 | 2 | 0.02 | 1 | 0.01 |
| Basin.Swamp | 500 | 100 | 13 | 0.13 | 0 | 0 |
| Baygall | 500 | 100 | 0 | 0 | 2 | 0.02 |
| Lake.Bottom | 500 | 100 | 0 | 0 | 83 | 0.83 |
| Mesic.Hammock | 500 | 100 | 0 | 0 | 0 | 0 |
| Pine.Plantation | 500 | 100 | 0 | 0 | 91 | 0.91 |
| Sandhill | 500 | 100 | 0 | 0 | 100 | 1 |
| Sandhill.Upland.Lake | 500 | 100 | 0 | 0 | 99 | 0.99 |
| Succesional.Hardwood.Forest | 500 | 100 | 0 | 0 | 1 | 0.01 |
| Xeric.Hammock | 500 | 100 | 0 | 0 | 92 | 0.92 |
| Improved | 500 | 100 | 0 | 0 | 62 | 0.62 |
| Abandoned.Field.Pasture | 500 | 100 | 1 | 0.01 | 0 | 0 |
| Scrubby.Flatwoods | 500 | 100 | 0 | 0 | 100 | 1 |
| Clastic.Upland.Lake | 500 | 100 | 0 | 0 | 0 | 0 |

Table S25: Numbers and proportions of replicates showing significant phylogenetic patterns of PD_SES_ for chronograms of randomly sampled subsets for each community (Question 5). Results are separated into clustered and overdispersed patterns.

| Community | Subset | Number of replicates | Number Overdispersed | Proportion Overdispersed | Number Clustered | Proportion Clustered |
| --- | --- | --- | --- | --- | --- | --- |
| Basin.Marsh | 100 | 100 | 12 | 0.12 | 0 | 0 |
| Basin.Swamp | 100 | 100 | 62 | 0.62 | 0 | 0 |
| Baygall | 100 | 100 | 19 | 0.19 | 0 | 0 |
| Lake.Bottom | 100 | 100 | 6 | 0.06 | 0 | 0 |
| Mesic.Hammock | 100 | 100 | 30 | 0.3 | 0 | 0 |
| Pine.Plantation | 100 | 100 | 18 | 0.18 | 0 | 0 |
| Sandhill | 100 | 100 | 0 | 0 | 23 | 0.23 |
| Sandhill.Upland.Lake | 100 | 100 | 11 | 0.11 | 0 | 0 |
| Succesional.Hardwood.Forest | 100 | 100 | 11 | 0.11 | 0 | 0 |
| Xeric.Hammock | 100 | 100 | 5 | 0.05 | 3 | 0.03 |
| Improved | 100 | 100 | 0 | 0 | 2 | 0.02 |
| Abandoned.Field.Pasture | 100 | 100 | 7 | 0.07 | 0 | 0 |
| Scrubby.Flatwoods | 100 | 100 | 8 | 0.08 | 0 | 0 |
| Clastic.Upland.Lake | 100 | 100 | 2 | 0.02 | 0 | 0 |
| Basin.Marsh | 200 | 100 | 20 | 0.2 | 0 | 0 |
| Basin.Swamp | 200 | 100 | 72 | 0.72 | 0 | 0 |
| Baygall | 200 | 100 | 21 | 0.21 | 0 | 0 |
| Lake.Bottom | 200 | 100 | 3 | 0.03 | 2 | 0.02 |
| Mesic.Hammock | 200 | 100 | 36 | 0.36 | 0 | 0 |
| Pine.Plantation | 200 | 100 | 5 | 0.05 | 0 | 0 |
| Sandhill | 200 | 100 | 0 | 0 | 82 | 0.82 |
| Sandhill.Upland.Lake | 200 | 100 | 8 | 0.08 | 0 | 0 |
| Succesional.Hardwood.Forest | 200 | 100 | 16 | 0.16 | 0 | 0 |
| Xeric.Hammock | 200 | 100 | 1 | 0.01 | 4 | 0.04 |
| Improved | 200 | 100 | 0 | 0 | 4 | 0.04 |
| Abandoned.Field.Pasture | 200 | 100 | 15 | 0.15 | 0 | 0 |
| Scrubby.Flatwoods | 200 | 100 | 0 | 0 | 2 | 0.02 |
| Clastic.Upland.Lake | 200 | 100 | 2 | 0.02 | 0 | 0 |
| Basin.Marsh | 300 | 100 | 20 | 0.2 | 0 | 0 |
| Basin.Swamp | 300 | 100 | 88 | 0.88 | 0 | 0 |
| Baygall | 300 | 100 | 21 | 0.21 | 0 | 0 |
| Lake.Bottom | 300 | 100 | 1 | 0.01 | 2 | 0.02 |
| Mesic.Hammock | 300 | 100 | 31 | 0.31 | 0 | 0 |
| Pine.Plantation | 300 | 100 | 0 | 0 | 0 | 0 |
| Sandhill | 300 | 100 | 0 | 0 | 98 | 0.98 |
| Sandhill.Upland.Lake | 300 | 100 | 0 | 0 | 0 | 0 |
| Succesional.Hardwood.Forest | 300 | 100 | 3 | 0.03 | 0 | 0 |
| Xeric.Hammock | 300 | 100 | 0 | 0 | 4 | 0.04 |
| Improved | 300 | 100 | 0 | 0 | 11 | 0.11 |
| Abandoned.Field.Pasture | 300 | 100 | 8 | 0.08 | 0 | 0 |
| Scrubby.Flatwoods | 300 | 100 | 0 | 0 | 5 | 0.05 |
| Clastic.Upland.Lake | 300 | 100 | 0 | 0 | 0 | 0 |
| Basin.Marsh | 400 | 100 | 22 | 0.22 | 0 | 0 |
| Basin.Swamp | 400 | 100 | 99 | 0.99 | 0 | 0 |
| Baygall | 400 | 100 | 20 | 0.2 | 0 | 0 |
| Lake.Bottom | 400 | 100 | 0 | 0 | 5 | 0.05 |
| Mesic.Hammock | 400 | 100 | 16 | 0.16 | 0 | 0 |
| Pine.Plantation | 400 | 100 | 0 | 0 | 0 | 0 |
| Sandhill | 400 | 100 | 0 | 0 | 100 | 1 |
| Sandhill.Upland.Lake | 400 | 100 | 0 | 0 | 0 | 0 |
| Succesional.Hardwood.Forest | 400 | 100 | 4 | 0.04 | 0 | 0 |
| Xeric.Hammock | 400 | 100 | 0 | 0 | 4 | 0.04 |
| Improved | 400 | 100 | 0 | 0 | 28 | 0.28 |
| Abandoned.Field.Pasture | 400 | 100 | 7 | 0.07 | 0 | 0 |
| Scrubby.Flatwoods | 400 | 100 | 0 | 0 | 21 | 0.21 |
| Clastic.Upland.Lake | 400 | 100 | 1 | 0.01 | 0 | 0 |
| Basin.Marsh | 500 | 100 | 8 | 0.08 | 0 | 0 |
| Basin.Swamp | 500 | 100 | 100 | 1 | 0 | 0 |
| Baygall | 500 | 100 | 3 | 0.03 | 0 | 0 |
| Lake.Bottom | 500 | 100 | 0 | 0 | 10 | 0.1 |
| Mesic.Hammock | 500 | 100 | 2 | 0.02 | 0 | 0 |
| Pine.Plantation | 500 | 100 | 0 | 0 | 0 | 0 |
| Sandhill | 500 | 100 | 0 | 0 | 100 | 1 |
| Sandhill.Upland.Lake | 500 | 100 | 0 | 0 | 0 | 0 |
| Succesional.Hardwood.Forest | 500 | 100 | 0 | 0 | 0 | 0 |
| Xeric.Hammock | 500 | 100 | 0 | 0 | 18 | 0.18 |
| Improved | 500 | 100 | 0 | 0 | 63 | 0.63 |
| Abandoned.Field.Pasture | 500 | 100 | 0 | 0 | 0 | 0 |
| Scrubby.Flatwoods | 500 | 100 | 0 | 0 | 63 | 0.63 |
| Clastic.Upland.Lake | 500 | 100 | 0 | 0 | 0 | 0 |

Table S26: Species included in phylogenetic analysis with voucher information and GenBank accession numbers

| **Taxon** | **Voucher** | ***matK* GenBank Accession** | ***rbcL* GenBank Accession** |
| --- | --- | --- | --- |
| **Acalypha gracilens** | FLAS: *Majure 4720* |  |  |
| **Acer rubrum** | FLAS: *Majure 4735* | MH551750 | KY626932 |
| **Acmella oppositifolia var. repens** | FLAS: *Whitten 4046* | MH552314 | KY627490 |
| **Aeschynomene viscidula** | FLAS: *Whitten 3915* | MH552187 | KY627366 |
| **Agalinis fasciculata** | FLAS: *Whitten 4010* | MH552278 | KY627454 |
| **Agalinis plukenetii** | FLAS: *Whitten 4032* | MH552300 | KY627476 |
| **Agalinis setacea** | FLAS: *Whitten 3997* | MH552266 | KY627441 |
| **Ageratina jucunda** | FLAS: *Majure 4518* | MH551561 | KY626722 |
| **Agrostis hyemalis** | FLAS: *Majure 5038* | MH551968 | KY627151 |
| **Albizia julibrissin** | FLAS: *Majure 5113* | MH552032 | KY627206 |
| **Alysicarpus ovalifolius** | FLAS: *Whitten 4042* | MH552310 | KY627486 |
| **Ambrosia artemisiifolia** | FLAS: *Majure 4715* | MH551731 | KY626913 |
| **Amorpha herbacea** | FLAS: *Majure 4942* | MH551886 | KY627076 |
| **Amphicarpum muhlenbergianum** | FLAS: *Whitten 4021a* | MH552289 | KY627465 |
| **Amsonia ciliata** | FLAS: *Majure 4941* | MH551885 | KY627075 |
| **Andropogon brachystachyus** | FLAS: *Majure 4561* | MH551598 | KY626765 |
| **Andropogon floridanus** | FLAS: *Whitten 3996* | MH552265 | KY627440 |
| **Andropogon glomeratus var. glomeratus** | FLAS: *Majure 4652* | MH551676 | KY626853 |
| **Andropogon gyrans var. stenophyllus** | FLAS: *Majure 4554* | MH551591 | KY626758 |
| **Andropogon ternarius** | FLAS: *Whitten 4028* | MH552296 | KY627472 |
| **Andropogon tracyi** | FLAS: *Majure 4547* | MH551585 | KY626751 |
| **Andropogon virginicus var. virginicus** | FLAS: *Majure 4687* | MH551706 | KY626886 |
| **Anthaenantia villosa** | FLAS: *Majure 4660* | MH551682 | KY626860 |
| **Ardisia crenata** | FLAS: *Majure 4732* | MH551747 | KY626929 |
| **Arenaria serpyllifolia** | FLAS: *Majure 4830* | MH551807 | KY626993 |
| **Aristida condensata** | FLAS: *Majure 4523* | MH551566 | KY626727 |
| **Aristida gyrans** | FLAS: *Majure 4532* | MH551573 | KY626736 |
| **Aristida mohrii** | FLAS: *Majure 4526* | MH551569 | KY626730 |
| **Aristida purpurascens var. purpurascens** | FLAS: *Majure 4534* | MH551574 | KY626738 |
| **Aristida spiciformis** | FLAS: *Whitten 3968* | MH552238 | KY627413 |
| **Aristida stricta** | FLAS: *Majure 4531* | MH551572 | KY626735 |
| **Aristolochia serpentaria** | FLAS: *Majure 5153* | MH552068 | KY627244 |
| **Arnoglossum floridanum** | FLAS: *Majure 5114* | MH552033 | KY627207 |
| **Asclepias humistrata** | FLAS: *Majure 4936* | MH551880 | KY627070 |
| **Asclepias tomentosa** | FLAS: *Majure 5116* | MH552035 | KY627209 |
| **Asclepias tuberosa** | FLAS: *Majure 5099* | MH552027 | KY627201 |
| **Asclepias verticillata** | FLAS: *Whitten 3917* | MH552189 | KY627368 |
| **Asemeia violacea** | FLAS: *Majure 5058* | MH551988 | KY627167 |
| **Asimina angustifolia** | FLAS: *Majure 4989* | MH551931 | KY627116 |
| **Asimina incana** | FLAS: *Majure 4887* | MH551844 | KY627032 |
| **Asimina parviflora** | FLAS: *Majure 4737* | MH551752 | KY626934 |
| **Asimina pygmaea** | FLAS: *Majure 4970* | MH551912 | KY627100 |
| **Asplenium platyneuron** | FLAS: *Majure 5154* |  | KY627245 |
| **Astragalus obcordatus** | FLAS: *Majure 4842* | MH551816 | KY627004 |
| **Axonopus fissifolius** | FLAS: *Majure 5052* | MH551982 | KY627162 |
| **Axonopus furcatus** | FLAS: *Majure 4572* |  | KY626775 |
| **Baccharis halimifolia** | FLAS: *Majure 4633* | MH551658 | KY626834 |
| **Balduina angustifolia** | FLAS: *Majure 4508* | MH551551 | KY626712 |
| **Bambusa multiplex** | FLAS: *Majure 4998* | MH551940 | KY627124 |
| **Bejaria racemosa** | FLAS: *Majure 5272* | MH552161 | KY627340 |
| **Berlandiera subacaulis** | FLAS: *Majure 4882* | MH551840 | KY627027 |
| **Bidens alba** | FLAS: *Majure 4915* | MH551871 | KY627060 |
| **Bidens mitis** | FLAS: *Majure 4641* | MH551665 | KY626842 |
| **Bignonia capreolata** | FLAS: *Majure 5191* | MH552096 | KY627274 |
| **Boerhavia diffusa** | FLAS: *Whitten 4048* | MH552316 | KY627492 |
| **Boltonia diffusa** | FLAS: *Whitten 3933* | MH552204 | KY627383 |
| **Bothriochloa pertusa** | FLAS: *Whitten 4039* | MH552307 | KY627483 |
| **Botrychium biternatum** | FLAS: *Majure 5152* |  | KY627243 |
| **Brasenia schreberi** | FLAS: *Majure 5258* | MH552147 | KY627326 |
| **Bromus catharticus** | FLAS: *Majure 5031* | MH551962 | KY627144 |
| **Buchnera americana** | FLAS: *Majure 5083* | MH552011 | KY627188 |
| **Bulbostylis barbata** | FLAS: *Majure 5239* | MH552129 | KY627307 |
| **Bulbostylis ciliatifolia** | FLAS: *Whitten 3929* | MH552200 | KY627380 |
| **Bulbostylis stenophylla** | FLAS: *Majure 5261* | MH552150 | KY627329 |
| **Bulbostylis warei** | FLAS: *Majure 4533* |  | KY626737 |
| **Butia capitata** | FLAS: *Majure 4846* | MH551819 | KY627008 |
| **Buxus microphylla** | FLAS: *Majure 4843* | MH551817 | KY627005 |
| **Callicarpa americana** | FLAS: *Majure 4691* | MH551710 | KY626890 |
| **Callisia graminea** | FLAS: *Majure 4898* | MH551855 | KY627043 |
| **Calyptocarpus vialis** | FLAS: *Whitten 4055* | MH552323 | KY627499 |
| **Camellia sasanqua** | FLAS: *Majure 4919* | MH551875 | KY627064 |
| **Campsis radicans** | FLAS: *Majure 4997* | MH551939 | KY627123 |
| **Cardamine hirsuta** | FLAS: *Whitten 4099* | MH552367 | KY627543 |
| **Carex atlantica ssp. capillacea** | FLAS: *Majure 4957* | MH551901 | KY627091 |
| **Carex dasycarpa** | FLAS: *Majure 4899* |  | KY627044 |
| **Carex elliottii** | FLAS: *Majure 5143* | MH552061 | KY627234 |
| **Carex fissa var. aristata** | FLAS: *Majure 5027* | MH551958 | KY627142 |
| **Carex floridana** | FLAS: *Majure 4844* | MH551818 | KY627006 |
| **Carex longii** | FLAS: *Majure 5062* | MH551992 | KY627171 |
| **Carphephorus corymbosus** | FLAS: *Whitten 3961* | MH552232 | KY627406 |
| **Cartrema americanum** | FLAS: *Majure 4900* | MH551856 | KY627045 |
| **Carya glabra** | FLAS: *Majure 5213* | MH552117 | KY627295 |
| **Carya illinoensis** | FLAS: *Majure 5046* | MH551976 | KY627158 |
| **Castanea mollissima** | FLAS: *Majure 4999* | MH551941 | KY627125 |
| **Castanea pumila** | FLAS: *Majure 5158* | MH552071 | KY627249 |
| **Ceanothus microphyllus** | FLAS: *Majure 5292* | MH552179 | KY627358 |
| **Cenchrus gracillimus** | FLAS: *Majure 4580* | MH551614 | KY626783 |
| **Cenchrus spinifex** | FLAS: *Majure 5112* | MH552031 | KY627205 |
| **Centella asiatica** | FLAS: *Majure 5174* | MH552079 | KY627257 |
| **Centrosema virginianum** | FLAS: *Majure 5245* | MH552135 | KY627313 |
| **Cephalanthus occidentalis** | FLAS: *Majure 5180* | MH552085 | KY627263 |
| **Cerastium glomeratum** | FLAS: *Majure 4914* | MH551870 | KY627059 |
| **Ceratiola ericoides** | FLAS: *Majure 4603* | MH551632 | KY626804 |
| **Chamaecrista fasciculata** | FLAS: *Majure 5274* | MH552163 | KY627342 |
| **Chamaecrista nictitans var. aspera** | FLAS: *Majure 4517* | MH551560 | KY626721 |
| **Chamaesyce hirta** | FLAS: *Whitten 4040* | MH552308 | KY627484 |
| **Chamaesyce _hyssopifolia_** | FLAS: *Whitten 4014* | MH552282 | KY627458 |
| **Chapmannia floridana** | FLAS: *Majure 5111* | MH552030 | KY627204 |
| **Chasmanthium sessiliflorum** | FLAS: *Majure 4773* | MH551786 |  |
| **Chionanthus virginicus** | FLAS: *Majure 4765* | MH551779 | KY626963 |
| **Chrysopsis gossypina** | FLAS: *Whitten 3954* | MH552225 | KY627400 |
| **Chrysopsis mariana** | FLAS: *Whitten 3988* | MH552258 | KY627433 |
| **Cinnamomum camphora** | FLAS: *Majure 4733* | MH551748 | KY626930 |
| **Cirsium horridulum** | FLAS: *Majure 4893* | MH551850 | KY627038 |
| **Cirsium nuttalii** | FLAS: *Majure 5278* | MH552165 | KY627344 |
| **Citrus X aurantium** | FLAS: *Majure 4728* | MH551743 | KY626925 |
| **Cladium jamaicense** | FLAS: *Majure 5188* | MH552093 | KY627271 |
| **Clematis reticulata** | FLAS: *Majure 5093* | MH552021 | KY627196 |
| **Clitoria mariana** | FLAS: *Majure 5270* | MH552159 | KY627338 |
| **Cnidoscolus stimulosus** | FLAS: *Majure 4885* | MH551842 | KY627030 |
| **Coelorachis tuberculosa** | FLAS: *Majure 4587* | MH551619 | KY626790 |
| **Commelina diffusa** | FLAS: *Whitten 4016* | MH552284 | KY627460 |
| **Commelina erecta** | FLAS: *Majure 4563* | MH551600 | KY626767 |
| **Conyza canadensis** | FLAS: *Majure 4567* | MH551603 | KY626770 |
| **Coreopsis tinctoria** | FLAS: *Majure 5033* | MH551963 | KY627146 |
| **Cornus florida** | FLAS: *Majure 4920* | MH551876 | KY627065 |
| **Cortaderia selloana** | FLAS: *Majure 5061* | MH551991 | KY627170 |
| **Corydalis micrantha subsp. australis** | FLAS: *Majure 4838* | MH551812 | KY627000 |
| **Crataegus michauxii** | FLAS: *Majure 4908* | MH551864 | KY627053 |
| **Crinum moorei X ornatum** | FLAS: *Majure 5235* | MH552125 | KY627303 |
| **Crocanthemum carolinianum** | FLAS: *Majure 4878* | MH551836 | KY627025 |
| **Crocanthemum corymbosum** | FLAS: *Majure 4529* |  | KY626733 |
| **Croptilon divaricatum** | FLAS: *Majure 4701* | MH551717 | KY626899 |
| **Crotalaria lanceolata** | FLAS: *Whitten 3975* | MH552245 | KY627420 |
| **Crotalaria pallida var. obovata** | FLAS: *Whitten 3984* | MH552254 | KY627429 |
| **Crotalaria rotundifolia** | FLAS: *Majure 4886* | MH551843 | KY627031 |
| **Crotalaria spectabilis** | FLAS: *Whitten 3991* | MH552260 |  |
| **Croton argyranthemus** | FLAS: *Majure 4550* |  | KY626754 |
| **Croton glandulosus var. glandulosus** | FLAS: *Majure 4723* | MH551738 | KY626920 |
| **Croton michauxii** | FLAS: *Majure 4688* | MH551707 | KY626887 |
| **Ctenium floridanum** | FLAS: *Majure 4859* | MH551832 | KY627021 |
| **Cucumis anguria** | FLAS: *Whitten 4058* | MH552326 | KY627502 |
| **Cuscuta pentagona** | FLAS: *Majure 5082* |  | KY627187 |
| **Cycas revoluta** | FLAS: *Majure 4827* |  | KY626990 |
| **Cyclospermum leptophyllum** | FLAS: *Majure 4949* | MH551893 | KY627083 |
| **Cynodon dactylon** | FLAS: *Majure 5037* | MH551967 | KY627150 |
| **Cyperus compressus** | FLAS: *Majure 5169* | MH552074 | KY627252 |
| **Cyperus croceus** | FLAS: *Majure 4747* | MH551762 | KY626944 |
| **Cyperus erythrorhizos** | FLAS: *Whitten 3947* | MH552218 | KY627397 |
| **Cyperus filiculmis** | FLAS: *Majure 5078* | MH552007 | KY627183 |
| **Cyperus haspan** | FLAS: *Majure 5209* | MH552113 | KY627291 |
| **Cyperus lecontei** | FLAS: *Whitten 4021b* | MH552290 | KY627466 |
| **Cyperus odoratus** | FLAS: *Whitten 3945* | MH552216 | KY627395 |
| **Cyperus plukenetii** | FLAS: *Majure 4696* | MH551714 | KY626895 |
| **Cyperus polystachyos** | FLAS: *Majure 5190* | MH552095 | KY627273 |
| **Cyperus retrorsus** | FLAS: *Majure 4672* |  | KY626872 |
| **Cyperus surinamensis** | FLAS: *Majure 5036* | MH551966 | KY627149 |
| **Cyperus tetragonus** | FLAS: *Majure 4745* | MH551760 | KY626942 |
| **Cyrilla racemiflora** | FLAS: *Majure 4776* | MH551789 | KY626972 |
| **Dactyloctenium aegyptium** | FLAS: *Majure 4742* | MH551757 | KY626939 |
| **Dalea feayi** | FLAS: *Whitten 3936* | MH552207 | KY627386 |
| **Dalea pinnata** | FLAS: *Whitten 3982* | MH552252 | KY627427 |
| **Decodon verticillatus** | FLAS: *Majure 4706* | MH551722 | KY626904 |
| **Desmodium floridanum** | FLAS: *Majure 5129* | MH552047 | KY627221 |
| **Desmodium incanum** | FLAS: *Whitten 4011* | MH552279 | KY627455 |
| **Desmodium strictum** | FLAS: *Whitten 3998* | MH552267 | KY627442 |
| **Desmodium tortuosum** | FLAS: *Whitten 4013* | MH552281 | KY627457 |
| **Desmodium triflorum** | FLAS: *Majure 4693* | MH551711 | KY626892 |
| **Desmodium viridiflorum** | FLAS: *Whitten 3964* | MH552235 | KY627409 |
| **Dichanthelium aciculare subsp. aciculare** | FLAS: *Majure 5087* | MH552015 | KY627191 |
| **Dichanthelium commutatum ssp. commutatum** | FLAS: *Majure 4948* | MH551892 | KY627082 |
| **Dichanthelium erectifolium** | FLAS: *Majure 5197* | MH552101 | KY627280 |
| **Dichanthelium laxiflorum** | FLAS: *Majure 5023* | MH551954 | KY627138 |
| **Dichanthelium oligosanthes subsp. oligosanthes** | FLAS: *Majure 4697* | MH551715 | KY626896 |
| **Dichanthelium ovale** | FLAS: *Majure 5132* | MH552050 | KY627224 |
| **Dichanthelium portoricense ssp. patulum** | FLAS: *Majure 5204* | MH552108 | KY627286 |
| **Dichanthelium strigosum var. glabrescens** | FLAS: *Majure 5243* | MH552133 | KY627311 |
| **Dichanthelium tenue** | FLAS: *Majure 4952* | MH551896 | KY627086 |
| **Dichanthelium webberianum** | FLAS: *Majure 5293* | MH552180 | KY627359 |
| **Dichanthelium wrightianum** | FLAS: *Majure 5247* | MH552137 | KY627315 |
| **Dichondra carolinensis** | FLAS: *Majure 4828* | MH551805 | KY626991 |
| **Digitaria bicornis** | FLAS: *Majure 5172* | MH552077 | KY627255 |
| **Digitaria cognata** | FLAS: *Majure 5266* | MH552155 | KY627334 |
| **Digitaria filiformis var. villosa** | FLAS: *Majure 4512* | MH551555 | KY626716 |
| **Digitaria longiflora** | FLAS: *Majure 4557* | MH551594 | KY626761 |
| **Diodia teres** | FLAS: *Majure 4556* | MH551593 | KY626760 |
| **Diospyros virginiana** | FLAS: *Majure 4565* | MH551602 | KY626768 |
| **Dolichandra unguis-cati** | FLAS: *Majure 4992* | MH551934 | KY627118 |
| **Drosera brevifolia** | FLAS: *Whitten 3937* | MH552208 | KY627387 |
| **Drosera capillaris** | FLAS: *Majure 5282* | MH552169 | KY627348 |
| **Drymaria cordata** | FLAS: *Majure 5025* | MH551956 | KY627140 |
| **Dulichium arundinaceum** | FLAS: *Majure 5250* | MH552139 | KY627318 |
| **Dyschoriste oblongifolia** | FLAS: *Majure 4975* | MH551917 | KY627104 |
| **Dysphania ambrosioides** | FLAS: *Whitten 4045* | MH552313 | KY627489 |
| **Echinochloa colonum** | FLAS: *Majure 5273* | MH552162 | KY627341 |
| **Eleocharis baldwinii** | FLAS: *Majure 4621* | MH551649 | KY626822 |
| **Eleocharis vivipara** | FLAS: *Majure 5010* | MH551951 | KY627135 |
| **Elephantopus elatus** | FLAS: *Whitten 3960* | MH552231 | KY627405 |
| **Eragrostis elliottii** | FLAS: *Whitten 3973* | MH552243 | KY627418 |
| **Eragrostis minor** | FLAS: *Majure 4767* | MH551781 | KY626965 |
| **Erechtites hieraciifolius var. hieraciifolius** | FLAS: *Majure 4618* | MH551646 | KY626819 |
| **Eremochloa ophiuroides** | FLAS: *Whitten 3994* | MH552263 | KY627438 |
| **Erigeron quercifolius** | FLAS: *Majure 4924* | MH551879 | KY627069 |
| **Erigeron vernus** | FLAS: *Majure 5240* | MH552130 | KY627308 |
| **Eriogonum tomentosum** | FLAS: *Majure 4506* | MH551549 | KY626710 |
| **Eryngium aromaticum** | FLAS: *Majure 4507* | MH551550 | KY626711 |
| **Eryngium baldwinii** | FLAS: *Majure 5053* | MH551983 | KY627163 |
| **Eryngium yuccifolium** | FLAS: *Majure 5290* | MH552177 | KY627356 |
| **Erythrina herbacea** | FLAS: *Majure 5002* | MH551944 | KY627128 |
| **Eubotrys racemosa** | FLAS: *Majure 4954* | MH551898 | KY627088 |
| **Euonymus americanus** | FLAS: *Majure 5097* | MH552025 | KY627200 |
| **Eupatorium album** | FLAS: *Whitten 3987* | MH552257 | KY627432 |
| **Eupatorium capillifolium** | FLAS: *Whitten 4034* | MH552302 | KY627478 |
| **Eupatorium compositifolium** | FLAS: *Majure 4522* | MH551565 | KY626726 |
| **Eupatorium leptophyllum** | FLAS: *Whitten 4006* | MH552275 | KY627450 |
| **Eupatorium mohrii** | FLAS: *Whitten 3966* | MH552236 | KY627411 |
| **Euphorbia prostrata** | FLAS: *Majure 4768* | MH551782 | KY626966 |
| **Eustachys neglecta** | FLAS: *Majure 5179* | MH552084 | KY627262 |
| **Eustachys petraea** | FLAS: *Majure 5045* | MH551975 | KY627157 |
| **Euthamia caroliniana** | FLAS: *Majure 4600* | MH551629 | KY626802 |
| **Facelis retusa** | FLAS: *Majure 5028* | MH551959 |  |
| **Ficus pumila** | FLAS: *Majure 4852* | MH551825 | KY627014 |
| **Fimbristylis caroliniana** | FLAS: *Majure 5216* | MH552120 | KY627298 |
| **Froelichia floridana** | FLAS: *Majure 4515* | MH551558 | KY626719 |
| **Fuirena pumila** | FLAS: *Whitten 3927* | MH552198 | KY627378 |
| **Fuirena scirpoidea** | FLAS: *Majure 4897* | MH551854 | KY627042 |
| **Galactia elliottii** | FLAS: *Majure 5193* | MH552098 | KY627276 |
| **Galactia erecta** | FLAS: *Majure 5254* | MH552143 | KY627322 |
| **Galactia mollis** | FLAS: *Majure 5068* | MH551997 | KY627176 |
| **Galactia regularis** | FLAS: *Majure 5146* | MH552063 | KY627237 |
| **Galium hispidulum** | FLAS: *Majure 4721* | MH551736 | KY626918 |
| **Galium pilosum** | FLAS: *Majure 5253* | MH552142 | KY627321 |
| **Gamochaeta antillana** | FLAS: *Majure 5030* | MH551961 | KY627143 |
| **Gamochaeta purpurea** | FLAS: *Majure 4876* | MH551834 | KY627023 |
| **Garberia heterophylla** | FLAS: *Whitten 3934* | MH552205 | KY627384 |
| **Gaylussacia dumosa** | FLAS: *Majure 4779* | MH551791 | KY626975 |
| **Gaylussacia nana** | FLAS: *Majure 4650* | MH551674 | KY626851 |
| **Gaylussacia tomentosa** | FLAS: *Majure 4962* | MH551905 | KY627093 |
| **Gelsemium sempervirens** | FLAS: *Majure 4824* | MH551802 | KY626987 |
| **Geranium carolinianum** | FLAS: *Majure 4921* |  | KY627066 |
| **Gomphrena serrata** | FLAS: *Majure 5022* | MH551953 | KY627137 |
| **Gordonia lasianthus** | FLAS: *Majure 4922* | MH551877 | KY627067 |
| **Gratiola hispida** | FLAS: *Majure 4671* | MH551693 | KY626871 |
| **Gymnopogon ambiguus** | FLAS: *Majure 4511* | MH551554 | KY626715 |
| **Habenaria odontopetala** | FLAS: *Huey s.n.* | MH551798 | KY626983 |
| **Habenaria repens** | FLAS: *Majure 5183* | MH552088 | KY627266 |
| **Helianthus radula** | FLAS: *Whitten 3944* | MH552215 | KY627394 |
| **Heliotropium amplexicaule** | FLAS: *Majure 5040* | MH551970 | KY627153 |
| **Heterotheca subaxillaris** | FLAS: *Whitten 3978* | MH552248 | KY627423 |
| **Hieracium** | FLAS: *Whitten 3935* | MH552206 | KY627385 |
| **Hieracium megacephalon** | FLAS: *Majure 4761* | MH551775 | KY626959 |
| **Houstonia procumbens** | FLAS: *Majure 4749* | MH551764 | KY626946 |
| **Hydrocotyle umbellata** | FLAS: *Majure 5182* | MH552087 | KY627265 |
| **Hydrocotyle verticillata** | FLAS: *Majure 5148* | MH552064 | KY627239 |
| **Hypericum brachyphyllum** | FLAS: *Majure 4591* |  | KY626793 |
| **Hypericum fasciculatum** | FLAS: *Majure 4651* | MH551675 | KY626852 |
| **Hypericum gentianoides** | FLAS: *Whitten 3923* | MH552194 | KY627374 |
| **Hypericum hypericoides** | FLAS: *Majure 4607* |  | KY626808 |
| **Hypericum mutilum** | FLAS: *Majure 5208* | MH552112 | KY627290 |
| **Hypericum tetrapetalum** | FLAS: *Majure 4589* |  | KY626792 |
| **Hypochaeris chillensis** | FLAS: *Majure 5024* | MH551955 | KY627139 |
| **Hyptis mutabilis** | FLAS: *Majure 5233* | MH552123 | KY627301 |
| **Ilex ambigua** | FLAS: *Majure 4986* | MH551928 | KY627114 |
| **Ilex cassine var. cassine** | FLAS: *Majure 4627* | MH551653 | KY626828 |
| **Ilex coriacea** | FLAS: *Majure 4860* | MH551833 | KY627022 |
| **Ilex cornuta** | FLAS: *Majure 4918* | MH551874 | KY627063 |
| **Ilex glabra** | FLAS: *Majure 4577* | MH551611 | KY626780 |
| **Ilex opaca** | FLAS: *Majure 4775* | MH551788 | KY626971 |
| **Imperata cylindrica** | FLAS: *Majure 5283* | MH552170 | KY627349 |
| **Indigofera caroliniana** | FLAS: *Majure 5207* | MH552111 | KY627289 |
| **Indigofera spicata** | FLAS: *Majure 5140* | MH552058 | KY627231 |
| **Indigofera suffruticosa** | FLAS: *Whitten 3993* | MH552262 | KY627437 |
| **Ipomoea cordatotriloba** | FLAS: *Whitten 3976* | MH552246 | KY627421 |
| **Ipomoea hederifolia** | FLAS: *Whitten 4053* | MH552321 | KY627497 |
| **Ipomoea triloba** | FLAS: *Whitten 4036* | MH552304 | KY627480 |
| **Jacquemontia tamnifolia** | FLAS: *Whitten 4037* | MH552305 | KY627481 |
| **Juncus dichotomus** | FLAS: *Majure 4717* | MH551733 | KY626915 |
| **Juncus effusus** | FLAS: *Majure 4729* | MH551744 | KY626926 |
| **Juncus elliottii** | FLAS: *Majure 5176* | MH552081 | KY627259 |
| **Juncus marginatus** | FLAS: *Majure 5055* | MH551985 | KY627165 |
| **Juncus repens** | FLAS: *Majure 4622* | MH551650 | KY626823 |
| **Juncus scirpoides** | FLAS: *Majure 4786* | MH551773 | KY626956 |
| **Juniperus virginiana var. silicola** | FLAS: *Majure 5032* |  | KY627145 |
| **Koelreuteria elegans subsp. formosana** | FLAS: *Majure 4995* | MH551937 | KY627121 |
| **Krameria lanceolata** | FLAS: *Majure 5079* | MH552008 | KY627184 |
| **Krigia virginica** | FLAS: *Majure 4847* | MH551820 | KY627009 |
| **Kummerowia striata** | FLAS: *Whitten 4043* | MH552311 | KY627487 |
| **Lachnanthes caroliniana** | FLAS: *Majure 4647* | MH551671 | KY626848 |
| **Lachnocaulon anceps** | FLAS: *Majure 5064* | MH551994 | KY627173 |
| **Lachnocaulon engleri** | FLAS: *Majure 5284* | MH552171 | KY627350 |
| **Lachnocaulon minus** | FLAS: *Majure 5260* | MH552149 | KY627328 |
| **Lactuca graminifolia** | FLAS: *Majure 5088* | MH552016 | KY627192 |
| **Lantana camara** | FLAS: *Whitten 4054* | MH552322 | KY627498 |
| **Lechea minor** | FLAS: *Majure 4947* | MH551891 | KY627081 |
| **Lechea mucronata** | FLAS: *Majure 4753* | MH551767 | KY626950 |
| **Lechea sessiliflora** | FLAS: *Majure 4754* | MH551768 | KY626951 |
| **Leersia hexandra** | FLAS: *Majure 4586* | MH551618 | KY626789 |
| **Lemna aequinoctialis** | FLAS: *Majure 4823* | MH551801 | KY626986 |
| **Lemna valdiviana** | FLAS: *Majure 5215* | MH552119 | KY627297 |
| **Lespedeza hirta** | FLAS: *Majure 4763* | MH551777 | KY626961 |
| **Lespedeza procumbens** | FLAS: *Majure 5131* | MH552049 | KY627223 |
| **Liatris gracilis** | FLAS: *Whitten 4023* | MH552291 | KY627467 |
| **Liatris pauciflora** | FLAS: *Whitten 3957* | MH552228 | KY627402 |
| **Liatris tenuifolia** | FLAS: *Majure 4542* | MH551581 | KY626746 |
| **Licania michauxii** | FLAS: *Majure 5201* | MH552105 | KY627283 |
| **Limnobium spongia** | FLAS: *Majure 5177* | MH552082 | KY627260 |
| **Linaria canadensis** | FLAS: *Majure 4911* | MH551867 | KY627056 |
| **Linaria floridana** | FLAS: *Majure 4907* | MH551863 | KY627052 |
| **Lindernia crustacea** | FLAS: *Majure 5263* | MH552152 | KY627331 |
| **Liquidambar styraciflua** | FLAS: *Majure 4711* | MH551727 | KY626909 |
| **Lobelia paludosa** | FLAS: *Majure 5157* | MH552070 | KY627248 |
| **Lolium multiflorum** | FLAS: *Majure 5048* | MH551978 | KY627160 |
| **Ludwigia alternifolia** | FLAS: *Majure 5232* | MH552122 | KY627300 |
| **Ludwigia leptocarpa** | FLAS: *Majure 4703* | MH551719 | KY626901 |
| **Ludwigia palustris** | FLAS: *Majure 4716* | MH551732 | KY626914 |
| **Ludwigia suffruticosa** | FLAS: *Whitten 3962* | MH552233 | KY627407 |
| **Lupinus diffusus** | FLAS: *Majure 4822* | MH551800 | KY626985 |
| **Lycopodiella appressa** | FLAS: *Majure 4582* |  | KY626785 |
| **Lycopus rubellus** | FLAS: *Majure 4617* | MH551645 | KY626818 |
| **Lygodesmia aphylla** | FLAS: *Majure 5069* | MH551998 |  |
| **Lyonia ferruginea** | FLAS: *Majure 4856* | MH551829 | KY627018 |
| **Lyonia fruticosa** | FLAS: *Majure 4771* | MH551784 | KY626968 |
| **Lyonia lucida** | FLAS: *Majure 4634* | MH551659 | KY626835 |
| **Lyonia mariana** | FLAS: *Majure 4950* | MH551894 | KY627084 |
| **Magnolia grandiflora** | FLAS: *Majure 4734* | MH551749 | KY626931 |
| **Magnolia virginiana** | FLAS: *Majure 4639* | MH551663 | KY626840 |
| **Matelea pubiflora** | FLAS: *Majure 5244* | MH552134 | KY627312 |
| **Mayaca fluviatilis** | FLAS: *Majure 5217* | MH552121 | KY627299 |
| **Medicago lupulina** | FLAS: *Majure 4850* | MH551823 | KY627012 |
| **Melia azedarach** | FLAS: *Majure 5149* | MH552065 | KY627240 |
| **Melilotus albus** | FLAS: *Majure 5160* | MH552073 | KY627251 |
| **Melinis repens** | FLAS: *Majure 4783* | MH551795 | KY626979 |
| **Melothria pendula** | FLAS: *Majure 5156* | MH552069 | KY627247 |
| **Mimosa quadrivalvis** | FLAS: *Majure 5211* | MH552115 | KY627293 |
| **Mitchella repens** | FLAS: *Majure 4654* | MH551678 | KY626855 |
| **Mitracarpus hirtus** | FLAS: *Whitten 4041* | MH552309 | KY627485 |
| **Mollugo verticillata** | FLAS: *Majure 5142* | MH552060 | KY627233 |
| **Monarda punctata** | FLAS: *Whitten 3986* | MH552256 | KY627431 |
| **Morus rubra** | FLAS: *Majure 5089* | MH552017 | KY627193 |
| **Murdannia nudiflora** | FLAS: *Majure 4722* | MH551737 | KY626919 |
| **Myrica cerifera var. cerifera** | FLAS: *Majure 4636* | MH551660 | KY626837 |
| **Nephrolepis cordifolia** | FLAS: *Majure 5248* |  | KY627316 |
| **Nuphar advena** | FLAS: *Majure 5186* | MH552091 | KY627269 |
| **Nymphaea odorata** | FLAS: *Majure 5185* | MH552090 | KY627268 |
| **Nyssa sylvatica var. biflora** | FLAS: *Majure 4637* | MH551661 | KY626838 |
| **Oenothera biennis** | FLAS: *Whitten 4038* | MH552306 | KY627482 |
| **Oenothera laciniata** | FLAS: *Majure 5029* | MH551960 |  |
| **Oenothera simulans** | FLAS: *Majure 5042* | MH551972 | KY627154 |
| **Oldenlandia corymbosa** | FLAS: *Majure 4675* | MH551696 | KY626875 |
| **Oldenlandia uniflora** | FLAS: *Majure 4573* | MH551608 | KY626776 |
| **Ophioglossum petiolatum** | FLAS: *Majure 4835* |  | KY626997 |
| **Oplismenus hirtellus subsp. setarius** | FLAS: *Majure 4718* | MH551734 | KY626916 |
| **Opuntia mesacantha subsp. lata** | FLAS: *Majure 5000* | MH551942 | KY627126 |
| **Orbexilum lupinellus** | FLAS: *Majure 5139* | MH552057 | KY627230 |
| **Osmunda regalis** | FLAS: *Majure 5144* |  | KY627235 |
| **Osmundastrum cinnamomeum** | FLAS: *Majure 4623* |  | KY626824 |
| **Oxalis corniculata** | FLAS: *Majure 4746* | MH551761 | KY626943 |
| **Oxalis debilis** | FLAS: *Majure 5214* | MH552118 | KY627296 |
| **Palafoxia integrifolia** | FLAS: *Whitten 4008* | MH552276 | KY627452 |
| **Panicum anceps subsp. rhizomatum** | FLAS: *Majure 4624* | MH551651 | KY626825 |
| **Panicum hemitomon** | FLAS: *Majure 5125* | MH552043 | KY627217 |
| **Panicum repens** | FLAS: *Majure 5236* | MH552126 | KY627304 |
| **Panicum verrucosum** | FLAS: *Majure 4595* | MH551624 | KY626797 |
| **Paronychia americana** | FLAS: *Majure 5076* | MH552005 | KY627181 |
| **Paronychia baldwinii** | FLAS: *Majure 4714* | MH551730 | KY626912 |
| **Paronychia patula** | FLAS: *Majure 4597* | MH551626 | KY626799 |
| **Parthenocissus quinquefolia** | FLAS: *Majure 4906* | MH551862 | KY627051 |
| **Paspalum notatum** | FLAS: *Majure 5021* | MH551952 | KY627136 |
| **Paspalum setaceum var. ciliatifolium** | FLAS: *Majure 4553* | MH551590 | KY626757 |
| **Paspalum urvillei** | FLAS: *Majure 5115* | MH552034 | KY627208 |
| **Passiflora incarnata** | FLAS: *Whitten 4063* | MH552331 | KY627507 |
| **Pectis prostrata** | FLAS: *Whitten 4056* | MH552324 | KY627500 |
| **Pediomelum canescens** | FLAS: *Majure 5118* | MH552037 | KY627211 |
| **Peltandra virginica** | FLAS: *Majure 4960* | MH551903 |  |
| **Pennisetum purpureum** | FLAS: *Whitten 4025* | MH552293 | KY627469 |
| **Penstemon multiflorus** | FLAS: *Majure 4689* | MH551708 | KY626888 |
| **Persea palustris** | FLAS: *Majure 4629* | MH551655 | KY626830 |
| **Persicaria glabra** | FLAS: *Majure 5178* | MH552083 | KY627261 |
| **Persicaria hydropiperoides** | FLAS: *Majure 5189* | MH552094 | KY627272 |
| **Phlebodium aureum** | FLAS: *Majure 4676* |  | KY626876 |
| **Phlox drummondii** | FLAS: *Majure 5141* | MH552059 | KY627232 |
| **Phlox nivalis** | FLAS: *Majure 4895* | MH551852 | KY627040 |
| **Phoebanthus grandiflorus** | FLAS: *Majure 5286* | MH552173 | KY627352 |
| **Phoradendron leucarpum** | FLAS: *Majure 4785* | MH551797 | KY626981 |
| **Photinia pyrifolia** | FLAS: *Majure 4630* | MH551656 | KY626831 |
| **Phyla nodiflora** | FLAS: *Majure 5237* | MH552127 | KY627305 |
| **Phyllanthus tenellus** | FLAS: *Majure 4740* | MH551755 | KY626937 |
| **Phyllanthus urinaria** | FLAS: *Majure 4719* | MH551735 | KY626917 |
| **Physalis arenicola** | FLAS: *Majure 5267* | MH552156 | KY627335 |
| **Physalis virginiana** | FLAS: *Majure 4978* | MH551920 | KY627107 |
| **Phytolacca americana** | FLAS: *Majure 5145* | MH552062 | KY627236 |
| **Pinus clausa** | FLAS: *Majure 4663* | MH551685 | KY626863 |
| **Pinus elliottii** | FLAS: *Majure 4758* | MH551772 | KY626955 |
| **Pinus palustris** | FLAS: *Majure 4566* |  | KY626769 |
| **Pinus taeda** | FLAS: *Majure 4837* | MH551811 | KY626999 |
| **Piptochaetium avenaceum** | FLAS: *Majure 4953* | MH551897 | KY627087 |
| **Piriqueta cistoides subsp. caroliniana** | FLAS: *Majure 5085* | MH552013 | KY627189 |
| **Pityopsis graminifolia var. latifolia** | FLAS: *Majure 4516* | MH551559 | KY626720 |
| **Plantago virginica** | FLAS: *Majure 4829* | MH551806 | KY626992 |
| **Platanus occidentalis** | FLAS: *Majure 5035* | MH551965 | KY627148 |
| **Pleopeltis polypodioides** | FLAS: *Majure 4692* |  | KY626891 |
| **Pluchea baccharis** | FLAS: *Majure 4571* | MH551607 | KY626774 |
| **Poa annua** | FLAS: *Majure 4833* | MH551810 | KY626996 |
| **Poinsettia heterophylla** | FLAS: *Whitten 4057* | MH552325 | KY627501 |
| **Polanisia tenuifolia** | FLAS: *Whitten 3921* | MH552193 | KY627372 |
| **Polygala setacea** | FLAS: *Whitten 3963* | MH552234 | KY627408 |
| **Polygonella gracilis** | FLAS: *Majure 4510* | MH551553 | KY626714 |
| **Polypogon monspeliensis** | FLAS: *Majure 5159* | MH552072 | KY627250 |
| **Polypremum procumbens** | FLAS: *Majure 4619* | MH551647 | KY626820 |
| **Pontederia cordata** | FLAS: *Majure 4704* | MH551720 | KY626902 |
| **Portulaca amilis** | FLAS: *Majure 5170* | MH552075 | KY627253 |
| **Portulaca pilosa** | FLAS: *Majure 4849* | MH551822 | KY627011 |
| **Prunus caroliniana** | FLAS: *Majure 5008* | MH551950 | KY627134 |
| **Prunus serotina** | FLAS: *Majure 4825* | MH551803 | KY626988 |
| **Prunus umbellata** | FLAS: *Majure 4821* | MH551799 | KY626984 |
| **Pseudognaphalium obtusifolium** | FLAS: *Majure 4562* | MH551599 | KY626766 |
| **Psilotum nudum** | FLAS: *Majure 5147* |  | KY627238 |
| **Pteridium aquilinum** | FLAS: *Majure 4845* |  | KY627007 |
| **Pterocaulon pycnostachyum** | FLAS: *Majure 4575* | MH551609 | KY626778 |
| **Pteroglossaspis ecristata** | FLAS: *Whitten 3912* | MH552183 | KY627362 |
| **Pyrrhopappus carolinianus** | FLAS: *Majure 4909* | MH551865 | KY627054 |
| **Pyrus communis** | FLAS: *Majure 4916* | MH551872 | KY627061 |
| **Quercus chapmannii** | FLAS: *Whitten 4002* | MH552271 | KY627446 |
| **Quercus geminata** | FLAS: *Majure 4667* | MH551689 | KY626867 |
| **Quercus hemisphaerica** | FLAS: *Majure 4606* | MH551635 | KY626807 |
| **Quercus incana** | FLAS: *Majure 4888* | MH551845 | KY627033 |
| **Quercus laevis X incana** | FLAS: *Majure 5210* | MH552114 | KY627292 |
| **Quercus margaretta** | FLAS: *Majure 4969* | MH551911 | KY627099 |
| **Quercus myrtifolia** | FLAS: *Majure 4891* | MH551848 | KY627036 |
| **Quercus nigra** | FLAS: *Majure 4626* | MH551652 | KY626827 |
| **Quercus pumila** | FLAS: *Whitten 4000* | MH552269 | KY627444 |
| **Quercus virginiana** | FLAS: *Majure 4605* | MH551634 | KY626806 |
| **Rhexia cubensis** | FLAS: *Majure 5296* | MH552185 | KY627364 |
| **Rhexia mariana** | FLAS: *Majure 5123* | MH552041 | KY627215 |
| **Rhexia nashii** | FLAS: *Whitten 3952* | MH552223 | KY627398 |
| **Rhododendron simsii** | FLAS: *Majure 4826* | MH551804 | KY626989 |
| **Rhus copallinum** | FLAS: *Whitten 3979* | MH552249 | KY627424 |
| **Rhynchosia difformis** | FLAS: *Majure 5121* | MH552040 | KY627214 |
| **Rhynchosia reniformis** | FLAS: *Majure 4524* | MH551567 | KY626728 |
| **Rhynchosia tomentosa var. mollissima** | FLAS: *Majure 4938* | MH551882 | KY627072 |
| **Rhynchospora fascicularis** | FLAS: *Majure 5259* | MH552148 | KY627327 |
| **Rhynchospora globularis var. globularis** | FLAS: *Majure 5133* | MH552051 | KY627225 |
| **Rhynchospora grayii** | FLAS: *Majure 4971* | MH551913 | KY627101 |
| **Rhynchospora megalocarpa** | FLAS: *Majure 4750* | MH551765 | KY626947 |
| **Rhynchospora microcephala** | FLAS: *Majure 5249* | MH552138 | KY627317 |
| **Rhynchospora pusilla** | FLAS: *Majure 5241* | MH552131 | KY627309 |
| **Rhynchospora scirpoides** | FLAS: *Majure 4585* | MH551617 | KY626788 |
| **Richardia brasiliensis** | FLAS: *Majure 4694* | MH551712 | KY626893 |
| **Rubus argutus** | FLAS: *Majure 4646* | MH551670 | KY626847 |
| **Rubus cuneifolius** | FLAS: *Majure 5056* | MH551986 | KY627166 |
| **Rubus trivialis** | FLAS: *Majure 4840* | MH551814 | KY627002 |
| **Rudbeckia mollis** | FLAS: *Majure 5234* | MH552124 | KY627302 |
| **Ruellia caroliniensis** | FLAS: *Majure 5120* | MH552039 | KY627213 |
| **Rumex hastatulus** | FLAS: *Majure 4896* | MH551853 | KY627041 |
| **Sabal etonia** | FLAS: *Majure 4892* | MH551849 | KY627037 |
| **Sabal palmetto** | FLAS: *Majure 4649* | MH551673 | KY626850 |
| **Sabatia grandiflora** | FLAS: *Majure 5202* | MH552106 | KY627284 |
| **Saccharum giganteum** | FLAS: *Majure 4616* | MH551644 | KY626817 |
| **Sacciolepis striata** | FLAS: *Majure 4709* | MH551725 | KY626907 |
| **Sagittaria isoetiformis** | FLAS: *Majure 5280* | MH552167 | KY627346 |
| **Sagittaria lancifolia subsp. media** | FLAS: *Majure 4614* | MH551642 | KY626815 |
| **Sagittaria latifolia** | FLAS: *Whitten 4030* | MH552298 | KY627474 |
| **Salvia azurea** | FLAS: *Whitten 4019* | MH552287 | KY627463 |
| **Salvia lyrata** | FLAS: *Majure 4994* | MH551936 | KY627120 |
| **Sassafras albidum** | FLAS: *Whitten 3990* | MH552259 | KY627435 |
| **Saururus cernuus** | FLAS: *Majure 4724* | MH551739 | KY626921 |
| **Schizachyrium sanguineum var. sanguineum** | FLAS: *Majure 4752* | MH551766 | KY626949 |
| **Schizachyrium scoparium var. stoloniferum** | FLAS: *Majure 4537* | MH551577 | KY626741 |
| **Schoenocaulon dubium** | FLAS: *Majure 4980* | MH551922 | KY627109 |
| **Scirpus cyperinus** | FLAS: *Majure 4726* | MH551741 | KY626923 |
| **Scleria ciliata var. ciliata** | FLAS: *Majure 4973* | MH551915 | KY627103 |
| **Scleria muelenbergii** | FLAS: *Majure 4592* | MH551621 | KY626794 |
| **Scleria reticularis** | FLAS: *Majure 5264* | MH552153 | KY627332 |
| **Scleria triglomerata** | FLAS: *Majure 4678* | MH551698 | KY626878 |
| **Scoparia dulcis** | FLAS: *Majure 4598* | MH551627 | KY626800 |
| **Scutellaria multiglandulosa** | FLAS: *Majure 4940* | MH551884 | KY627074 |
| **Scutellaria racemosa** | FLAS: *Majure 4990* | MH551932 | KY627117 |
| **Senna obtusifolia** | FLAS: *Majure 4708* | MH551724 | KY626906 |
| **Serenoa repens** | FLAS: *Majure 4736* | MH551751 | KY626933 |
| **Sericocarpus tortifolius** | FLAS: *Whitten 3958* | MH552229 | KY627403 |
| **Setaria magna** | FLAS: *Whitten 3946* | MH552217 | KY627396 |
| **Setaria parviflora** | FLAS: *Majure 4670* | MH551692 | KY626870 |
| **Seymeria cassioides** | FLAS: *Whitten 3938* | MH552209 | KY627388 |
| **Seymeria pectinata** | FLAS: *Whitten 3981* | MH552251 | KY627426 |
| **Sida rhombifolia** | FLAS: *Majure 4741* | MH551756 | KY626938 |
| **Sida ulmifolia** | FLAS: *Whitten 4044* | MH552312 | KY627488 |
| **Sideroxylon rufohirtum** | FLAS: *Majure 4939* | MH551883 | KY627073 |
| **Sideroxylon tenax** | FLAS: *Majure 4743* | MH551758 | KY626940 |
| **Silene antirrhina** | FLAS: *Majure 4937* | MH551881 | KY627071 |
| **Silphium compositum** | FLAS: *Majure 5275* | MH552164 | KY627343 |
| **Smilax auriculata** | FLAS: *Majure 5043* | MH551973 | KY627155 |
| **Smilax glauca** | FLAS: *Majure 4727* | MH551742 | KY626924 |
| **Smilax laurifolia** | FLAS: *Majure 4638* | MH551662 | KY626839 |
| **Smilax pumila** | FLAS: *Majure 4772* | MH551785 | KY626969 |
| **Smilax walteri** | FLAS: *Majure 5262* | MH552151 | KY627330 |
| **Solanum americanum** | FLAS: *Majure 5117* | MH552036 | KY627210 |
| **Solanum chenopodioides** | FLAS: *Majure 4713* | MH551729 | KY626911 |
| **Solanum viarum** | FLAS: *Majure 4964* | MH551907 | KY627095 |
| **Solidago arguta var. caroliniana** | FLAS: *Whitten 4020* | MH552288 | KY627464 |
| **Solidago odora var. chapmanii** | FLAS: *Majure 4543* | MH551582 | KY626747 |
| **Soliva sessilis** | FLAS: *Majure 4877* | MH551835 | KY627024 |
| **Sorghastrum nutans** | FLAS: *Majure 5257* | MH552146 | KY627325 |
| **Sorghastrum secundum** | FLAS: *Majure 4521* | MH551564 | KY626725 |
| **Spartina bakeri** | FLAS: *Majure 4890* | MH551847 | KY627035 |
| **Spermolepis divaricata** | FLAS: *Majure 5041* | MH551971 |  |
| **Spermolepis echinatus** | FLAS: *Majure 5059* | MH551989 | KY627168 |
| **Sphenopholis obtusata** | FLAS: *Majure 4831* | MH551808 | KY626994 |
| **Spiraea fritschiana var. parvifolia** | FLAS: *Majure 4917* | MH551873 | KY627062 |
| **Spiranthes tuberosa** | FLAS: *Majure 5265* | MH552154 | KY627333 |
| **Sporobolus clandestinus** | FLAS: *Majure 4695* | MH551713 | KY626894 |
| **Sporobolus diandrus** | FLAS: *Majure 4669* | MH551691 | KY626869 |
| **Sporobolus indicus** | FLAS: *Majure 4659* | MH551681 | KY626859 |
| **Sporobolus junceus** | FLAS: *Majure 4514* | MH551557 | KY626718 |
| **Stachys floridana** | FLAS: *Majure 5050* | MH551980 |  |
| **Stellaria media** | FLAS: *Majure 4832* | MH551809 | KY626995 |
| **Stenotaphrum secundatum** | FLAS: *Majure 5212* | MH552116 | KY627294 |
| **Stillingia sylvatica** | FLAS: *Majure 4551* | MH551588 | KY626755 |
| **Stipulicida setacea var. setacea** | FLAS: *Majure 5101* | MH552028 | KY627202 |
| **Stylisma patens** | FLAS: *Majure 4545* | MH551583 | KY626749 |
| **Stylodon carneum** | FLAS: *Majure 5127* | MH552045 | KY627219 |
| **Stylosanthes biflora** | FLAS: *Majure 5077* | MH552006 | KY627182 |
| **Symphyotrichum concolor var. concolor** | FLAS: *Majure 4540* | MH551579 | KY626744 |
| **Symphyotrichum dumosum** | FLAS: *Majure 4730* | MH551745 | KY626927 |
| **Symphyotrichum elliottii** | FLAS: *Majure 4615* | MH551643 | KY626816 |
| **Symphyotrichum undulatum** | FLAS: *Majure 4979* | MH551921 | KY627108 |
| **Sysyrinchium rosulatum** | FLAS: *Majure 5026* | MH551957 | KY627141 |
| **Taxodium ascendens** | FLAS: *Majure 4625* |  | KY626826 |
| **Tephrosia chrysophylla** | FLAS: *Majure 5095* | MH552023 | KY627198 |
| **Tephrosia florida** | FLAS: *Majure 5074* | MH552003 |  |
| **Tephrosia mohrii** | FLAS: *Majure 4760* |  | KY626958 |
| **Tephrosia virginiana** | FLAS: *Majure 5096* | MH552024 | KY627199 |
| **Tillandsia bartramii** | FLAS: *Majure 4658* | MH551680 |  |
| **Tillandsia recurvata** | FLAS: *Majure 4601* | MH551630 | KY626803 |
| **Tillandsia usneoides** | FLAS: *Majure 4602* | MH551631 |  |
| **Toxicodendron pubescens** | FLAS: *Majure 5269* | MH552158 | KY627337 |
| **Toxicodendron radicans** | FLAS: *Majure 4904* | MH551860 | KY627049 |
| **Tradescantia ohiensis** | FLAS: *Majure 5271* | MH552160 | KY627339 |
| **Tradescantia roseolens** | FLAS: *Majure 5110* | MH552029 | KY627203 |
| **Tragia urens** | FLAS: *Majure 4558* | MH551595 | KY626762 |
| **Triadenum virginicum** | FLAS: *Majure 4632* |  | KY626833 |
| **Triadica sebifera** | FLAS: *Majure 5070* | MH551999 | KY627177 |
| **Trichostema dichotomum** | FLAS: *Majure 4739* | MH551754 | KY626936 |
| **Trichostema setaceum** | FLAS: *Whitten 4024* | MH552292 | KY627468 |
| **Trifolium campestre** | FLAS: *Majure 5060* | MH551990 | KY627169 |
| **Trifolium repens** | FLAS: *Whitten 4050* | MH552318 | KY627494 |
| **Triodanis perfoliata** | FLAS: *Majure 4910* | MH551866 | KY627055 |
| **Triplasis americana** | FLAS: *Majure 4549* | MH551587 | KY626753 |
| **Typha latifolia** | FLAS: *Majure 5187* | MH552092 | KY627270 |
| **Urochloa ramosa** | FLAS: *Majure 5171* | MH552076 | KY627254 |
| **Urtica chamaedryoides** | FLAS: *Majure 5034* | MH551964 | KY627147 |
| **Utricularia floridana** | FLAS: *Whitten 4018* | MH552286 | KY627462 |
| **Utricularia gibba** | FLAS: *Majure 4699* | MH551716 | KY626898 |
| **Vaccinium arboreum** | FLAS: *Majure 4683* | MH551703 | KY626883 |
| **Vaccinium corymbosum** | FLAS: *Majure 4923* | MH551878 | KY627068 |
| **Vaccinium myrsinites** | FLAS: *Majure 4883* | MH551841 | KY627028 |
| **Vaccinium stamineum** | FLAS: *Majure 4664* | MH551686 | KY626864 |
| **Verbena officinalis ssp. halei** | FLAS: *Whitten 4012* | MH552280 | KY627456 |
| **Verbena scabra** | FLAS: *Whitten 4017* | MH552285 | KY627461 |
| **Verbesina heterophylla** | FLAS: *Majure 5291* | MH552178 | KY627357 |
| **Vernonia angustifolia** | FLAS: *Whitten 3970* | MH552240 | KY627415 |
| **Veronica arvensis** | FLAS: *Majure 4839* | MH551813 | KY627001 |
| **Viburnum nudum** | FLAS: *Majure 4965* | MH551908 | KY627096 |
| **Viburnum rufidulum** | FLAS: *Majure 5006* | MH551948 | KY627132 |
| **Vicia tetrasperma** | FLAS: *Majure 4991* | MH551933 |  |
| **Viola palmata** | FLAS: *Majure 5071* | MH552000 | KY627178 |
| **Viola sororia** | FLAS: *Majure 4881* | MH551839 | KY627026 |
| **Vitis aestivalis** | FLAS: *Majure 5151* | MH552067 | KY627242 |
| **Vitis rotundifolia** | FLAS: *Majure 4640* | MH551664 | KY626841 |
| **Vittaria lineata** | FLAS: *Whitten 3989* |  | KY627434 |
| **Vulpia octoflora** | FLAS: *Majure 4912* | MH551868 | KY627057 |
| **Wahlenbergia marginata** | FLAS: *Majure 4620* | MH551648 | KY626821 |
| **Woodwardia areolata** | FLAS: *Majure 4698* |  | KY626897 |
| **Woodwardia virginica** | FLAS: *Majure 4635* |  | KY626836 |
| **Xyris ambigua** | FLAS: *Majure 4581* | MH551615 | KY626784 |
| **Xyris baldwiniana** | FLAS: *Majure 5124* | MH552042 | KY627216 |
| **Xyris caroliniana** | FLAS: *Majure 5066* |  | KY627174 |
| **Youngia japonica** | FLAS: *Majure 4851* | MH551824 | KY627013 |
| **Yucca aloifolia** | FLAS: *Majure 5001* | MH551943 | KY627127 |
| **Yucca filamentosa** | FLAS: *Whitten 3930* | MH552201 | KY627381 |
| **Zamia pumila** | FLAS: *Majure 4751* |  | KY626948 |
| **Zanthoxylum clava-herculis** | FLAS: *Majure 5047* | MH551977 | KY627159 |
| **Zornia bracteata** | FLAS: *Majure 5175* | MH552080 | KY627258 |
